# Genomic Evidence of Recombination in the Basidiomycete *Wallemia mellicola*

**DOI:** 10.3390/genes10060427

**Published:** 2019-06-04

**Authors:** Xiaohuan Sun, Cene Gostinčar, Chao Fang, Janja Zajc, Yong Hou, Zewei Song, Nina Gunde-Cimerman

**Affiliations:** 1 China National GeneBank, BGI-Shenzhen, Jinsha Road, Shenzhen 518120, China; sunxiaohuan@genomics.cn (X.S.); fangchao@genomics.cn (C.F.); houyong@genomics.cn (Y.H.); songzewei@genomics.cn (Z.S.); 2 BGI-Shenzhen, Beishan Industrial Zone, Shenzhen 518083, China; 3 Department of Biology, Biotechnical Faculty, University of Ljubljana, 1000 Ljubljana, Slovenia; janja.zajc@bf.uni-lj.si (J.Z.); nina.Gunde-Cimerman@bf.uni-lj.si (N.G.-C.); 4 Lars Bolund Institute of Regenerative Medicine, BGI-Qingdao, Qingdao 266555, China; 5 Department of Biotechnology and Systems biology, National Institute of Biology, 1000 Ljubljana, Slovenia

**Keywords:** population genomics, halotolerance, xerotolerance, basidiomycete, allergenic fungus, recombination

## Abstract

One of the most commonly encountered species in the small basidiomycetous sub-phylum Wallemiomycotina is *Wallemia mellicola*, a xerotolerant fungus with a widespread distribution. To investigate the population characteristics of the species, whole genomes of twenty-five strains were sequenced. Apart from identification of four strains of clonal origin, the distances between the genomes failed to reflect either the isolation habitat of the strains or their geographical origin. Strains from different parts of the world appeared to represent a relatively homogenous and widespread population. The lack of concordance between individual gene phylogenies and the decay of linkage disequilibrium indicated that *W. mellicola* is at least occasionally recombining. Two versions of a putative mating-type locus have been found in all sequenced genomes, each present in approximately half of the strains. *W. mellicola* thus appears to be capable of (sexual) recombination and shows no signs of allopatric speciation or specialization to specific habitats.

## 1. Introduction

Towards the end of the 19th century, fish inspector Wallem was trying to tackle the problem of salted drying fish being spoiled by microbial growth [1]. From his samples in 1887, mycologist Johan Olav Olsen isolated and described the fungus *Wallemia ichthyophaga* [2]. More than a century later, and after several nomenclature changes, the only recognized species of *Wallemia* was *Wallemia sebi*. In 2005, the name *W. ichthyophaga* was resurrected for a group of *Wallemia* spp. strains able to grow only in media with substantially lowered water activity and an additional species—*W. muriae*—was described [3]. In 2015, a multi-locus phylogenetic analysis led to the description of additional species, *W. mellicola*, *W. canadensis*, *W. tropicalis* [4], followed by a description of *W. hederae* the following year [5] and finally *W. peruviensis* a year later [6]. In the resulting taxonomy *W. sebi s. str.* and *W. mellicola* were the most commonly isolated and most ubiquitous species of the genus. In addition to differences in molecular taxonomic markers, *W. mellicola* can be recognized by the larger size of conidia compared to *W. sebi*, while it is also less salt-tolerant and chaotolerant [4].

Due to their unusual morphology, *Wallemia* spp. long evaded reliable positioning into the fungal tree of life. The use of molecular phylogenetics showed that the genus is distant from all other known fungi, but its exact phylogenetic position remained uncertain. The first comprehensive molecular study by Zalar et al. [3] placed the *Wallemia* spp. into a new order (Wallemiales) and class (Wallemiomycetes) at the base of the Basidiomycota phylogenetic tree. Additional molecular analyses based on six genes confirmed a basal position of Wallemiomycetes to all of Pucciniomycotina, Ustilaginomycotina and Agaricomycotina [7]. Following the genome sequencing of *W. mellicola* and *W. ichthyophaga*, the analyses based on larger datasets positioned Wallemiomycetes as a sister group of Agaricomycotina [8,9]. Finally, the class Wallemiomycetes was accommodated in a new sub-phylum Wallemiomycotina, which was estimated to have emerged almost half a billion years ago, while its position in this study (as a sister group of just Agaricomycotina or basal to all three major subphyla of Basidiomycota) was again unclear and depended on the dataset used for inferring the phylogenetic relationships [10].

*Wallemia* spp. used to be known mainly as contaminants of food preserved with low-water-activity [3,11,12]. Later it became clear that they are frequent in both indoor and outdoor environments. They have been found in indoor air and house dust [13,14] and were reported to represent a large share of the microbiome of some species of house dust mites [15]. In natural environments *Wallemia* spp. are isolated particularly often from habitats characterized by low water activity [5]. While only a few isolates are known for some of the species of the genus, *W. mellicola* is encountered much more frequently. It can be found in different habitats around the world, among them air and house dust, hypersaline water of solar salterns, soil, salted, food preserved with low water activity, plant surface and pollen, straw and seeds [1]. These habitats reflect the extremotolerant character of *Wallemia* spp. Although tolerance of low water activity, especially if induced by high concentrations of salt, is rare among basidiomycetes, *Wallemia* spp. are among the most xerotolerant fungal taxa described to date, and some of them are even xerophilic—requiring low water activity to grow—an exceedingly rare trait in the fungal kingdom [3,5,16]. While *W. mellicola* is not the most extreme of *Wallemia* spp. in terms of halotolerance, the upper salinity levels supporting its growth are still high: 4.1 M NaCl and 1.4 M MgCl_2_ [1]. However, even though its growth optimum is at water activity of 0.97 to 0.92, *W. mellicola* also grows well in regular mycological media without additional osmolytes and is therefore considered to be xerotolerant/halotolerant rather than xerophilic/halophilic [4].

Strains of *W. mellicola* are known to produce secondary metabolites, namely tricyclic dihydroxysesquiterpenes wallimidione, walleminone, walleminol, and two azasteroids with antitumor activity, UCA 1064-A and UCA 1064-B [17]. Unusually, the production of wallimidione increases with increasing concentration of salt up to 2.6 M NaCl. This trait raises questions about the safety of salt-preserved food contaminated with mycotoxigenic *Wallemia mellicola* and other *Wallemia* spp. [17]. Walleminol (known also as walleminol A) was detected in food [18]. There are also sporadic reports of human infections by *Wallemia* spp. [19], although these may be underreported due to slow growth of the species [1].

Despite the above, the major threat posed by *Wallemia* spp. appears to be their allergenic potential, either through exposure by inhalation or, as shown by recent research, by the overgrowth of *W. mellicola* in the gastrointestinal tract. *Wallemia* spp. have long been associated with the development of farmer’s lung disease, a type of bronchial asthma or hypersensitivity pneumonitis (reviewed in [1]). A survey of air in animal and hay barns detected propagules of *Wallemia* spp. reaching up to 500 × 10^6^ colony forming units (CFU)/m^3^, while only 20 to 500 CFU/m^3^ were found in residential buildings [5]. Immune sensitization to *Wallemia* spp. is frequently observed in asthmatic patients. Species of *Wallemia* were among the few fungi that increased the risk of asthma for inhabitants of homes damaged by water [20,21]. 

*Wallemia* spp. are often found in the human (and mice) gastrointestinal mycobiota. In mice the eradication of *Candida* spp. with antifungals leads to gastrointestinal overgrowth of *W. mellicola*, *Aspergillus amsteoldami*, and *Epicoccum nigrum*. While feeding healthy mice with these fungi did not lead to changes in their gut mycobiota, oral administration of *W. mellicola* after transient antibiotic therapy led to expansion of *W. mellicola* in the gut (a phenomenon not observed for either *A. amstelodami* or *E. nigrum*). This expansion in turn led to altered pulmonary immune responses to inhaled aeroallergens–without *Wallemia* present in the lungs [22,23].

The genome of *W. mellicola* (strain CBS 633.66, isolated from date honey and at the time classified as *W. sebi*) was published in 2012 [8]. The genome turned out to be unusually compact for a basidiomycete (9.8 Mbp) and contained a putative mating-type locus, even though sexual reproduction in *W. mellicola* has not been described to date.

To investigate the intraspecific relationships between strains of *W. mellicola* isolated from various indoor and outdoor environments in different parts of the world, we sequenced the whole genomes of 25 strains and analysed them using population and comparative genomic tools.

## 2. Materials and Methods

### 2.1. Culture, Medium, Growth Conditions and DNA Isolation

Twenty-five strains of *W. mellicola* (Table 1) were obtained from the Ex Culture Collection of the Department of Biology, Biotechnical Faculty, University of Ljubljana (Slovenia). They were cultivated and their DNA was isolated as described previously [24]. All strains used in this study are publicly available in the Ex Culture Collection under their EXF numbers (Table 1).

### 2.2. Genome Sequencing

The genome sequencing was performed using the platform BGISEQ-500, with 2 × 150 bp libraries, prepared as described previously [25]. Multiplexing of the samples was used, and after demultiplexing, the quality of the reads was investigated using FastQC. Trimming the reads for adaptors and quality (removal of bases with Q < 20) was performed with the ‘bbduk’ script (https://jgi.doe.gov/data-and-tools/bbtools/).

The sequencing reads, assembly and annotation data have been deposited in Genbank under BioProject PRJNA527769 and in CNGB Nucleotide Sequence Archive (CNSA) (https://db.cngb.org/cnsa/) of China National GeneBank DataBase (CNGBdb) with accession code CNP0000446.

### 2.3. Variant Calling

Sequencing reads were mapped to the reference *W. mellicola* genome of strain CBS 633.66 (GenBank AFQX00000000.1) [8] with ‘bwa mem’, using the default parameters. This was followed by sorting with Samtools 1.6 [26], and identification of duplicates with Picard 2.10.2. Variant calling was performed with Genome Analysis Toolkit 4.1 [27]. ‘Genome Analysis Toolkit (GATK) Best Practices’ were modified by using the ‘hard filtering’ and haploid ploidy.

### 2.4. Assembly and Annotation

IDBA-Hybrid 1.1.3 [28] was used to assemble the genomes. The process was guided by the *W. mellicola* CBS 633.66 reference genome [8]. The other parameters were: maximum k-value 120, seed kmer 20, minimum support 2, similarity for alignment 0.95, maximum allowed gap in the reference 100, minimum size of contigs 500.

Protein-coding genes were annotated with MAKER 2.31.8 [29]. The published predicted proteome of *W. mellicola* CBS 633.66 [8] and the fungal proteins of the Swissprot database (downloaded on 12.06.2018) were used as evidence. Semi-HMM-based Nucleic Acid Parser (SNAP) [30] was trained in three bootstrap iterations (*W. mellicola* CBS 633.66 proteins were used as evidence in the first iteration, *W. mellicola* and Swissprot database in the second and third), using protein-alignment-derived gene models following the workflow of Campbell et al. (2014). Augustus predictions with the *Laccaria bicolor* training parameters was also used [31].

BUSCO 3 software [32] in proteomic mode and with the Basidiomycota protein dataset [33] was used to investigate the genome assembly and gene prediction completeness. All of the parameters were used as the default values.

Genome Annotation Generator (GAG) 2.0.1 software [34] was used to prepare the files for submission to GenBank. All of the gene models with a coding region <150 bp or with introns <10 bp were removed.

### 2.5. Variant-Based Analysis

Principal component analysis of the Single Nucleotide Polymorphism (SNP) data was performed with the ‘glPca’ function from the ‘adgenet’ package [35]. Linkage Disequilibrium (LD) was estimated on a dataset of biallelic SNP loci. For each pair of loci, the normalized coefficient of LD (*D’*) and the squared correlation coefficient (*r*^2^) were calculated using ‘vcftools’ [36]. To investigate LD decay, *D’* and *r*^2^ of loci within 10,000 nucleotides from each other were plotted as a function of distance and a generalized additive model fitted curve was added using ‘ggplot2’ in R [37,38]. The LD decay range was determined as the interval outside which all of the arithmetic means of *D’* or *r*^2^ were either higher (left interval border) or lower (right interval border) than half of the maximum observed *D’* or *r*^2^ means.

### 2.6. Phylogenetic Analysis

Gene phylogenetic trees were constructed from the predicted coding sequences of complete and single-copy BUSCOs. Alignment was calculated with MAFFT 7.407 in ‘--auto’ mode [39]. Gblocks 0.91 was employed to optimize the alignment, with the options ‘-b3 = 10 -b4 = 3 -b5 = n′ [40]; if the resulting alignment length was > 200 nt and the mean number of different nucleotides between the sequence pairs was larger than 15 (as counted by the ‘infoalign’ tool included in EMBOSS 6.6.0.0 [41]), phylogeny was inferred from the alignment with PhyML 3.1 [42]. The nucleotide substitution model was Hasegawa-Kishino-Yano 85 [43], the proportion of invariable sites and the alpha parameter of the gamma distribution of substitution rate categories were estimated by PhyML. Trees were visualized in DensiTree 2.2.5 [44]. A majority rule consensus tree was constructed in R with the function ‘consensus.edges’ (package ‘phytools’), using the default parameters [38,45].

The phylogenetic network was reconstructed from the SNP data as described previously [24].

### 2.7. Core Genome, GO Enrichment

The core genome *W. mellicola* was estimated with the pipeline GET_HOMOLOGUES 3.0.8 [46] from the predicted proteomes of all here sequenced strains and the reference strain *W. mellicola* CBS 633.66 [8] as a consensus of COGtriangle and OrthoMCL algorithms using default parameters. Representative sequences of each protein cluster were annotated using the PANTHER HMM scoring tools 2.1 and the HMM library version 13.1 [47]. Statistically significant enrichment of GO-Slim Biological Process terms was investigated at www.pantherdb.org for the lists of core gene clusters (present in all 26 genomes) and soft core gene clusters (in at least 24 genomes) with a list of all gene clusters used as a reference. Fisher’s Exact test and the False Discovery Rate correction were used.

### 2.8. Mating-Type Loci

BLAST was used to search for mating genes in the assembled and annotated *W. mellicola* genomes and predicted proteomes, using homologues of putative mating genes identified in the reference genome [8] as queries. The functional annotations of the genes were assigned according to Padamsee et al. [8]. Putative mating loci and their flanking regions were visualized in R with ‘ggplot2’ [37,38]. The corresponding regions of the genomes were aligned and the alignments visualized using Mummer 3.23 [48].

## 3. Results

*Wallemia mellicola* has a worldwide distribution and, unlike most other species in the genus, it is frequently isolated. To investigate its population structure, 25 genomes of *W. mellicola* were sequenced and compared. Strains were selected to cover a variety of habitats (from hypersaline water and various low water activity food to house dust, air, soil, plants and soil) and isolation locations (14 countries), as listed in Table 1.

Genomes were sequenced at 318× average coverage and the minimum coverage was 194× in case of genome 5. Using the reference *W. mellicola* to guide the assembly process, the genomes were assembled into 202 to 422 contigs (average 239 ± 43 SD). The size of the genomes was small and similar between the strains (average 9.75 Mbp ± 0.05 Mbp SD). Nevertheless, the completeness of the genome was high, with 88.18% (±0.50% SD) complete basidiomycetous Benchmarking Universal Single-Copy Orthologues (BUSCOs) present in the predicted proteomes, most of them in a single copy, and only 5.89% (±0.38% SD) of BUSCOs missing entirely. Between 4327 and 4509 (average 4475 ± 37 SD) predicted genes covered 66.64% (±0.32% SD) of the assemblies. The average intron length of 64 and the average GC content of 39.95% were very similar between the individual genomes (Table 2, Supplementary Table S1).

All 25 genomes and the reference *W. mellicola* genome shared 2845 genes (the core genome, identified by the GET_HOMOLOGUES pipeline). The softcore genome (genes present in at least 24 of 26 genomes) contained additional 611 genes. When the genomes were classified with the PANTHER classification system, the following categories were identified as significantly overrepresented in the core genome: Molecular Function: nucleoside-triphosphatase activity, oxidoreductase activity, protein binding, transporter activity, transferase activity, nucleic acid binding; Biological Process: nucleic acid metabolic process, cellular localization, transport, cellular component organization, gene expression, regulation of biological process; Cellular Component: chromosomal part, endomembrane system, cytosol, plasma membrane, vacuole, protein-containing complex, nuclear lumen.

**Figure 1:**
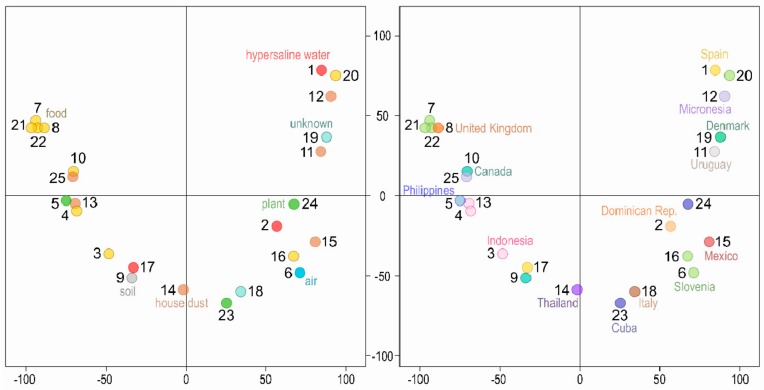
Clustering of the *Wallemia mellicola* genomes. Principal component analysis of single nucleotide polymorphisms (SNP) data estimated by comparing 25 sequenced genomes to the reference genome. The genomes are represented by circles, the color of which corresponds to the habitat (left) or sampling location (right) of the sequenced strains. The first two axes explain 23.7% (*x* axis) and 8.80% (*y* axis) of variation.

The density of single nucleotide polymorphisms (SNPs) when compared to the reference *W. mellicola* genome (Table 2, Supplementary Table S1) was very similar for all strains (0.41–0.60%). No strains with very high similarity to the reference genome were found. There was also little clustering of the strains in the principal component analysis (PCA) of SNP data (Figure 1). The first two axes explained 23.7% and 8.80% of the variation. A cluster of four strains isolated from food (three from Slovenia, one from United Kingdom) were observed and were most likely of clonal origin (Supplementary Table S2). Two pairs of strains from the same country clustered closely together (4 and 13, 6 and 16) but apart from that the strains from the same habitats or from the same geographic locations were spread relatively far from each other on the PCA plot.

**Figure 2:**
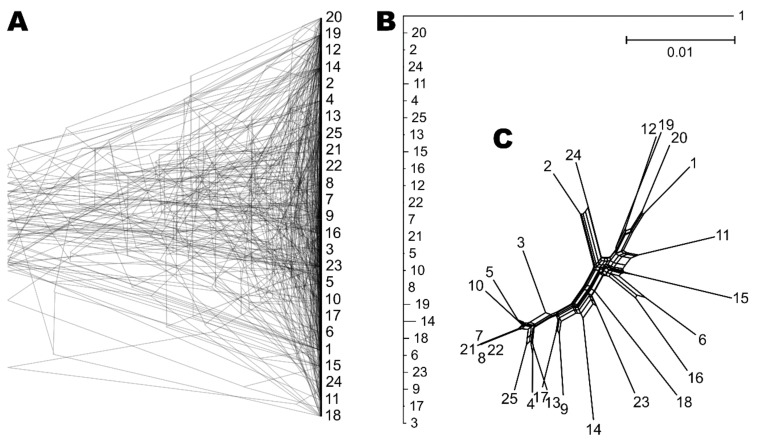
Phylogeny of *W. mellicola* strains. (**A**) Overlay of 40 core Benchmarking Universal Single-Copy Orthologue (BUSCO) gene trees estimated by PhyML 3.1 using the Hasegawa-Kishino-Yano 85 nucleotide substitution model and estimating the alpha parameter of the gamma distribution of the substitution rate categories and the proportion of invariable sites. (**B**) Majority rule consensus tree of 40 core gene trees described above. (**C**) Phylogenetic network reconstructed with the Neighbor-Net algorithm based on the dissimilarity distance matrix calculated from the SNP data.

Phylogenetic analysis of core BUSCOs (40 single-copy BUSCOs present in all sequenced genomes and with a minimum average of 15 different nucleotides between gene pairs) returned very different phylogenetic trees (Figure 2). There was little concordance between the tree topologies, resulting in a majority rule consensus tree with an extreme, star-like topology. The phylogenetic network analysis of the SNP data detected a fair amount of reticulation in the network.

**Figure 3:**
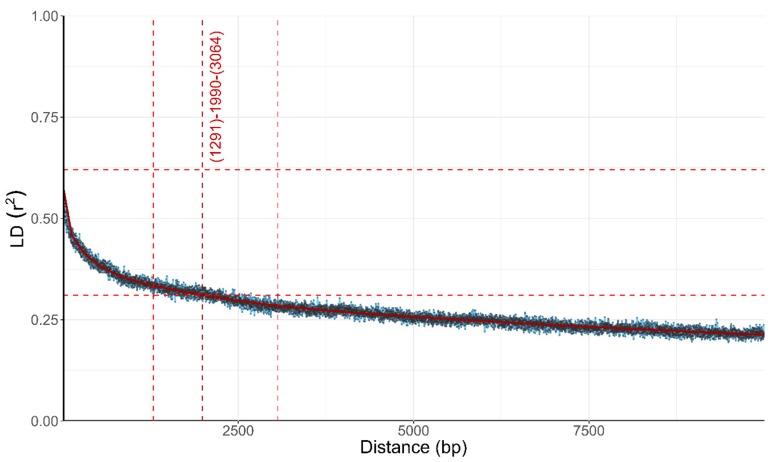
Linkage disequilibrium (LD) decay in *Wallemia mellicola* estimated by calculation of the squared correlation coefficient (*r*^2^) between pairs of biallelic loci. *r*^2^ is plotted against the physical distance of the loci in the genome. Horizontal lines mark the maximum observed value and half of the maximum observed value. Vertical lines mark the interval of the physical distance delimited by the first point of the curve under half of the maximum *r*^2^ value (left vertical line), the last point above half of the maximum *r*^2^ value (right vertical line) and the point where the fitted curve intersects with half of the maximum *r*^2^ value (middle vertical line).

A lack of concordance between phylogenetic histories of individual parts of the genome can be best explained by recombination shuffling these parts of the genome between individual organisms. A well-recognized estimator of the amount of recombination is the influence of the physical distance between the loci on the linkage disequilibrium (LD) between these loci. In non-recombining organisms the linkage between the loci should be absolute, no matter the distance between them, while in recombining populations the linkage between two loci is expected to decrease as a function of the distance between the two loci, approaching (but not necessarily reaching) equilibrium. In *W. mellicola* the LD decay, the average distance over which the LD falls to half of its maximum value, was 1291–3064 bp (intersect of the fitted curve was at 1990) when estimated with the squared correlation coefficient (*r*^2^; Figure 3) and 224–860 when estimated with the normalized coefficient of LD (*D’*; data not shown).

**Figure 4:**
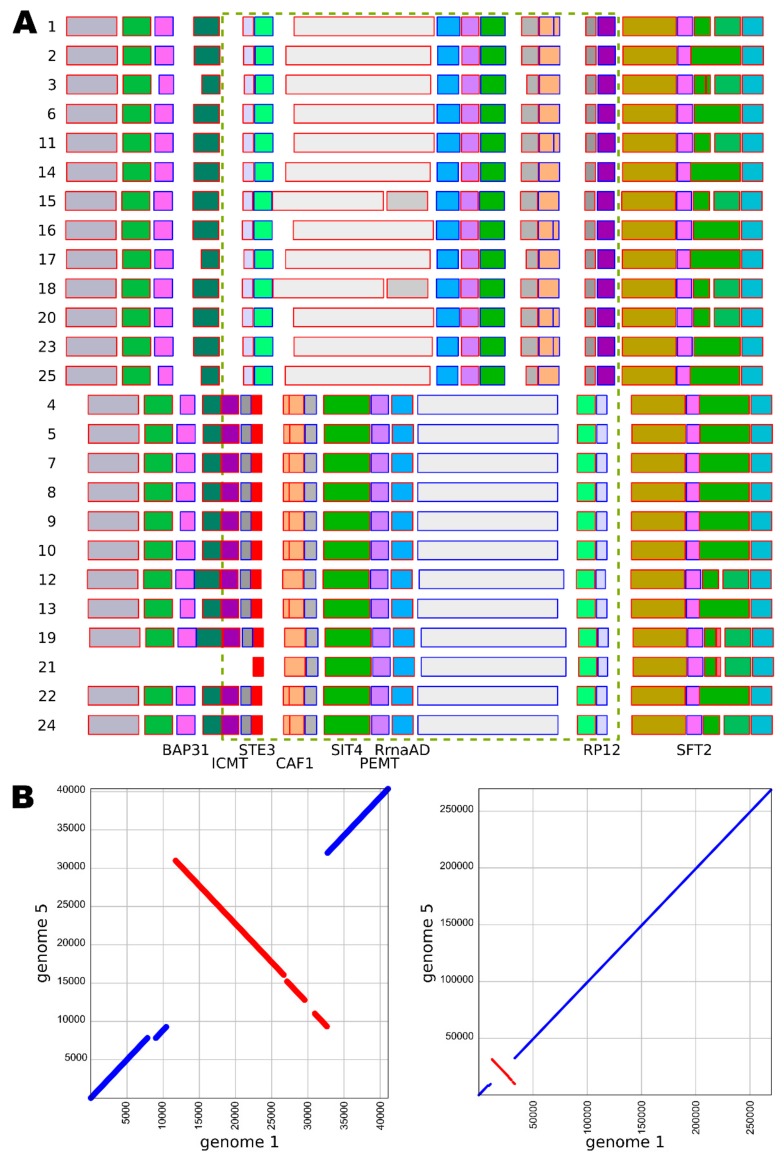
Putative mating-type loci in different strains of *Wallemia mellicola*. (**A**) Annotated mating-type loci and their flanking regions with putative gene functions assigned according to Padamsee et al. [8]. The chromosomal inversion is marked with a dashed green rectangle. Genome numbers are marked on the left. The blue or red outline of the rectangles representing the genes shows the gene orientation: left to right (blue) or right to left (red). (**B**) Alignment of the contigs containing the putative mating-type loci from the genomes 1 (*x* axis) and 5 (*y* axis) at different magnifications.

A putative mating-type locus of *W. mellicola* that was identified by Padamsee et al. [8] was also found in all 25 here sequenced genomes (Figure 4, Supplementary File S1). Two groups of strains (containing 12 and 13 strains each) were recognized by comparing the gene annotations in the locus - the two groups differed in some of the genes and in the orientation of the locus. No other chromosomal inversions were identified in the corresponding contig (Figure 4).

## 4. Discussion

Some extremotolerant fungal species are found in a large number of diverse environments, presumably using their stress tolerance to endure the often challenging conditions in their chosen habitats [49,50,51]. Although an infrequently mentioned example of such species, *Wallemia mellicola*, a species described upon a taxonomic revision of *Wallemia sebi*, can be isolated from habitats as different as air and house dust, hypersaline water of solar salterns, soil, salted, food preserved with low water activity, plant surface and pollen, straw and seeds [1] and is even a common part of the human gastrointestinal mycobiota [23]. To compare *W. mellicola* isolates from a variety of habitats and locations, twenty-five strains (Table 1) were genome sequenced and compared to investigate the possible existence of local subpopulations or cryptic specialization towards specific habitats and to check for evidence of recombination within the species. Such result would not be unusual—in recent years population genomics enabled the discovery of several cases of cryptic diversification or specialization in fungal species, which had previously appeared homogenous due to the lower phylogenetic resolution of standard taxonomic markers [52,53,54,55].

The sequenced genomes showed high similarity to the reference *W. mellicola* genome, which was reported to be very compact and with a large gene density [8]. The genomes of *Wallemia* spp. thus remain among the smallest in Basidiomycota [56,57]. The twenty-five genomes sequenced here were on average 9.75 Mbp large (compared to 9.8 Mbp of the reference genome) and 4475 protein coding genes were annotated per genome—with very little variation between the genomes (Table 2, Supplementary Table S1). This was less than 5284 genes annotated in the reference genome, which might be a consequence of different approaches to the genome assembly (de novo for the reference genome and reference-guided from short reads here) and/or to the gene annotation. At the same time 94% of the Benchmarking Universal Single-Copy Orthologues (BUSCOs) were found in the genome on average, indicating that the genomes were assembled to a high degree of completeness.

The variation between genomes (0.52% average SNP density compared to the reference genome (Table 2, Supplementary Table S1)) was comparable to variation observed in other fungal species, for example 0.55% in wild strains of *Saccharomyces cerevisiae* [58], 0.41% in *Neurospora crassa* [59] or up to 0.66% in *Candida glabrata* [60]. Only one clonal cluster of nearly identical genomes was observed on the PCA plot of SNP data, representing strains 7, 8, 21 and 22, which differed by less than 500 SNPs per genome pair (Supplementary Table S2). All four strains were isolated from food, three from chocolate in Slovenia, one from bread in UK. The next most similar pair of genomes (4 and 13, both isolated in Indonesia) differed by 17223 SNPs. However, despite this there was no general trend of higher similarity based on geographical proximity—although only a rough estimate, the average number of SNPs between the isolates isolated in different countries and the average difference between the genomes from the same country differed by only 2.4%. Similarly, the PCA did not uncover any clustering of strains based on either their habitat or geographical origin (Figure 1). This indicates that adaptation of localized populations or cryptic specialization for specific (types of) habitats is not widespread in *W. mellicola*. Considering the many habitats *W. mellicola* is able to inhabit, such generalization is not self-evident. To name just a few examples, using genomic data, it was discovered that the mushroom *Suillus brevipes* shows strong population differentiation and environmental adaptation [61], the pathogen *Cryptococcus neoformans* diversified into lineages with different pathogenic potential [62], the arbuscular mycorrhizal fungus *Rhizophagus irregularis* diverged into four main genetic groups, which were not related to the geographical origin of the strains [63], and the yeast *Metschnikowia reukaufii* diverged into lineages, which were again not related to geographical origin, but were shown to be metabolically distinct [53].

The general absence of clear clonal clusters (apart from the above-mentioned exception) raised a question about signs of recombination within *W. mellicola* genomes. This would be in line with the proposal made by Padamsee et al. [8] that *W. mellicola* is capable of sexual reproduction and their description of a putative mating locus in its genome. The availability of the genome sequences of *W. mellicola* for the first time enabled us to check for evidence of recombination in the species. If an organism is recombining (either by meiotic recombination or through other mechanisms), different parts of its genome are expected to have different phylogenetic histories. Two main approaches are used to check this in practice. The first is to reconstruct the phylogenies of individual genes and compare them. If, on the one hand, the gene trees share the same topology, they fulfil the “strong phylogenetic signal” criterion for clonality [64]. If, on the other hand, there is a lack of concordance between the phylogenies, this can be interpreted as a good indication of recombination. In the case of *W. mellicola* the latter scenario was observed and the differences between the gene trees were so numerous that the majority rule tree had an extreme star-shaped topology (Figure 2). The second approach for investigating recombination on the genome level is to look at the inheritance of loci on the same DNA molecule. In clonal organisms two loci will always be inherited together—they will be linked and in maximum linkage disequilibrium (LD). In sexual organisms two loci can segregate randomly and even if they are on the same DNA molecule, the linkage between them can be broken by chromosomal crossover—which is more likely to happen the further apart the loci are located. Such decrease of LD with distance, and particularly the distance over which half of the maximum observed LD is reached (a value known as LD decay distance), is a good measure of the amount of recombination in the population [65]. In the sequenced strains of *W. mellicola* the LD decay distance calculated from biallelic SNPs was around 1990 bp (Figure 3). This value is higher than observed in many other fungi [55,58,66], but much lower than in highly clonal species, where LD decay distances can be larger by two orders of magnitude [66]. This indicates that *W. mellicola* is at least occasionally recombining, but also extensively combining this with clonal reproduction—an observation in line with the identification of four clones in the sequenced dataset. 

The indications for recombination in *W. mellicola* appear to support its proposed capability for sexual reproduction [8]. Indeed, the putative mating-type locus has been found in all 25 here sequenced genomes. Furthermore, the locus was found to exist in two variants, which share several regions of high similarity, but differ in their orientations relative to the rest of the contigs on which they are located, and which contain no other chromosomal inversions (Figure 4). These two variants possibly represent two different mating types of *W. mellicola*—a hypothesis to be tested in the future.

## 5. Conclusions

*Wallemia mellicola* is an extremotolerant basidiomycetous fungus from the sub-phylum Wallemiomycotina, with a distinct phylogenetic position and capable of inhabiting a wide variety of habitats. Sequencing and analysis of twenty-five *W. mellicola* genomes showed that the strains form no clusters based on the habitat or geographical location from which they were isolated. The sequenced strains appear to represent a relatively homogenous and widespread population with only one clonal lineage detected in the dataset. The lack of concordance between individual gene phylogenies and the decay of linkage disequilibrium indicated that *W. mellicola* is at least occasionally recombining. The mechanism of recombination could be sexual reproduction—two versions of a putative mating-type locus have been found in all sequenced genomes, each present in approximately half of the strains.

## Figures and Tables

**Table 1:** Strains sequenced in this study.

Culture Collection Strain Number *	Number in This Study	Isolation Habitat	Sampling Site Location
EXF-277	1	hypersaline saltern water	Spain
EXF-757	2	hypersaline saltern water	Dominican Republic
EXF-1274 (CBS 110588)	3	peanuts	Indonesia
EXF-1277 (CBS 110589)	4	*Channa striata* dried salted fish	Indonesia
EXF-1279 (CBS 110593)	5	straw hat	Philippines
EXF-5677	6	air	Slovenia
EXF-5829	7	chocolate	Slovenia
EXF-6156 (UAMH 2651)	8	moldy white bread	United Kingdom
EXF-6157 (UAMH 2757)	9	soil	Canada
EXF-6158 (UAMH 6689)	10	maple syrup	Canada
EXF-8738	11	house dust	Uruguay
EXF-8740	12	house dust	Micronesia
EXF-8747	13	house dust	Indonesia
EXF-8749	14	house dust	Thailand
EXF-8757	15	house dust	Mexico
EXF-10633	16	dry common fig	Slovenia
EXF-483	17	hypersaline saltern water	Spain
EXF-1262 (CBS 213.34)	18	Unknown	Italy
EXF-1443 (IBT 19078)	19	Unknown	Denmark
EXF-5828	20	chocolate	Slovenia
EXF-5830	21	chocolate	Slovenia
EXF-5922	22	chocolate	Slovenia
EXF-6152 (MUCL 45613)	23	forest plant (*Clusia rosea*)	Cuba
EXF-6151 (MUCL 45615)	24	forest plant (*Verbena officinalis*)	Cuba
EXF-8741	25	house dust	Micronesia

* EXF strain numbers (Ex Culture Collection of the Department of Biology, Biotechnical Faculty, University of Ljubljana, Slovenia); other culture collection numbers are in parentheses (CBS—CBS-KNAW culture collection, Netherlands; UAMH—UAMH Centre For Global Microfungal Biodiversity, Canada; IBT—IBT culture collection, DTU, Denmark; MUCL—BCCM/MUCL Agro-food & Environmental Fungal Collection, Belgium).

**Table 2:** Statistics for the sequenced *Wallemia mellicola* genomes.

Statistic *	Minimum **	Mean **	Maximum **	Standard Deviation**
Coverage	194	318	558	92
Genome assembly size (Mbp)	9.68	9.75	9.95	0.05
Number of contigs	202	239	422	43
Contig N50	115375	144560	170540	13888
GC content (%)	39.91%	39.95%	39.97%	0.01%
CDS total length (Mbp)	6.44	6.50	6.53	0.02
CDS total length (% of genome)	66.45%	66.64%	67.08%	0.32%
Gene models (n)	4317	4475	4509	37
CDS average length (bp)	1438	1453	1512	13
Exons per gene (average)	3.98	4.02	4.17	0.04
Intron average length (bp)	63	64	66	0.57
Complete BUSCOs	87.40%	88.18%	89.80%	0.50%
Complete and single-copy BUSCOs	85.90%	87.91%	88.60%	0.56%
Complete and duplicated BUSCOs	0.10%	0.27%	3.90%	0.76%
Fragmented BUSCOs	5.50%	5.94%	6.50%	0.31%
Missing BUSCOs	4.60%	5.89%	6.50%	0.38%
SNP density	0.41%	0.52%	0.60%	0.04%

* Complete data for each genome is available in Supplementary Table S1; ** Calculated from 25 here sequenced *W. mellicola* genomes; CDS, Coding Sequence; BUSCOs, Benchmarking Universal Single-Copy Orthologues; SNP, Single Nucleotide Polymorphism.

## Supplementary Materials

The following are available online at https://www.mdpi.com/2073-4425/10/6/427/s1, Table S1: Statistics of the sequenced W. mellicola genomes, Table S2: Number of different SNP loci between pairs of W. mellicola genomes, File S1: Sequence of the putative mating-type locus and flanking genomic regions.
