# Supplementary material for: Genomic Evidence of Recombination in the Basidiomycete Wallemia mellicola
Source: Genes (Basel). 2019 Jun 4;10(6):427. doi: 10.3390/genes10060427 (PMC6628117; doi:10.3390/genes10060427)
Supplement: Supplementary file 1 [file genes-10-00427-s001.zip › FileS1.doc]

Mating loci of *Wallemia mellicola* strains 1 and 5.

Upstream region in blue.

Mating locus in red. Region found in both mating locus types is underlined in Wm1.

Downstream region in green.

>Wm1

TACATTTTAGCTTTGTATAATAACAAGTAAGCCACACTATCTTCACCTCCACCTTCTAATTGCGCAATTTTCTCCTTTGATATCTTGCTAACCTTGTTATCATCGAATTTATACCAATCATCTTGGTGTTCTTCACCTTTGTGTACATCTCCCCTAACCCAACCGATGTAGTGACCACCATCTGCGTTGACACCTTTGTGTGTAACTATGCCAACCAAATCGTACAATCCACTCTTTGAAGAACCAATGTCATTCTTCAAGTCCTCATCAACTAACTCATCCAGTTCCTTCTTCTCCTTCGCTTGATATACTTCTTCACTGAGTAATTCTGCATCACCAATCTTGTTTTGTTCATCTTTTATCTCATCGATTAACATCTCATCATTGTTCTTTGAATTGTTAGCCTTCTCCTCCAAGTCGTTTCTCGTCTTGATGCGCTTCCTCAATCTTCTTCTTTCTTCTCTATCCTTGTTGATCGAATAAAGTTTCTTATTGACTGGATTGATCTTGGCTTTCAAATCGTCACTAACTAATTCGCTCGCATCGTACTCCATTGGGAATTTTACTTTACGGAGAATCTTGGCTTTTTTACCTAAGTCAGCTCTCCAGTAGAATCTCACCAGATGAACGGTAAGATAGCTTGGTAGACGATTTATTCTGGACTTCTGAACATATTGAGCGTTCCTGTTAAGAGTTCCAGAATATTTCTCAATTTGTTCATCTAAGCCTTCACTCAAGCCTTGTTGGAGGTAATTGGTATTTGAAGAGATATTACAGCCCAGTTTGGTGAATTCCTCGATCGAATAAGTAGGTTCTTCCTCTGGCGCTTCAGGTGTCGACATTTCTTTCACATAGCTACCTCTCATGTACTGATCGACAAATGACTTGCCTTCCATACCAGTGGTGCCTGCTAATGAGCTGAGTATCGCACCCCAGGCTTCTTCTGCATCTTGTTGGGCAAACTGTCCGAATCTGTCTCTCTCTGTGAACTGTTGGTAGTTTGATCTTAACGTTGTCAAGAATGAAGTTGGTGTTATTGCGTCAGTTGAGTTGTTAAGTGAGCTAAAAAGCGCTTCTAAGGATCTACTGAGACCATTGTTCTGTGAGTTGTTTAAGTTGCTTTGTAATTCCGGTATAGCTCTCAGAACCTGAATTGAACTGTTCAAATAACAAGTGTTACCAAGGTTTGTCAAACCAACTGGGTTCTTCAAGGCTTGTGACAACTGCTTATCATCGAGATCTTCCATGAATACGACCTTTTCTGTAGGCGCCGAAGGTAATGGACCTGCTGCACCAATAACCATGAACAAATGACCTGGTTTTATTGTTGATCCGAGCTTCTCCATATCAGTATCATCCTTTAAGATACCACCTTTTATCATAACTTTTTGTCTAAACTGAGTGTTAAATGAGGTATACTTCACTAAGGTTCTTCTGCTTACCTCTCTGGTGCCACGCCAGTTATAGCGTAGATATCATTCTTAAAATCTAAACCAGATGCTTGTGTATTTAAATCTAAATCTAATTTCTTCCCTGCGTGCTTAATTGATACTGGTATTTTAGTCATCTCTTCGTAGTAGTTAATAGAAAAATGACGCGTTCATTTAACTGTCAAATTATGTATTTTTCTGTGTAAAAACAATAATATGGATTTATTTATGCTATATTTAAAATGTAAAAATCAAAATATTCTAATCATCTCTAGATTAAAGACTCATTGAAATACTGACGTCAACATTTCGGTTGAACTCCTCTCTTCTACGAACGTCGATTATCAAAAATAAAAAACTAGAGAAGCAAAATGACAGTGAGTACTATTTTATAGCGTTATACTATTTTACTAAGACTCACTCTTTAGCTTTATTACAGCTTAGTGTGGGTAATCAACTATCAATTAGAGATAGATAATTAACAGTTTAAACAGTTTCGGTTTACTCTCTGCTGAGTACGCTTGATTCGTCATAATATCTATCAACTCATCACACTCATAGAATCGTCACTTTCTTGGTATTAGTAGCACCAATGCCATTTGCGTTTAAGCAAAAGTTCTTTAAATTTTTAAGCACAAACCCATTAGTTGCTAAATTACAATACTCCTTGAAGATCTCTCTCATATTCACTTCCATTCTCTTCTTTGATGCTGTACAAAGGTGAGTTTAAGTGTAATAATCAGAAATATCATTACTTACATTATCAATAGAATGCTTAGAGTCGTTAAAGAAGGTCAAACAGCCAAAGAGGAACATGCTTTCACAGATGTACGCTCAGAAACTAACTTTGCCGCACGCAAGTACGTCACTTTTATATTCTAAAGATCTAAATTATTCACACACTTTTTAGGTTTTACTCACAACGTAACGTTTATCTCACCGGTATGTCTCACCTTTCTTTTCGTCATCTTTACAAAACTCATTTTCAAAAGGTTTCACCCTCTTTCTCAGCTTGTGGGTATTACTGCTACGTTTTTAAATTAACCAACTAATTCATTCCTTCTTCCATTAGAATTCTTTCACGCGTATTTGGTATTATCATGGACCTTATTCAATCACAAGAAGAATTGGGTTTGATGAAGGTACTCATTATACAATTATATTTAAACAATTTACTTACATTCTTCATAGAAAGATGGCCAATCATCAGCTGCTCAAAACTTCAAGAAGCAAATTGAAGCTAAAGATCGTGAAATTGGTTAGTATTATTAATAAAACCTTTCACCTTATTTACAAGCTTTTCCAGAAATTCTCAAAAAGCAATCTACACAAAACAACAAAGCTATGAACGAACTCGTTGATAACGCTAATTCTTCATCAGGCTCAAAGAAGGAGAAGTAA

TGCAATTGTACAAGATGTATGTTGTTGATGACAGTAAATAATTCATAGTATGTTTGGTTGATGTTATAATTGTTCTACTTTATAGATTATTACGATAGTTTTGTAGATTAAAAACAACATTTGGGTCAACATAGTTAGAGTGATTCAGATTATCAATAAAAACCTTATCTTGAATAGTATATTCAGAAGAAGTATTCAACAATTCTGCTGTTAGTATCTTGTTTGGTAATGGGTCAACTACATCAAGAAGAGGCAATTGCAAATGTAAATATCCTTGTGAAAGAAGTAAGATTTTCAGTTGCTCTAAAGTAGTATTCATAGTGATAATTTCGCAATTAACTTGACTTACCTCGTAACATTTTATTCTCGTTTTCAAGCCTAGTGACCAGGTCTTTTTGGTTTTGACGATAACGTCGCCCAATCTCTAGATGATATTAAGCTACTGTAGGTAATCTGTGGCTTCATGAACTAACCTTGGCTTCTTTCAAATCGTTAGGAAAGCTCTGACTTTTTAAAAAGACTTACTTCGTAGCTGGACTTTGAGTGTTGCGAGGTCGACCACGACCTCTCGATACTATTGTTTTACTAGACATCATTAACTACCTTAGTTTCTATCAGAAAGTATATGTGATGATTGATATATCGAATATATGTACGCTCTGATCTGTCCGTCCGCATTCCTATCTGTACCTTTTTTTAAGACAATGAAACTAAAAAAAAAATCATGCGCTAAACAAAGATACCAGAAATAAAGTTACCATTGTTCTAAAAACTGACAATAACAATGGCGATAAATTTTTCAATGGTTAGCATTATAAATAAGCGTCTTTCTCTAATAAGCTTTATTGACATTAAAAGATGGAACCAGAGTCATTTAACAACTTCAACTGTCAAGATACAGACAAGTTCTATTCACAAAATGATGAAACCTACTTCGAAACTGGTATTGGAAGGACTGAAATGTCAAGTGAAGATTTAGCTAATTTCGATAATCCTATGGATGATGACACTGGTACAGTAGGTGATAAGGCTGACACCATATACTGTGTCATTGCTTAAGTTAGTTATTATCATTCAAAATTTATCATTACTAATTCATTAAACAGAAAATTCAGAATTTTGTGCAATGATTGTTCTTCAATATATATATATACATTATGCAAATAATTCAGGTGCTTGACTGTTTACATCGACGGCCACATTCTCTACTTTAGATCTCCATTCGTTTAGACGTGAAGCCAATGATTTGACTTGATTTTTATCGAGTACTCTTGGTTGTACCCAATTGATGCTAGCTGAGCTTGAGACTTGGTCAATAGTACCACGGATAAGTTTAAGCCTTATTATTTTAAGTTGAAACAATCCAGTAAATAGATTACTCACGATAATGCTTTCATAATTAAGTGTTCTACTTCATCAAGTGGAAGCTTAGTTTCAGCGCCGATCGTTGAGAAAGACATGTCACGGTCTTTTGATGACCTTTTGAACACGCATTCGATTAATGCCATAAGGCATATCTTTTGACGAAGGAAAGCTTGACTGTCGTGTAAGATTGGCTTATATAATGTTACTTTCAGTGTTCTAAAATGCTATTACACCCACTTCATTTGCCAATTTAGAGGCATTAACCTCAAACTTGGTTATATCACCTTCGTTGAAAATGTAAATAATATCTTTCAACCATTCTAATTCGGTTCCATCAAGAGCATGTAAGATTGGATGAGTCAACTGAATTTTTAATTAGAGAACTACTATATATTATATTAAAGTGAGTAAGCTTACCAATTCACCGAAATTGTAAATTGTATCACCTAATAAGGCAGCGATGGCTAAATCGTGACTTCTTTGGATTTGCTCAGCTTTGTCAAGTTGGTCCATACCAGCACATGAGATATACAGTAATGTTGTACGGTAAAATGCTGCATATTCCGCTTTTGACTGATTTTTGATTAGGTCGATTTCTGAAGAGTTCCAAATACTCACTTTAAAGTAATCACCACTAACGCGTAGCCAAGCACCAGTGACACTGGCTTCTACGCCATCAATCGAATCTAAAACACGCTCGCAAACATCCATAGCTTCTTTAGCTTTGTCTAAGTTACCAAGTAAAAGGTCATAATGTGCTATTGAGATTTTTGCTAAAACGTAAGCCTCAGATTCGGTTTTAGTTGGTGGTTGCTCCTCATCGAACGATTTTGTATTGGTTGGCTTCAGTGCAGGTGGTAATGCAGCTCTTGGAACTGGTAGGGTGTCCAATAAAGATTCTAAGAAGTTTAGAGACTTTTGTGGAATCATCTCCTGTGCTACTCTAACGCCAATTTCCACTAAACGTAGTTGATTCAACTTATCCTTGAATGTTGAGATAAAGTTATTCCACAAGTCAACCAAATACTTAGAAATATCATCCTTCTGTATGATTTCTTGTAGTGTTTTAGTAAGCTGATGGTATAAACTTTGGTGGCTGGTTAGCAATTATTTGATTCCTCGCGTAAGACGCACCGCTTGCTATAATTAATTTCTAATTTAGTCAAATCTTCTGTTAATGAAGCTGGCGCCTCTGCTTTTACTTTATTTATGAATTCTGTCGGACTCATGTTGCGCGTGCGTAATCGTTCTACTCAGGGCTCTGACCAACCCATTCACTGACGCAACGAAGCAATTCGATTTTTTATTTACTCTCTTCACAAAGAACTTTCAACTATTAGCAAATTAAAATAAATGTCATTCGCAGATATAAGTCAGATTGGTGATGGGCTTATTCATACCATCAATCAAAGCCTTCACAATCATCAAAGTAAACAAAGAGATGAAGCCAGTATACAGAATTATCCCGTCATACAGCTGCTCGACCACTTAAATGGCAAAAAACCTGTACATAGAGATAATGCTGTGATTGGTATTATAAAGAGTCTGCACAGTTTGGAGAACAATGGAATGCCTAAAAGCTGTATGGACGAAGTCAAGGAGGCGCTGAAGTATCAAGATTTAGTTAATGGATCATTTATTGCGTCTACGTGCAAATGGGCTGAAAACGGTGAAACTCTTGAACATAACATTGATGATAAGCATCGAAAGCAGGTATGTTACATAGTTTCTCAAGATAGAGGTTAATAACAAATGTAGGTCTTTCGCGAAATCTTGCCAGAATTGTACAAACAGAATCCTAGACCTTCAGTTGCTGACAGAAATTATTTGAGTCGAGTATGTTACCTTAGGGCCCGTGTTTCAAGCTAATATAAAGCTTAAGGTCACCGGTTTGGGTCGCAAATGCCTTGACACATTCGTTAGTTCTTTACATTTATGTCGTATACTAATTCAAAGTTTAATAGTTTCAAAATAAGAGAAACCGTCACGGTCAAACCAGAAAAAGAGTGTCATCTGTGCATTCATCAAATAGTGCAACTTCTCAAGTTGATATATTGTATGATGCGTCAGACAACGGGTAAGTATTGATATCTATAATTTCCAGATGGATAACTTTAGATATACAGCAAAAAGAGGAAGTATGATAGTATGAATGAAGTAACCAATACTTGCAATGTGAATCAAAGTAACACGGTTCTCAATTCAAACAAGGTAGTATTTCCAGTTCATGAAAATGCAGAATATAGTGCTTCAAGCCTTTCTGGCACTCTTGAAGACGATATTCTTTCACAATTTCAATGCTCAAATGAATACATCAGGCCAAGCTCAATGGGCGAACAATTCAATGACACTAAATACTTACTGGATTTTCCGGAAGTGGTTAACATATTTAATAATACTGATGATCTTTTCCATGAGTCATTATTTAGCATGTCACTATACCCAAAGACGGACATGGAACACATACTACTAAATGTTTAAGACACATAACTAAATTTCCCTTTATACTGATATCGATACCTATTGTCGAAGATGCATTTTATCTATTTATTTAGCACAATTACAAATCTTTAGGTTTTTTAGCTGTTGAAATTAGTATAAAGTAAATCAAAGGAAAGAGCACCTACACCCATATTTTGACCTCGCAGATGAACGACCGACGACACCACTGAAATCTAAAGCACCTCTTATTATTTTGTAACTAATAAATGATAAGTAGTTATCCTTTGGAAGATATTATCAACCTACCGAACACCAGGAAGATCTTGAGTACGTCCACCACGTAATAAAACAACACTGTGTTCCTGCAGATTGTGACCTTCACCTGGGATGTATGCTCTAACGACTCTACCATTTGATAACTTTACACGAGCAACTTTACGAATGGCAGAATTTGGTTTCTTTGGTTTGGTGGTATAAACCTTTGTACAAACGCCCTTCTTTTGTGGATTATTCTCTAATGCAGGCGCTTTAGAAACTTTTGTGAATGATTTACGAGCTCCTCTCATAATTTGGTTTAAAGTAGCAAAGTTGGTAGGAGTTGATGAGATTGACCGCCGAGTGAAAACAGTGGATGGCTGCTGATATTGGGTGCGTAGGTTTAACCTAGTAAAAGATCTTAAGAAAGAATTCATGACCGACATTTCCTTCTATTCAATTTATTGATGGTCGTTTACTTTCAAGAGAGAAGAGCGCTTTAAAAGTGATAACGATGAGCGTAGATTATGATAAAGACGCAGATATCGGGAGAAACGATAGAAATTCTATAAGAAGTTTAAATTCTCTTTGGAAGAGAGTGCCGATAGACGCAGCCTCTGAGAACGTCGTTGATCTCATCACTCAGATTCAATACTGTAAACGGTATGACCCAAATGGTGGCTGGGAGCATGTTTGTGTTCTACTATTTTAAATTTGCAGCTCACAATTATCTAGCTAGAAACTACAGCAGATAATAATATGAGATTTCTCGTAGATCTCGAAAATCGCCTTCAAATGAAGCAAGAGACTGCTAATGAAATAGTAAATGAGATAGAAAATGACGCAAAGATTGTGAGCAGCCTATAGTTGCGCTTTACCTACAACTTATTGCTTATCAAGGTCAAACCAGCGAATGTCTTCCAACAGAAGTTTGATAGTCGTCAGGCGCAATACGATAATAAAACTGCGCGTCAAAAGTATTCAAATAACAGCAAAATTCAATCTTATTACAATGCTATTTGGGTATGTCTCAAGCGCACGCGTCTTGTAAATTGTCTAACAAAGCAAAGGCTGTATCCAAACCTGACGAGGTTGTACCAAATATTAAGAAACTGATACCTAGGGAGGACGATGATTCAGATGACGATGACTCTGATATCGAGATAGGTAGTATGCGTCAAAATTTCAATGACCCTATCACGCTCGGTCTTTTCCAGAATCCATATACTTCGTAAGTCAACTTGAATGATAAGCAAACCCATTAAATATACTTTAGCACCAAATGTAATCACTCCTACTCATTTGAATCAATTAAAGCCTATCTAAATGGTTCAACAAAGCTATGCCCCTATAGTGGTTGTAGAAGTAGCATTGCAATGCGTGATCTGAAAGAAGATCATAGATTAGCTAAGAGGTGTGTCTTCTACTTGTTTTCCCTTTCGGTCGCTTAATTCTCTGTCACAAGAGTACAGCAAATCGCTAGTAGAGATGCTGAAGAGGAACATTACCATCAAATTGACGCTGAAGACAATTAAATAAATATTCGAACTGTATTGATATTGTACAACTAATTAAATTATGCTTCTCTCAAGAATAAATATGTAGTACTCTCATCTTCAAGGCTATGTAATTGTATGCCTTCATGAACGCTTTGAGATCTTCCTTCAACAAACCCATTCCTCAATAAGCCATTAAGTGCATCGCTAATTTCAAGCCTATCATAAGCTTTTTCGAACCGCTTTCTTAGCTGTACCTACATGGCGAGTTAGTTTGATAGCCATCTTTGGGATATGACTTACCATCGAAATACCAGGGTAGATCATCAGGATACCGATTATTGCGAATAACACGTTATTCCATATTTCTATCACAGTATTTCCTTTTAAGTCAATCCATTGACGCGGATGAACCAATTTGTCACCTATAGCTAGACACCAATTCTTTTCGAACTTTGACTTTACAAGCCTAGCTGCATCATAACCTGTAATAAAAGCCGAAGACGTGTTCAACATTTTTTTCACTTCTTTGTCTGTCAATTTTTCACCTTTCAGTAAACCAATCGGACCACATTGATCCAATAAAATGTCATCACACTCATCAGTTTCCATAGACGGTGTGTATACGTATTGCGGTACTGATGGTAACTGAATACCTGCTGCATTGCTGGGAGTGAATGTGATAATATTTTCATAGTCATCATCCTCTGTAATTAATTTCGTAAGTCACAGCAATATTAGGTTATTATTTTACTTACGAACGTCACGACTACCAATACTATCAATTCTGGCACTAAAGTTTTCAATATTGATGCTGCATTTGATTTGATTTGAGGATATACAACTTAAAAGCGCTGCTTGAAGCGACTCCTGAACAACTATTGGCCAATTATTAACATCACCTTTTTGAACACTCATTATATCATCAAAGAAGTCTTGCATAAACTCTGCTTGCCAAGGTCCGTGACTATCATGTTTGTATATATCAGAGAAAGCATATAAAGCCGTTGGACCACTTTTATTTGCAGGTAAATAAAGGATATTTTCATCTTTAATTCTCTTAAATGAAATTTGAAAGGCTTCATCAGGTATCTTCTTAAGAAATTCTTGAGACCTTTCCATGTGTCCTTCACTTCTATCATGTGTTGACATCTTCAAATGTGTTAGTAAAATATTGATTAATTGCATAAAACTCACTTTAATAGCAACTTCACTGAGTCTAATATTTAATTCATCAACTGTATTCACCGGTCTTGAAATACAGTTCTCATATGAATCGAAGAGTACCATTCTCGAGTAAGCATGTTCTCTTGCAATCTCAGTTTGAGCCGTGTCAAATAATACTGACTGTAAATCTTGATATTGCTGAGCGTGATGAGTTTCTTCAGCTATATCGTATTGATCAAAGAAGCTCTGTACATCATCAGGTACTTCCAAAATGCTCGAGCCTTTTCCTGTGGAGGTTGTATTAATATATCTGCCAATCATTAGTTAAAACATTCAACTAAACCTCGAGCACTTACAATGCTCTTTTATCAATATAGATTCTGATTAGCTTTAAATGCTCAATAAAATCAATTTTATCAAGAGACAATGACCTGTTCTTCTCCATTTTCTTTTCTTTGACAAGATCTTTGCATAAACTCTCATATTCGTTTGCCAAACGCTCTATATAGACATGTTCTTCACGATTCTCTAGTAACGTCTTCTCATGATTTCTCAACTGACCTGTCTTAAATCTCGGGAAAACGTGTAAAGCTGCAGACAAAGATGGATTCTTGTGATTCCTAGTACGAGCTTTTATGATAGCAATTGAATCACGCAATAATTCATCAGTCTCAGGGTTCCAATAACTGTAATTCTTCGTTTGACGTGCACTTGTACCTTCAGGGACTTAAACAGTTTAATAAGTATATCATCTGTACGTAAATTAAAGTACTTACAAAGTCTCTTATGTTCTTCTATAAGCTCCTCAGGTGATTTACCAGGTGGTTCAGAAAGAGGTGGATCAAGCCTATAGGGGTTCTCTCTTGTTGGTTTACTTTTCTTTTTTTTCTCGTTTTTCTTTTTAAACTCGCGTATTTGCTCAACTATTGCTGCATCTTCAGCATCCTCAAAGCTATCTTTTTCAGATTCTTCGCTTCTCATGTGTCGAGAACGCGCTTTTTTTACTTTCGCATCAATGGACCTGCTATACTTCGAAGACTTTTTAAGCTTTTTCGTTGTGTTTTTGTTCTTCTTAGGTCTATGAAGAGTTACTCCCAATTTAAGTTTTTCTTGTTTGGAAGTTTCCTCTTCTATATAACTTTTTACAAGTGGTAAAGGTGCTCCGCAGGTATAGGCAATCTCCCTGTATTCCTTCTCCGTACCTGGGCGTGGTATGTCACTTCTCTTCCCAGTATAATTAGCAAGGAGGCGTTTTTGCATTGCTGATAACATCATGTCATTTTGCCAACTTTGATGCTTTTGAATATACGATGCAAAATGTGAGAAGTTCGTCATATCCGGATTCTTCGATTTCTCATTTGCTGTCATTGAAGCATCTTGACTGCTCAACGCAAGCACTTTCAACCTTTCAAAATACGTATCTATATCTTCGTTTGTGTTGAGCGTCATACCATCGATAAGTACGGGTTTATCTTCAAAAGATAAAAAGAGACCATCTTTCTTCAATTTAAAATAAAGGAGCCTGTTTGGTGTCTTTAACTCCCTTACACCCTTGAGATTACTAACATCAGTTACTATTTCATCAGTTACTTCGATCTCCTCCATGAGCTCGACAAGGTTCAAATGCCATAAGATACGGACACATTCAGTAATAAAGTGACGTCTCTTTGAAAATGATAGACTGACAAGTTCTTGTAAATGCTCTGGAAGGTCTTTAATCCTCAGTTTACTCTTTAAAGCATCATTCTTGTATTCAAACGCCATCTCATCGAATGTTGTCCAAGGAATTAGTTTCATGTACATATTCAATGGTATGTCACGTTTGAAGTAGTTCATACATACGATAGGCTCATCGTCGCTCATTTTACTAGATAGCTGATCCTTCTGCTGCATTTCTTGCGATAAAAGGGAGTACAAAGTATGTGCTTTTGCAAACCTTCCGGTCATGTAACCATGAAGCCTAGCCATTGCTTTAACATCAGACAAGATCAGGTTTCTGATATTTTCTTTTTGCGATGGATCAGTGCAATTTTCAGGTAAATTGTCAGCAGCATATTTCCTCCTAAAGTTAATATATCCGATATTTTCGGTGTCTACTTCTTTGAAAGACGCTTTAGGTTGGTATCTCTTCATTAAATCTTGCCCCAACTCTTCTTTCTTTTTGCGAAAAGCCTCCGAATGTGTATCAATATCTGATACGTAAATAAGTCTTTTCTGCATTGAAGCGCCTCCATAAGTTCTACTGGATACCTTTGTTTCTTTCAATTTACCTTCATTAAACAACACATTGAGAGTTTTTCTAACAACTGGCCTATCGATTTGTGCCGCAGTTTCGTGATTGGGGCCATGTACATACTTCTGATACACATCAAACAATTCGCGCACCGCTTCCACCATACCACCAGCACTCTTTATTGCATTGTATAAATCTTGAATTCTACGATGTTGTGAAAGATCTGTTCTTGATGCTCGCATTTGCTGAATAGCTCTCTTCGTTGTCTTGCTTCTAGGGGTTCCATTTGCTAATTTTGATGTTGATGTCATAGTTGATTCCATTTGATTATCTGAAATGGCAGTATCTGCATCCCTCTTAACATCTAGCTCGTTAATTTCGGATGCGTTCTGTTGTTGAGATGTGTCATATTGTAATTTGTTTACTTGATTTTCAACCTCAGTTATGACTTTTGGTTGAGGCTCCTGTATATTACCCTTCTCATCTTCGACACCTTGATTTTCGGTAGTCTCGTCTTCCTTTTCATGATGGTCTTCACCAGGTTCATATGCATCGGACTCATCACTATCTTGTGGACTTTGAACTCTTTTTCGTTTTGTTGATATATGATCTTGACTAACATCTTCGACATCAATTACAATAGGAGCGATCCCACTTTTCCTTGATCTAAGTTTGCGTTTTTTAAGGTATGAATCCTTATCTACATCGTTTGTAACATTGATAGTATTATGTGCGACTTCTTGTTTATTTAGTTGTTGTCTTTTATTAGGCGTTGACATGTGACTATTATCGTCACCGACCATATCAAGTTCAGGATTGTGATGAGCTCTTTGAGGTGCAGTTTGTGATGTTGTTAAAGACACTTCAGATGGGGGCTGTGTTTCTTCTGGCGTAGCATTCAATGGTATGCTTTCAGCTACATTTTCCTGTAATTCAGAATGTTTCTTTGTCACGTCCTTTACAGTTTTTTCATCATTATCATCATCATCATCATCATCATCATCTACTTCTATTGTATTTTTTGAAGCACGACCTTTCTTTTTAGGCTGATCTTCGATGATTTCGGACTTCCGTGGACGGCCTCTCTTCTTTGGTACCTCTGATGGTGTTTGGCTTGCTTCAGACTTACGAGGACGACCTTTAGGCTTTGGTGTAGGCATTGCTGATTTGGATGATTCTGCTTTCGGAGGTCTACCAAGTTTTTTAGGAGTAGCCACCTGCCCACCGATCGATCCTATCATGCGAGTTGCTGACGCCTGGTACTCTTGACGAGACGAACACCCCAAGGCAGAATTAATAACCTTCAATGAACCAGATTGATTGGAAACCAAGTCTTCAACTGGAATATCTAACCCTTCTTTTTCTGCTAAAAGTATATAATTCTTACGAGTAAAAAATCGTTGCCTGCGCTCTCGACCATTCATTTCTGTGGTTGATTTTAGTGCAAGGTCAGTGAGGCTAGCTGGATCATGATCTGTCTCATGACGCAAAAGTATGTTTTCGACAGTTCTCCTATTGATGGTAGGAAGAGCGCTAATAACTGACTAGCAAGATGTTAAACAATACATTCTATTTACCTTCTCAACTTACAGAGATAGTCATCCCTTCAATACCTGATTCATGTATAAGGTTTAATATTTGTTTTTCCACTGTGATAGCAATCCCAGCTCCAAAATTATCTGAAACAACGTCATCATCATCGTCTCCATCATCAGATTCTAATTTAATCATATCTTCCTCAATGACTTTCTCAGACGACTTTGATCCGTTATCATCATCTGCAATACGCAAACAGTTAACACGTGTGTCCCTACCTCCAACTTTGGTAGCACATTTTTCGAGTGAACCCTCTGCAACCATTCTGCCTATAAGACGTGCCACGAGTCTTTGAATAGGTTTACTATTTGTTTTAAATCCTATTTTGTTAAATATATACTTATGTTGAATAATCTTGTTCGGACAGCTATTGATAAGTTTTAAGATACGGCCTTTCAACAAATTGTTGTTGGTGAGATGTTCCTTACTTAATTCTGAGAATTCTGGTAGACCTGAGGCGTCACGAGCTTGTACTGTTTCTTCAAAATCTATTGCTTCATTGTCGGAATCATTTTTTGCAGGTTCTGCTTCTGTGAGTGCATGTGCTCGATAGTGTGGTGAGCTGGCCAAAAATTTACGATGTATAGCAACATTTGTGGTGCTACCTCTGTAGACAGTGTGCATTTTTACGCTTATGATACTGTCAATTGAGGGCTAGATCAGATTGTTAACTTACACAAGATCCAATTCTATAAGTATTTTGACACAATAAAATACAGTTTTGCTGTCATAACCAGTCATCTTGCTGATCTGCATAAAGGTGATACCATTTTCTCTACTTCTAGAGATAAAAGTGAGCACACTCAATACCATCTGGCTAAGTTTTGCTGGTTTATGATTAAGACCAGTTATCACAAACCAATTCATGTCACTATCAGTACGAACGCGAATATCGTTACCGTATCGTTCAAGAAGGGAGGCCAAAGATGTTTGTGTATCATCGAAGTTGAGTAGCTCAAACATGATAGCTTGATTACCGTCTTTAGAAGATATCGGAGGGGCTTTTTCCTTTAAGGCTAGCTTCAACTGTTCTGTTGCTAAGCCAATTTGAAATGAGGGTTGTTGAGTGAGAATTCTCCAGAAGAATTCAAGTAACGGACCATTGTTGATATCTACCGAAGGATCTAACTGTGCTTTGATGTATTTCACAAGAGTTTGTATATCGGAGCCTATGATTTGTTAGCAACCAGAGATTCGAACACCGGCACCTGCTGACGGCGCCTGATAACGAAAGTGACGTACCCCAGTCACCATCTACTGAGATCTCATTTAAAACGTTATGTACTACTACGTCTGCCGCAATCATTATAAGAAGCTTCCTATATAGCTTTACGAGTCGCTAGTACGATTATGCGAGTACTTGTATTTAACGTATATTAATTTTATTTATTTACGAGAGTTATTTCTTCTTTATCTCTCGAATTAATAATTCACTCCTGATGGTTGAAATCAGAGATAGGGCCAAATGCGAGGAAGTGTATGAGAACAAAAATTACATTAAAAAAGGAGATCTTCTAAACCACTATTCTTATTCTGCCCTTGTGTGTTGTTTGTTGTTGTCATATCGAAAATGTCGTTATTTGCGCCGACGGACTGACTAGGACTCGAAACGGTGGCATTTCCGAATAATCCTAATAAGTCATCCACAGGATTAGACTGTGCGGGACTTGGTGCATGGTTAATATTACTTGTCGCCGTATTGGCTGTGAAACTGTTGAAGCTTTCTTGTGGACTTTGTGTCGTGGATGGTGAGGCATCTTCATCAAGGTCTAATAAATTTTCTGCTTTAACACCTACATGAGAGTTTAGAAGAATGCGCAATCACAAATTTCATAGATACTTACCTTGTGCCACCGTGTTCAATGCTTTTTGTTTCGAAATATTCTCATTTTCTTCATTCGGATTAATCTTCTTTATTGACTCAATACCAAGACGACCTTTACCAACGAAAGTAGATGCTGGCTTGTGATATGCGGCTGCAAGGGAAGAAATTTCTTGAACTAGTTCTTCTAGGATTGCCCGCGAGACTGTTAAGTTTGGAATGACGATAGGTGGCCTATCCGCTATAGTAATTGACTATAATCGTTAGTCAGTTCTAGCACTTAATTAGCACTCACTCTTGCTGCTCCAGTATCTGTTGAAAGTAGTCTCCAGTAAATAAATGCACGATCACGTACATCTGAGTTATCACAGTCACTTGTAGCTGTCGTCAAAACCCTTTGAACCAATGACTGAGAAGGTCCCTCTGGCTTCTTTAAAAACAGTTTTACTATCGCACTTAAGGTTTGTAATTGAACCTAACATTATGAGCTTATCGCATGCTTTAATATATTCACTCACTGGGTAAGAGTCCTCGATAAAGCTATCCAGATACGTCTCTAATAACTCCTCGACATTTACTATTTTCTCTGCATTTTCACCTAGGATCCAAATCAATGACGCTTTGGATTCAGGTTCATCAATTTCTTCGAGGTTTTCACAAAGTGTTGGAATAATTGCTTCATATGAGTGTGGGTATTTACGGAATATATCCTAGATAAAGGTCAACCAAAATCCACGTTCGAGAATAGTGTTTCTCACCTTTATGACTATGACTGCTTCTTGTACAACGTATGATACTTTTGTATTGATCAAGTCGACGAGAACTTGTACACAACGCTCTGCAGCATCTTCAATTTTGATTGCGCACCGTCCTATAGCTCTTATCGATCTGCGAACGAAATCAACATCAACTTCACTAGCATATTCCTTAAGCTCGTTTAATAGAGTCTCGACATTGTTCTCATTAGCTAATTTAACCAATATGTCTAATTTTTCAACTTTGTTGTATTGTGCATCGGAGTACTTGCAGAAAAAGACGCGCAATTCTGAAGAAAGAACGTCTGGACGAGCTTGTAATATCAAATTTATGTTTCTAAGAGCTACCCATTGTACCTCAGGCTCTGATGCCACAAGAGTGACTGAAAAATTGTCAAATTACCAAGTAGTAAATGCATTGTACTTACCTAGAGGGGGTGCCATCTTACGAACGAGCTGTCTTATAAAGTCTTCCCTTTGTAAACGTTGTAAATTAATCATAACGACTTTAATAGCACTCAAAACAACACTACCATTGGCATGTTGAAATTGTGGTAAAACACGTTCGCAAATGTATTCAGTTTCCTTCTCCTCTGTTGGTGTATAGCGACACAACGCGCTCAATATTGATATTCTGCCCCATTCGGTGCATTCATTAAGAGCGACTAGGATCTTGTTAAGTATGTCCCTATCAATTATGAAACCTGGTGAATCTGGATGAGGGGAATTATGAATATCAGTCAAGGCAGTGACGGCATTTGCAACAACCTGGGGAATAATGAGATGATTGAATAATACGACAGTAATTTTCTTACAGTCGGATTTGGATCACCAACAAGTTCTTGAAGCGTTTCAACAAATCCTCGATCAATCGCAAGTGAAGGTTTAAGATCGTACATTTTGGCAACGCATAGAGCAGCTGTTTTGCGTACATATGGGTTATCATCTTGTAGACCTGCAGCAAGTGGATCTGAGAGATAGTCGATAATCTTTTCTGCTCTGAGACATCCCATAGTTCTTATAGATAATGCACGGACTAATGGATTAGGATCGGAAGCATCCTAAGTGTTGTTAAGCGATAAATCACAGCCAATTGTCTCACCAACCTTGACAAATGTGTTCACGGCAAGTATAACGAGCTCTGGCTGTGTTTTTGCGTAATTCATAAGATAAAGATAAACGAGCTTCTTCTGCTCCAGATCTTCTGTCTGCATGTTCTTCAGTACGTCTGGAAATAAACCCGATACGTCTTTTCCAACAGTCTGAGAGATATCAGAGATGCACGCACTATAAGAAATAACCAACCATATTCGCGATAACACGTTTAATAGCATCCTTTCTTTTGTCGCGGTATTCACTATTTAAGTCTGGCTACGTTAACTGCAGGTAGTGCGGAGATATCGTGATAGCATACCTGCACGGAGCTCATAGTTCTCTCCTAAATAATAGATCAGAGCTGCTTACTTGGGATAGCTGTTCTACCCACCTTTCTTCGCTGTGAAGACGTTTCTTCTTATCGTATTCCTGCCGTTGGGTTAGGAAAGCTGGGAAAGGAGATATGGCCACCTACATTTTGTAGCTAAGTAGTTTTTTTATTGCAACGCCAGTTCAGGCGAATAAATTGATTTCTTACTGCGTTGCTGTGATAGAAAGAAGATCGTAGCTCTCCTCTTTAATTTTGGCCGTCATCTAAAATATGCCAAAAGCATTGAAATCAGCTACAGAGAAGTTCTCCAGGAGCCATCAGCCTGCTGCTAAATCAGATAGCAATTCAGGCGGTACCAGCAATCCACTTTTGTAGGCAATTCCGTTATCAGCATCTACAATCACTCATTTAGGACAGTAACACAGACAGATTCGGTCAGCATATTCTCAAGAATCCTCTAGTAGCTCAAGCGTGGGTATCATTAGATTATTACTGCACTTCTGAACTCTCAAAAGTATCGTTGATAAAGCTTCTTTGCGAGCAACAGACAAAGTATTCGAAATCGGTCCAGGTACTGGAAATTTGACTGCTAGAATACTTGAAAAAGCAAAGTCTTGTCAAGTAATAGAAATGGATCCTAGAATGGCTGCCGAACTCAGCAAAAGATTTCAGGGAAAGTGAGTATCAATGGAAATCATCAATACTTGCTAAATATATTAGACCTGAGCAGCGTAGATTAGAAATCGTCATCGGTGATTTTGTCAAGGCAGATTTGCCTTTCTTTGACGTAGTGTGAGTTCAGAACTCTAAAAAGCACACATGTCTGACATAAGCAGAATATCCAATTGCCCATACTCAATCTCTTCACATATCGTGTTTCGTATTCTTTCCCATAGACCTATCTGTAGGGTCGCGATACTGATGTTTCAGCGTGAATTCGCCTTGCGATTATGCGCAAGACCTGGTACTGAGATGTGGTGTCGCCTCAGTGCCAACGCCCAGTTATATTCAAAAATCGATCATATTATGAAAGTCAGCAAGGGCAACTTCAGACCACCGCCACAAGTCGAAAGTAGCGTTGTAAGAATTGTCCCCTTAGATCCCCCACCGCCAGTCAAGTTTGAAGAATTCGACGGGTACGTCTTATTTTTTTCACTTTAGCTATTTAACTGTTTCATAGTCTCACCAGAGTGCTTTTCACAAGAAGGAACAAGACATGCAGATCAAATTTTACCGCTGCTGGAGTTTATGATATGCTAGAAAGCAATAGGAAAGCTTGGTTGACTGAGAAAAATGAAGTATGTTGGATTATTACCAGAGATATTAATTAATTGACTGGTATGTAGATGATAGACGACTCAGAGAACATTAAGAGCAGAGTCGAAAGGATATTAGTACAAACTGGCTTTGCAGAAAGCCGTGCCGCGAAAATGGACGTTGATGATTTCCTTAAGTAAGTGCGATCCGTTCCTCCTATACATGCTAACTTTGGGATTTCAGGTTACTTAGTGCATTCCACGATGAGAACATTCACTTTGGTTAATTAATTTATAAGTGATTAGATATATATTTAATTTTATTCATACACTACAATCCTATTCGATTTTGTTGAGAAAACCAAAACATTTGAAAATTTTACTCGTCAATGACCTTTCTAGCGCGACTTCTAGTCCTATAAGCAGGTGAGTTATTTGGAGAAGACTCCACTGGCTCTACTGGGGCTTTCACTGGAGCTGCTTTTGGTGTAGCTTCTTCTGTCTTCTTGTCCTTAGCTGAGTAGATCATAGCAGTGAATGGACTGGATTTTGTGAGTATAGAATTATTATAAGAAGCACCACTCACCCTTCATATCTGAGAGCTATTTGGTATACGATGAAGACGAATACACTGATTGCGATACCAGCAGGTGATCTGTACCAAATGGAAGTACCGAAGAAACAAAGTGTACTGCCAATGTACATTGGATCCTCAACAACATTGAATGGGAAACTTGTAACGCGGTGGCTCATAAGTATACCAAAGTAGTCGCCCAAGTAGGTTCCAGTAACGCCTAACGCAAGCATAGAAGTTATAACTAAGATTTGGCCAGAGAGGAACAAAGCGAGAGCAACGTATACATGCGCAGTTGGGTAGTATGGATGTGGAGCCAAGTTTGGTTGTTCAGCTAAAGCACTCTGAAACCTAGAAAGATGAGAAAGAAGTTCTACTATAATACTGCACGTACAGATGATCACGCAAGATACCTAATGAGAAAATGGTAACAGCAAGGAGGTAACAGCCAAGATATGGTCTACCACCAGCGAGCTTTGTGAGTAGCTTATTGTGATATTCTTTTTCAACGTGTAAGTTCAAAACTCATGTCTAACGAGATAGTACAAACCTTGACGAGCAACCTATGAAAAGAATCAGAATCAAAGGCTTAATAGTGTCTCTATACTCACGATATTCCAGAATAAGGGGTTGAATACAATTGATGCGATTACGATCCAAAGTGAAGGTTGCTTGAAATCGAACCAATCTTCTGGCTTGTCCTGTGAAACAGATAACAACGGTGAAGGGTGTGGAACCAGCTCAGAAAGCCCTCTAATAAACGGATGTTGCTTAGCGACCATCGACGTAACGTGTAAAGCGAGAGTTTTGTGCAAACCACGAGGACGAAATACTTTATAAAAACATAAGTATCCTTAGAGTCTTTTATTAATGACAGTCGACCCTCAACAATGGCTCGACACTATCTTATCGGGAAAGTATCTTGCTGAATCCCAAATCAAGATGTTATGCGATGTAGTACTCGGATTACTGGTAGAGGAATCAAATATACAACCCGTATCAAGCCCTGTCACCGTTTGCGGAGATATACACGGTCAGTTTTGGGATTTACTGGAGCTTCTGCGGGTCGGTGGTGATTGCAGGGATACCAACTACATTTTCATGGTTAGGAAACAAACTTATTAATAAATATGATGCTGATTATGGGTAGGGTGACTTTGTTGATAGGGGTCATCAAAGTTTAGAGAGTTTTACCCTTCTTATGGCCCTCAAAGCCAGGTTGGTTGACATAATTCTATTATGGTTCATCTAACAGCTATAAGGTATCCTGATAGGATAACTCTACTGCGTGGTAATCATGAATCTAGGCAGATCACTCAAGTATACGGATTTTATGGTAAGCTGGCTTCATAGTCGAGCTATAGTATTGACCGAGAGTCTAGACGAATGTCAACAAAAGTATGGCAATGCAAGTGTATGGAAAGCATGCTGTCAAGTCTTCGACTATCTCAATTTAGCTGCTGTAAGTCTAATGCCATTCAATTAGTCACAGGCTGAATGGCCAAAGATCATTGATGGTAAAATCTTATGTGTTCATGGAGGTTTGTCACCAGACGTACGAACTCTAGATCAAATTCGCGTAATTGGTGAGCTTCAGTTGGACCACTCAATTTAAAACTTATACACCTTAAGCACGAGCTCAAGAGATACCTCATGATGGACCGTTTTGTGGTAAGAAAGGTATTTGTTGTGGTTTTCTCACTAATGCGACCATTTAGACCTCATGTGGTCGGATCCTGACGATGTAGAACATTGGAGCATAAGTCCCAGAGGGGCTGGTTGGCTATTTGGTAGCAAAGTTACAAAAGAAGTAAGATTGGCTTTAATTCTATGATCATATTTCAACTATATGCAGTTTAATCACATAAATGGTTTAGACATGATCGCCCGTGCTCATCAGCTTATTCAAGAGGGCTTGTATGTATCAAGCATCGTAGCTGATATTTTACTTACTCGCCTTAGCAAATTCATGTTTCCGGACAATGACCTTGTGACTGTCTGGTCAGCACCTAATTACTGTTATAGATGCGGTTAGTTGGAACGCTGAAATTGGTCCGCAGTCATACTAAATAATGACCAGGAAACGTTGCCAGCGTTCTTAAAGTGAAAGAGAATGAACCTGTCACAGAAAACAATTTTGCAATTTTTGATGCAGGTACGTATTTCTATGAAGAAATAATACGGCTACTTACAACTACTATAGTAGCAGATCAGGACAGAGGTGCTGTTGCGAGAGTCGCTCCTTCTCAATACTTGTAAGTACGCATGACCTTCTTGTATCTCACTGATATGGCCAGCTTGTAAATTCTGTTGTAAATTATGCGATAACTATTCATGTTTATTCTAATAACATAATTGTTTATTCTAGAACGTGTGAATGAGGCTTCGGCCAATGGGCGATTGAAATGTTGAAAGATGACGTCACTAACATGCATAAAGACAAAGACAAGATAAAAAGACTACGAGATTCCAAGTTTTCAAATTCGTCCTTGAAAACTGGAATGATCCCTTTCCACTCATGGCAGATCGTTCCGCCATCGATGTTCACCAAAGCTTTCACAAGGCTCGGAGTCTTATATGTATCTAGCTTAGCCGAATGTAGTCGTAAATATATAGGTATAGTGTTATAGACCATATCACTAGCTTTTGCAAAGTTAGCACTATTTGGACGTTTCGTCGACACCTGAGTTGCTCAGGTGTAGGCTCATTCACTAATAAAGCACGAACTCAAGTTAATGAGGAGTACCCTCTATCCAGTCTCAAGTCAATATGTTTCCACGAGGACACATAAAGGAGCCCGTGAGAAGGAACATCAAATGCAATAATCATCAACTATCGCATCCGATTCATGGAGAAAGCTATGCGAACAAGCTCATGAAAACTTGGTAACCCCAAAAAGTAGAGAGTTACATCTAATAGAAGGAACTAGCTTACTGACGACGAAAAAGAGGTGAATGATTGAATGTCCAAAGTATTAAGCTAACATTTTCAATCGCATCGGCAAGCTTATCTCAGAGAGAAAATAAAAGAGGCCTCTGAAGAAGTAAACCAATGATCTTGCTCTATAGTGATCATCAAATTATTGAGAATTATATGTACTTTGCTGTTTTGTGATATCATTGAACTTTAATTGTTCTTGTTATATTTCATGTATATCTATTAATATTTATATTTATATTAATTCATCCAAAAAATCTTGATCAGACCTTGTGTCTATGGTGCTTATATTCATGGGCTGATTATCGCGTTCGATAGCGACTGTTTCACTTTTAACGCGATGCGCTGTTTTTGTTGGTGAGCTACTTTTTGGTTCGCCCACACTTTCAAAGAAATCGTTGTCTTCTAAATGATTCACGTCTCTCCTTGATGATTCGTATCGCTGGATGTATTGTCTACCTGAATCACCTCTCAGACGAGATTTAAGATCTTTAAGGATGTCAAAAGATGTAAAAGATTGCTGTTACTCACTCCATGATGCTTCTGCCTTCATCCTATCGTTACGCATCTCCTCCAATGCTTTTGAACTCTCTGAATCAATAGGAAGTAATTGGGGTTCTTTCTTCTTGTGTCTATTAGCCAAATCATAGTCTATATCATCATCGTCTGCATCTGTAGGTCGCTTTATATATTGTTCAAAATCGTCATCTACCTTAAGTTTCATTGCGATGGACGAGAGATCACGCTCTTCTTCTGATGAAACTGTGTATAGTCAGTCTAGTGGATCTATATATTCCCTCTCATACCTTCCTCTTTCTCGGTCGCGGGTTCATCGTATTTACTGGAGTTATCAACTAATTTCCGCCTAGAGTATTTTGGGTTCGTTGAACGCCTTGTGGCTAATAAATTAAGCTTTATCTAAATGTATCCCCTGTAAACTCACTGTTATTACTAGCAGATGGAACTGCAAGCTTATCTTCACTTTGACTAATCTCTGAGCTATCATTTTCGCTACTACTGCCATCTGAGCTATCAGATTTCTTTAAGTCAGGAAATTCCGCTTGATTATCTACTTCTGGTGGCTGTAGACGTCGTGTGGTGGGTTTTGGAGCGGTACTTAAACCTCTTGAAGCTTTCCATGCTTTTGAAGCTTTAGCTTTATATTTATCTTGTAAAACCATTCAAATTTCTTATTTTTTGACGAATTCGTCGACTTGGTTAGCAACAGTCAAGAAATGCAAACTCAGATCCGTGAAGTCTGGGCAGATAATCTAGACGCAGAGCTAGATATTTTGCGTGAATTAGTCGATCGATATCCTTACGTTTCTATGGTGAATTCAATCGACGTATTTGACATCCAAACTGACGGTTTTAGGATACTGAATTCCCTGGCGTTGTAGCTAGACCAATTGGAAATTTTAAGAATTCATCAGATTATCACTATCAAACGCTGCGATGCAACGTAGATTTATTAAAGATAATACAATTAGGTATCACTATCGCTGACGCAGACGGCAATATGCCTGAGTATCCTACTTGGCAGTTCAACTTTAAATTCAGTTTAAAGTACGCCTCTTTTATCATATCATTCATCATTCTAATATCTACATAGCGATGATATGTATGCACCAGATTCAATAGATTTACTCACAAAATCTGGTATTAATTTTAAAAGGCTAGAAGCTAATGGTATTGATATTGAAGAATTTGGAGAGAAACTGGTCACTTCTGGTTTTGTTCTGTTCGATCACGTTCACTGGATATCGTTTCACAGGTGAGTAACAAGCGCGATGTAACTCGTTTACAAACTCAATATAGTGGCTATGACTTTGGTTACTTACTCAAAGTTCTCACTTCATTGCCCTTACCTTCAAATGAAAACGACTTCTTTGATCTACTACGCATTTGGTTCCCTTGCATTTTTGACATAAAATACGTTATGAAAATCAATAGGCTGTTAAAGGGTGGTTTACAAGATATTGCAGATGAATTACAGGTAATAATAAGCATGTTCCATTATATCAGCTGCTCTGACTCTCATTAGGTGATGCGAATAGGCCCTCAACATCAAGCAGGTTCAGACTCCTTAGTCACATCAGCTTCCTTCTTCTCCATGCGTACAAAATTCTTCGATGGTACATCTGAAAGAGATAGACAATTGTAAGACATCCTTTCCGAAACTTATTACTTTGCTAATTTCACTATCAGACCTCATTGTATGGGATATCTTTATGGTATAGGTATGGGCGCTGGAGCAACTGCTATACAAACCCCAGGGGCACCTTTCACACCAATGAGCAATCCAATCCCAACTCCTTACGTTGGTCCTCAACCCATAGCACCTTATCCAAATCGAACAAACGTCGGATTCGGAGAAAGGTAAATTAAATTGTATTTTGTACATTATCATAAATCATTTCTATTTCCAGAATTAACAGAAAATTTTATGAATTCGAAATTCATGCAACATAGTGATCGAGGCCGAATAAAGTTCCCATTGTATACAAAATACAATTAAAATAAATCCACAAGCTACTCCTTAGATTCCTTGAACATCTCATATTAATCTAATCGAAAATGTTCCCTTCTTGGCTTTTTGAGCTGCTGTCTATACTGGCTATTGCGTCAGTGTTGATACCAGCACCATTCCATATTAAAGCACAGAATTATGCCGCTGTCGCCCTATTAAGTTGGCTGTTGCTTGACAATATTTTCAAAACAGTCAATAGCTTTGTTTGGAAAGGTAACGTAGACATCAAAATTCCAGTCTGGTGTGACATTGGTAAGTGAAGAATCGATAATCAACCTCTCGTTTTAACAATTTAGGAATTAAAATCAATATTGGAGGTTCGATAGCCTTACCTGCTGCGTGCCTCTGTATCAATCGCTTTCTTGCAAGCATCTCGTCTTCCGATACAATTAGAGCTACAAAGAAAGAGAGGAGAAACCGTCTATTGTTTGACCTTTCAATGTGTTACGGCCTACCGGTACTATTCATGATACTTCATGTTGTTTTTCAAGGCCATCGCTTCAACATTATTGAAAATATCGGTTGTTCTCAATATACATACAACAATTGGGGAAGTATTATTGTATTCAATATCTTACCATTAGTCCTAGCTGTTTTATCAGTGTTCTATGGATGTAAGTCTAGCTATAGTCTTTAGCTGACGTGCTGACAACATACAGCCTATGCCAGTTTTCACTTGCTTAAACGAAGATACCAATGTGATGATGAATTTTTAAGGGATTCGACGAATTTCACCCCCAGTAGATTCTATAGACTACTAGGGTTGACGTCAACCTTAATACTAATCAACCTTGCTTTCAACATAATGGGTATGATATTCAGCGTCACGTCTTACATAGGTTCTTATGAGAGCTGGGGTTACGTCCACAAAAATTTCTCACATATTAGCCAATTAGGTCTTGAGGTATTATCGCAAAAGGCAATCATACGAATCCAAATTATCTGGTTACTATCTCCAGTTGCAAGTTATATATTCTTTGCGTTCTTTGGATTTGGATTTGAAGCGAGGAAAGAATATCTATCATGGTATCAAGGCGTCAAGATGTTAATAATCTGGTGTTATGAAAAATTTACGAAGAAATCTGTCTCTAAAACATTCAATCCACCAACCTATGAAGATTCCAGGTATTCAAATGGAATTGATTATTCCCTCAAAGATATTACTGGTAAAACAGGAGTAGACTTTGATCAATATGAGCTTCCCTTCCTAACTAACTCACACGACTCCCATACAGTCAAGGTATGAAAAGGAGAGGTTCAAAATAGACTTTGTAATTTAGATTATTAATGTAAAAGCCATGTATTAAATTTATACTTGATCTTCATCGCGACTGAGGATGTGTAAGAGAAGAGTTAGGATAGACAGTAAAGACGTACCCAGCGTAGAATTTTTCAAGGAAAGTTTGTGGTCTAGCTGTGAGTATTATGTTAGTTTTGAATAAATAATAAAGTTATCAAACTGACGTGGTGCCTGTGTAAAGTTAGATATAGCGAATGAGAGTAGAAAGATTACTCACTTCTTCCAATGCAAACCAGCGCATAATGTGAGTTTCTTGCTTCCATGCGTTCATTGTTTCTTGGTATTCCTGTCTACCTTTGAATAAAACTAAGTCTATCACATTCAAATCGTCGAGATTTCTGTTCTTTTCAAACTCCTGACGAATTTTTGCTCTAATCTGTGATGGTGGAATATTTAATGAATAAAGCGCACAGATACTTGGTGCCTATAAGTATATCTTAATAAAGCAATCAACCATCATCTATTCTATCACTCACGCTACGATAAAAGTCACGATAAAGAGTTCTAGCTCTAGCGTGAGCTTCTGCGAATGAAGTTGACGATTTTGTTACGTGTGCCAATCTTGAAGGAATAGTAGTCATCGTCGGGAATCAAGAGATAATAAGAAGCTAGAACTAATAGATAAAATCTGTAATAGTATAGTTTATTCAATTAATCGTTCATATTTATTTATTCATACGATATATCTCTTCATCTTCGTCCTCGATGTCCGCTAAGATACCCAGTACAGCGTTATACGCAAATCAAATTGAAAGCCCACCAAGGCTCAATGGTAGTCTAACGAACTCTGCCTTTTCTATATCTCTTATTGCTTTTACCTTAGGTGCTGTCTTTGGTTTGAGCTTACTTCCCGGTTTATCAGTTATCAACTTAGATTTGAGTGCTATAATTCCAACAGCATTACATTCAACCCAAGTTTCCCTATATTTAGTCATACTTTGTTGGTTTCATCTTTGGGAATTCCTCAGTACAGCAGGTTGGAATATGACAAAGACATCAGTCGATTGTTAGTCATATACTTAATTGAATATGCAATCTAATAAACTAACAGCTTACTTATTAAACAATGGATATAGCTACATCGCTGCACACGCTTTCGGTCTAGTGTCTCATTACCTTTTGATATTGTTCTACCCTTCAATTTCTCAAAATCATTACTTAATCATCTCAGGTAAGTGTGGTATGAACCATTTAATTGCCCTTACCATAACTTAAGGTCTCGCCTTGGTTGTCGTAGGTCAGGTCTTGCGTACGCTTGCTATGGTACACGCGAGTGAAAGCTTTTCACATCATGTCGCATCCTTTAAACAAAAAGATCATAAGTTAGTGACTAATGGCGTTTACAGGTAAGAGTATTTTGCTATATAATATTTTTCATTAAACTTTCACAGAATCGTACGACACCCTTCGTATTTAGGTTTCTACCTATGGGCATTGGGAACACAGATGATTTTGACAAATCCTTTATCATTCATAGCATTTGCTATTATTCTGCATAGATTTTTCACTGATAGAATTAAACGTAAGTTTTTCTCTTTTCCTCACTTAAGACATTCTTCTAATTCTCGCAGATGAAGAAACTTATCTCGTAAAGTTCTTCGGAAATGACTATCAATCTTATAAAAATAATGTGTACTCCTTTTTCCCGTATATCATATGAATATTCTTCGAATGGTATCTTTATCAAACTTGGTAATATGAATGGTACGGGCACAAGTACTAAGAAGCTAAT

GACCGCTAAGTGAATGGAACAACGATGGGTACAACAAAAAAAGAAAAGAGAATAAGTAATTGCGAAGAGCAGAGGAAATGGGTGCTGTAGGGAGATATTACAAAGCTCTGCTGTGCGACTGAAGTGAATGGTTAGTGGAAATTTGAAATCCAGATATTAACATACTTAGAAGATTAGTTGTTATCCACTTTTGTGAGTCATATCATAGAAATAGAGATGATGAATGCAGTGTTATATATATTTTCTATTGATAAGATGAAGTTTGATTGATGTGTTTTCTTTGTTCATATGCTTCCATATCGTGCGTGTAATTTGTTATATATAAAAAAAGAATCCTTTTTTTTGTGAGTAAAACTCATTTAGTTGATTGTGATGAATTATTTCTTTGATGTTGTTTATCTTTGTTTGACTTCTTTTTAGAATTATTCGACTTATTTTTACCTGTTTCGCTACCAACACTAGCAGACCTACGTTTTTCTTTATTATTCTGTTGTTGCTGTTGTTGCTTCTTCCGTGATGCTTGTAAACTCGCTGAGTAGAGGTTATTCAGGCTTGAAGAAGTCTTGAATTGTCCTTGTGAATTGTTATTACTCAATGCGGGAAAACCTTCTTCTAATCCAGTCGTCAAATTTGGTTTAAACGGTGGTGTATCCTCATCGACATTGGTTGCATCACCAGCTTTATCATCTAACAGAGGTAAAGTTGCGTTTGGAATTATTCTTGAATACGTTCGGCCACCACCTACTGACTTCTCAAACTCTCTACTTGATATTCTAGACGTTTTGTTGGATACTCTCGGAGTTGAAGGTACTGAACGTGATCGAGTAGGTAATTTACGACCCGTTGTAGTTGATGGAGTTGTTGCCGTTGTTGTTGTTGCTTTTGAAGGCGTTTGTGTATTCCTGGCAGCAGCTTGTTTCTGCAATTTAAGCCAACGTCTTGCTGACATCCTTGGTGGTGGTACACGTACATCAGCTGCCAGAGTTAATGCAGACGTAGTTAGACTTTCCGAAGCGACGAAAGTGTTCTGACCGAATGTATAACGTTGTGCTTGTGGGAATGGAGGGAATCCCAATCCTAGTCCTACTGTTTGCTCTTTCTCTTCTCCATTTCTAGAAGGTTGAGCGGTTGATGTTACAGGCGCACTCAATGTAGATGTTGTCGACAAAGGTGGTGAATTTGTAGGTGTACCAGACTCAGACGGTTGAGTGAATGTTGACGGTGTTATTCTAATTTGCTGACCAGCTCCATTTGTAACAAACGTCGGCACCCGTATTTTCATATCACTGTTATTAGCTGCTATTTGTTTTTGGATCAATTGTGAAGGTGAAGCATTCACACTATTAGCGGTTGTTGCTGCTGCAGCTACCGCTGCTGCGCTGCTCGATGCACGTGAATGTAAAGCAGCATTGGCTAATCTCGGAGAATGCCTCAATGGTGCAGGTATTGGTACTGGTGGCCATCCAATTTCAGCTTGAGTTGTTGCATCAGCGTAGGGCCCTGAAACATTAGGAATTGTAGCTTTGAGATGATGTTTTGGTGCATACTTCCTCTCATGTAATCTAGATAACCAAGTTGGTCCCACGTTTGCACCTGAACTAGCTCTTCTTGGTATACTACTACTTTGTTCAGTTCTTGTGGATTCATCTGAAACTAAATTTTCTGCTTCCCAGAAAGCACTACCTAATGGTTTAGTATGTGACTGAGGATGTTGATACATCAGATGCTCTTCATGTGCGTTCCTCATCCTTTCATTTTGAGCAACATTTAATGCTATTGATAACGCTTGAGCATGTGATGATGATTGTCTACGTCTATTAAATGTAGCTGGTGGTGATGATATTTGATCGCTGACTTTTCTTGAAGGGAAGATTGAGGATTGCGATTGAGTAGGTGAATATGATTGTTGAGATGCAGTTTGAGTCATATGCGTATTGTGAGAATGCGTTTGTACAATTGGAGGTGGTTGTAAGCTAGGTGGAGGATGTAATGAAGGTCCTAGCGTTGGTCCTAAAGATGGGTTCGTGAGAGGTCTTGGTGTGCGATTTCCAGCGGAGAAAGGAGCAGGCACGAGGGCTTCTTCTCTTTCCTCACATAGATCTACGAGTACTTGTGAAGGTAGAATATCGGTTTTTCCTACAGTTTGTAACATCCTTGAAGCACGCATGAACTCGCCACGGATTGTGTATAAACCTGTGGTTGTTAGGAACGTTTCATTCAAAATTTTCTAAAACCCACCATCAGCTGTTACCGTTCTAGCTACGTTATAGGTAGTATCAAATGGATCCTCTATGCAAAGTCTATTCCTATCACGAGCGAATTCACTGGCACCATCGACTTCTAAATCTTGCACCCAACCTTTAGCGTCCTTAGAGAGAATACCACCTTCAGTTCTAATAGAGATAACATCGTGGGTATAGTTAAAATCTTTTGCGTAGAATCTAAAGAATTCCCATAATAATTCACCGACAGATTGAGTATTAGCGGATGACCATTCCTGACGAAGTAAAGCAACATCATCGAAGAAGTAAATATTTCTACCTTCATAACTTGCTGATTCTGGGCTTACTGGTCGTAATGGAGGTATTCGCTGTAAGTTTGGTAAAACGGGTGGTTGTTTAACGTGAGCCAAGAAGAAGATGACAAGAAGGGTGAATCCGTAACTACTAAGCGTTCCCCTGTATGCGCTATTAATTTTACGCCTTTTACTCCAAACTTTTATGAAAAGGACGAGGGTTCTCAAACGAGGAGGGTCTATGGAGGCGTAACCAAGAAGTAAGCGAGTATTTTCTAATGCTAGCTGACCACCAAAGCCGATATCACATGCTATACCATACGGCAGAGCAGGAGTGGGTGCCAAGGTGAGCTTTATGATAGGAATACGAGCTTTCGGGAGTGGTTTGACGTGAAAGTTAGTTTCTATTGACTATTAAAAGCGAAATAACAAAATAAAGTGCCTACCATGTCTAATTAATTCGCCAACTATCGTCGTTAGTTCTGAGGATGACAGTGGTTCAGACGCCGGGTCTAGAATGCATTGTAGATCCTGTAAATTGTTATTAGACGAATAAATTGTCGTTAATTAATAAGATACCATATCTGTAGTCGTTAGAAAGCGTCACAACGTCCGCAAAAGACACCTACCTGAATTCCTCAAAGCGAAACCGTTTGCCATAGAACCAAAAGCTATTAGTCTTGCACCGGGACTGACTCTATCTATCAACTTCTCTAATAACAGCCTCGTCTGTTCTTTTATCGCGTACTCTTCTGCAGTCGGGAGAAGAGGCAGTATGAAACTGAATAAGCAGCCAGTTAGGTCCTGTGACAGAGCTTCCCTCATTGAGTTAGAAGCCCAATTATTCGCCA

>Wm5

TACATTTTAGCTTTGTATAATAACAAGTAAGCCACACTATCTTCACCTCCACCTTCTAATTGCGCAATTTTCTCCTTTGATATCTTGCTAACCTTGTTATCATCGAATTTATACCAATCATCTTGGTGTTCTTCACCTTTGTGTACATCTCCCCTAACCCAACCGATGTAGTGACCACCATCTGCGTTGACACCTTTGTGTGTAACTATGCCAACCAAATCGTACAATCCACTCTTTGAAGAACCAACGTCATTCTTCAAGTCCTCATCAACTAACTCATCCAGTTCCTTCTTCTCCTTCGCTTGATATACTTCTTCACTGAGTAATTCTGCATCACCAATCTTGTTTTGTTCATCTTTTATCTCATCGATTAACATCTCATCATTGTTCTTTGAATTGTTAGCCTTCTCCTCCAAGTCGTTTCTCGTCTTGATGCGCTTCCTCAATCTTCTTCTTTCTTCTCTATCCTTGTTGATCGAATAAAGTTTCTTATTGACTGGATTGATCTTGGCTTTCAAATCGTCACTAACTAATTCGCTCGCATCGTACTCCATTGGGAATTTTACTTTACGGAGAATCTTGGCTTTTTTACCTAAGTCAGCTCTCCAGTAGAATCTCACCAGATGAACGGTAAGATAGCTTGGTAGACGATTTATTCTGGACTTTTGAACATATTGAGCGTTCCTGTTAAGAGTTCCAGAATATTTCTCAATTTGTTCATCTAAGCCTTCACTCAAGCCTTGTTGGAGGTAATTGGTATTTGAAGAGATATTACAGCCCAGTTTGGTGAATTCCTCGATCGAATAAGTAGGTTCTTCCTCTGGCGCTTCAGGTGTCGACATTTCTTTCACATAGCTACCTCTCATGTACTGATCAACAAATGACTTGCCTTCCATACCAGTGGTGCCTGCTAATGAGCTGAGTATCGCACCCCAGGCTTCTTCTGCATCTTGTTGGGCAAACTGTCCGAATCTGTCTCTCTCTGTGAACTGTTGGTAGTTTGATCTTAACGTTGTCAAGAATGAAGTTGGTGTTATTGCGTCAGTTGAGTTGTTAAGTGAGCTAAAAAGCGCTTCTAAGGATCTACTGAGACCATTGTTCTGTGAGTTGTTTAAGTTGTTTTGTAATTCCGGTATAGCTCTCAGAACCTGAATTGAACTGTTCAAATAACAAGTGTTACCAAGGTTTGTCAAACCAACTGGGTTCTTCAAGGCTTGTGACAACTGCTTATCATCGAGATCTTCCATGAATACGACCTTTTCTGTAGGCGCCGAAGGTAATGGACCTGCTGCACCAATAACCATGAACAAATGACCTGGTTTTATTGTTGATCCGAGCTTCTCCATATCAGTATCATCCTTTAAGATACCACCTTTTATCATAACTTTTTGTCTAAACTGAGTGTTAAATGAGGTATACTTCACTAAGGTTCTTCTACTTACCTCTCCGGTGCCACGCCAGTTATAGCGTAGATATCATTCTTAAAATCCAAACCAGATGCTTGTGTATTTAAATCTAAATCTAATTTCTTCCCTGCGTGCTTAATTGATACTGGTATTTTAGTCATCTCTTCGTAGTAGTTAATAGAAAAATGACGCGTTCATTTAACTGTCAAATTATGTATTTTTCTGTGTAAAAATAATAATATGGATTTATTTATGCTATATTTAAAATGTAAAAATCAGAATATTCTAATCATCTCTAGATTAAAGACTCATTGAAATACTGACGTCAACATTTCGTTTGAACTCCTCTCTTCTACGAACGTCGATTATCAAAAATAAAAAACTAGAGAAGCAAAATGACAGTGAGTACTATTTTATAGCGTTATACTATTTTACTAACACTCACTCTTTAGCTTTATTACAGCTTAGTGTGGGTAATCAACTATCAATTAGAGATAGATAATTAACAGTTTAAACAGTTTCGGTTTACTCTCTGCTGAGTACGCTTAATTCGTCATAATATCTATCAACTCATCACACTCATAGAATCGTCACTTTCTTGGTATTAGTAGCACCAATGCCATTTGCGTTTAAGCAAAAGTTCTTTAAATTTTTAAGCACAAACCCATTAGTTGCTAAATTACAATACTCCTTGAAGATTTCTCTCATATTCACTTCCATTCTCTTCTTTGATGCTGTACAAAGGTGAGTTTAAGTGTAATAATCAGAAATATCATTACTTACATTATCAATAGAATGTTTAGAGTCGTTAAAGAAGGTCAAACAGCCAAAGAGGAACATGCTTTCACAGATGTACGCTCAGAAACTAACTTTGCAGCACGCAAGTACGTCACTTTTATATTCTAAAGATCTAAATTATTCACACACTTTTTAGGTTTTACTCACAACGTAACGTTTATCTCACCGGTATGTCTCACCTTTCTTTTCGTCATCTTTACAAAACTCATTTTCAAAAGGTTTCACCCTCTTTCTCAGCTTGTGGGTATTACTGCTACGTATTTTAAATTATCCAACTAATTCATTCCTTCTTCCATTAGAATTCTTTCACGCGTATTTGGTATTATCATGGACCTTATTCAATCACAAGAAGAATTGGGTTTGATGAAGGTATTCATTATACAATTATATTTAAACAATTTACTTACATTCTTCATAGAAAGATGGCCAATCATCAGCTGCTCAAAACTTCAAGAAGCAAATTGAAGCTAAAGATCGTGAAATTGGTTAGTATTATTAACAAAACCTTTCAACTTATTTACAAGCTTTCCAGAAATTCTCAAAAAGCAATCTACACAAAACAACAAAGCTATGAACGAACTCGTTGATAACGCTAATTCTTCATCAGGCTCAAAGAAGGAGAAGTAA

TAATGATACATTATGCAAATAATTCAGGTGCTTGACTGTTTACATCGACTGCCACATTCTCTACTTTAGATCTCCATTCGTTTAGACGTGAAGCCAATGATTTGACTTGATTTTTATCGAGGACTCTTGGTTGTACCCAATTGATGCTAGCTGAGCTTGAGACTTGGTCAATAGTACCACGGATAAGTTTAAGCCTTCTTATTTTAAGTTGAGACAATTCAGTAAAGAGATTACTCACGATAATGCTTTCATAATTAAGTGTTCTACTTCATCAAGTGGAAGCTTAGTTTCAGCACCGATCGTTGAGAAAGACATGTCACGATCTTTTGATGACCTTTTGAACACGCATTCGATCAATGCCATAAGGCATATCTTTTGACGAAGGAAAGCTTGACTGTCGTGTAAGATTGGCTTATATAATGTTACTTTTAGTGTTCTAAAACGCTATTACACCCACTTCATTTGCCAATTTAGAGGCATTAACCTCAAACTTGGTTATATCACCTTCGTTGAAAATGTAAATAATATCTTTCAACCATTCTAATTCGGTTCCATCAAGAGCATGCAAGATTGGATGAGTCAACTGAGTTTAATTAGAAAACTACTATATATTATTTTAAAGTGAGTAAGCTTACCAATTCACCGAAATTGTAAATTGTATCACCTAATAAGGCAGCGATGGCTAAATCGTGACTTCTTTGGATTTGCTCAGCTTTGTCAAGTTGGTCCATACCAGCACATGAGATATACAGTAATGTTGTACGGTAAAATGCTGCATATTCCGCTTTTGACTGATTTTTGATTAGGTCAATTTCTGAAGAGTTCCAAATACTCACTTTAAAGTAATCACCACTAACGCGTAGCCAAGCACCAGTGACACTGGCTTCTACGCCATCAATTGAATCTAAAACCCGCTCACAAACATCCATAGCTTCTTTAGCTTTGTCTAAGTTACCAAGTAAAAGGTCATAATGTGCTATTGAGATTTTTGCTAAAACGTAAGCCTCAGATTCGGTTTTAGTTGGTGGTTGCTCCTCATCGAACGATTTTGTATTGGTTGGCTTCAGTGCAGGTGGTAATGCAGCTCTTGGAACTGGTAGGGTGTCCAATAAAGATTCTAAGAAGTTTAGAGACTCTTGTGGAATCATCTCCTGTGCTACTCTAACGCCAATTTCCACTAAACGTAGTTGATTCAACTTATCCTTGAATGTTGAGATAAAGTTATTCCACAAGTCAACCAAATACTTAGAAATATCATCCTTCTGTATGATTTCTTGTAGTGTTTTAGTAAGCTGGTGGTATAAACTTTGGTGGCTGGTTAGCAATTATCTGATTCCTCGCGTAAGACGCACCGCTTGCTATAATTAATTTCTAATTTAGTCAAATCTTCTGTTAATGAAGCTGGCGCCTCTGCTTTTACTTTATGAATGAATTCTGTTGGACTCATAATGATAGTCGTGGTCAACGACTTGCACGCACACGTCATTGAGTGTGGATGAAATACTTAAATAACATAAGGGAAGAAAGAGTACACATTATTTTTATAAGATTGATAGTCATTTCCGAAGAACTTTACGAGATAAGTTTCTTCATCTGCGAGAATTAGAAGAATGTCTTAAGTGAGGAAGAGAAAAACTTACGTTTAATTCTATCAGTGAAAAACCTATGCAGAATAATAGCAAAAGCTATGAATGATAAAGGATTTGTCAAAATCATCTGTGTTCCCAATGCCCATAGGTAGAAACCTAAATACGAAGGGTGTCGTACGATTCTGTGGAAGTTTAACGAGAAATATTTTATAACAAAATACTCTTACCTGTAAACGCCATTAGTCACTAACTTATGATCTTTTTGTTTAAAGGATGCGACATGGTGTGAAAAGCTTTCACTCGCGTGTACCATAGCAAGCGTACGCAAGACCTGACCTACGACAACCAAGGCGAGACCTTAATTTATGGTAAGGGTAATTAAATGGTTCATACCACACTTACCTGAGATGATTAAGTAATGATTTTGAGAAATTGAAGGGTAGAACAATATCAAAAGGTAATGAGACACTAGACCGAAAGCGTGTGCAGCAGTGTAGCTATATCCATTGTTTAATAAGTAAGCTGTTAGTTTATTAGATTGCATATTCAATTAAGTATATGACTAACAATCGACTGATGTCTTTGTCATATTCCAACCTGCTGTACTGAGGAATTCCCAAAGATGAAACCAACAAAGTATGACTAAATATAGGGAAACTTGGGTTGAATGTAATGCTGTTGGAATTATAGCACTCAAATCTAAGTTGATAACTGATAAACCGGGAAGTAAGCTCAAACCAAAGACAGCACCTAAGGTAAAAGCAATAAGAGATATAGAAAAGGCAGAGTTCGTTAGACTACCATTGAGTCTTGGTGGGCTTTCAATTTGATTTGCGTATAACGCTGTACTGGGTATCTTAGCGGACATCGAGGACGAAGATGAAGAGATATATCGTATGAATAAATAAATATGAACGATTAATTGAATAAACTATACTATTACAGATTTTATCTATTAGTTCTAGCTTCTTATTATCTCTTGATTCCCGACGATGACTACTATCCCTTCAAGATTGGCACACGTAACAAAATCGTCAACTTCATTCGCAGAAGCTCACGCTAGAGCTAGAACTCTTTATCGTGACTTTTATCGTAGCGTGAGTGATAGAATAGATGATGGTTGATTGCTTTATTAAGATATACTTATAGGCACCAAGTATCTGTGCACTTTATTCATTAAATATTCCACCATCACAGATTAGAGCAAAAATTCGTCAGGAGTTTGAAAAGAACAGAAATCTCGACGATTTGAATGTGATAGACTTAGTTTTATTCAAAGGTAGACAGGAATACCAAGAAACAATGAACGCATGGAAGCAAGAAACTCACATTATGCGCTGGTTTGCATTGGAAGAAGTGAGTAATCTTTCTACTCTCATTCGCTATATCTAACTTTACACAGGCACCACGTCAGTTTGATAACTTTATTATTTATTCAAAACTAACATAATACTCACAGCTAGACCACAAACTTTCCTTGAAAAATTCTACGCTGGGTACGTCTTTACTGTCTATCCTAACTCTTCTCTTACACATCCTACTCAGTCGCGATGAAGATCAAGTATAAATTTAATACATGGCTTTTACATTAATAATGTGATTTTCTATGTTGGTTTTCTGCTTTCTAAATATGAGGCTCTCAGAACAGGAAGCTCGTATTCTTCAGATTGACCCCTACTACAGTCTTTCAGCTTATCAAAGTCATCGTAGTCTTGGCTTGGTGAGGAGGAGATAGTTGACACATGACCATTGTTTGGAATAGTTGATCTTAGTATGCTCAATAAAGTATATCAATCGTATCCCTATACTCACCGTTGAAGCCTTGCTCTGAGGAATGGAAAGCAAAAGAGTAAGCAACGGACGTACTCTTTCCGGGATTCTTCAGCAAATCCGAAGAAAAAAAAGAAAATAATAGCTGTTATGATTGGCAATAACCAGCTTGCTATGTTCGCACCTAACTGATGTTCGTTTAGTTCGGAAAGTTTTACCTGGTAAATCTTTGAAAAATAGACATGAACTTGGTCCCAGCTCTTGTAAGGCAATAGCCGTCCACTGTTTGCTTGAAGGTTGACGACTAATTGTACTACTGCAGCAACGATGGTGAGAATAAAAATCGTCATAGCAAGGCCTATCAGTCTGATAAATTTACTTTTCGTCAACCCGTACTTCTTCATTTTAAACAAGTCCATGAAAGTTTCTCGATATTTGTAGAACGCCCTGAAAGTCTTAACTATTGAGTGTTAAACTAGTTCAACATTAAGTGTGAAAGGACACACATGAATAATAAGCCGCTACTATTGCGCATATGAAAGGAAGGAGGGACGTATGAACAAGAGAAAGCCAGGAAGGCCAAATGACTGGCATACAACCTATACCCTCAATAATGTTAAACCGACGATCTTGAATGACCACATGTAAAGCCATGTAGATGATAGGAACTACTACACCGTAAGTCAGATCAAGATATAATTTTTTTCTCATATCCTTTCGTGAGGTGCTGACAACGGACGCTGTAGCTATACCAGATAAGTTGCGTAGAAGACAAGCGGCGGTAGCCGGAATTGCGATGTTTGCGCCAATAACCAATTTCGTCGCTATGTCAGGTGAGGTTATTGATTCCAAGCAAGTAAATCACTTACTGATATCACACCAGACGGGAGTATGTATGTCCACATTGTCATGCCAGATGATAGAGTTGATGAAGTAGATAAAATTGACGCCAATAATCCATACTGTAAGTATAAGAATGTTGGTATTGCCGACTCGATAATGCCACGAGCAAGGGAGAATCGTTATAATGACTGCTATTATAGATATTATTGGGATTGCTACCAGGGACATTTCTGTTTAAAAATGATCAAAATCGTATTTATGGCTTTTTCTGGTTCGATTCATTGTCCTTCAAATATTTCGCCTCAAACAATGAGTTTTAGGCAGGAACGCCATTGTGCGGATAATCATGCATAAAAAACTCATTGTTCTTGTGATAATAAAGAAAATGAATTCGTGAACAATGGGAGATAAACTTGATAAAGTTCTGATAAGAAAATTCTCAGAGTATAAATAGAAAGCAGTTTGCTTGTGCTTCTTCAACAATCAAACAACAAGCATGATTTCAGATCTCGAAGTTCCAACTCTCCAATTCGACCCAGTACACATTGATGTTAAAAGAGAGCCTCCAAACGAAACAGAGAAGGACCCAATTGGTAATATACCTAATTGCGTTATCACATAGAATAGTGAGTTTTCTCAAATATGATATTGAAAATTGAAACAATCTTATCTGCAGCTACTTCATACTCCTACTTCGTCTGATTAAATTGACTTTATATATATATGTACGGAAAATGAATGATACTATACAATAAACTATGTGATTTTACCTTTCTCCAAATCCGACATTTGTTCGATTTGGATAAGGTGCTATGGGTTGAGGACCCACGTAAGGAGTTGGGATAGGATTGCTCATTGGTGTGAAAGGTGCCCCTGGGGTTTGTATAGCAGTTGCTCCAGCTCCCATACCTATACCATAAAGATATCCCATACAATGAGGTCTGATGGTGAAATTAGCAAAGTAATAAGTTTCGGAAAGAATGTCTTACAATTGTCTATCTCTTTCAGATGTACCATCGAAGAATTTTGTACGCATAGAGAAGAAGGAAGCTGATGTGACTAAGGAGTCTGAACCTGCTTGATGTTGAGGGCCTATTCGCATCACCTAATGAAAGTCAGAGCGGCTGATATAATGGAACATGCTTATTATTACCTGTAATTCATCTGCAATATCTTGTAAACCACCCTTTAACAACCTATTGATTTTCATAACGTATTTTATGTCAAAAATGCAAGGGAACCAAATGCGAAGTAGATCAAAGAAGTCGTTTTCATTTGAAGGTAAGGGCAATGAAGTGAGAACTTTGAGTAAGTAACCAAAGTCATAGCCACTATATTGAGTTTGTAAACGAGTTACATCGCGCTTGTTACTCACCTGTGAAACGATATCCAGTGAACGTGATCGAACAGAACAAAACCAGAAGTGACCAGTTTCTCTCCAAATTCTTCAATATCAATACCATTAGCTTCTAGCCTTTTAAAATTAATACCAGATTTTGTGAGTAAATCTATTGAATCTGGTGCATACATATCATCGCTATGTAGATATTAGAATGATGAATGATATGATAAAAGAGGCGTACTTTAAACTGAATTTAAAGTTGAATTGCCAAGTAGGATACTCAGGCATATTGCCGTCTGCGTCAGCGATAGTGATACCTAATTGTATTATCTTCAATAAATCCACGTTGCATCGCAGCGTTTGATAGTGATAATCTGATGAATTCTTAAAATTTCCAATTGGTCTAGCTACAACGCCAGGGAATTCAGTATCCTAAAACCGTCAGTTTGGATGTCAAATACGTCGATTGAATTCACCATAGAAACGTAAGGATATCGATCGACTAATTCACGCAAAATATCTAGCTCTGCGTCTAGATTATCTGCCCAGACTTCACGGATCTGAGTTTGCATTTCTTGACTGTTGCTAACCAAGTCGACGAATTCGTCAAAAAATAAGAAATTTGAATGGTTTTACAAGATAAATATAAAGCTAAAGCTTCAAAAGCATGGAAAGCTTCAAGAGGTTTAAGTACCGCTCCAAAACCCACCACACGACGTCTACAGCCACCAGAAGTAGATAATCAAGCGGAATTCCCTGATTTAAAGAAATCTGATAGCTCAGATGGCAGTGGTAGCGAAAATTATAGCTCAGAAATTAGTCAAAGTGAAGATAAGCTTGCAGTTCCATCTGCTAGTAATAACAGTGAGTTTACAGGGGATACATTTAGATAAAGCTTACTTTATTAGCCACAAGGCGTTCAACGAACCCAAAATACTCTAGGCGGAAATTAGTTGATAACTCCAGTAAATACGATGAACCCGCGACCGAGCAAGAGGAAGGTATGAGAGGGAATATGTAGATCCATTAGACTGACTATACGCAGTTTCATCAGAAGAAGAGCGTGATCTCTCGTCCATCACAATGAAACTTAAGGTAGATGACGATTTTGAACAATATATAAAGCGACCTACAGATGCAGACGATGATGATATAGATTATGATTTGGCTAATAGACACAAGAAGAAAGAACCCCAATTACTTCCTATTGATTCAGAGAGTTCAAAAGCATTGGAGGAGATGCGTAACGATAGGATGAAGGCAGAAGCATCATGGAGTGAGTAACAGCAATCTTTTACATCTTTTGACATCCTTAAAGATCTTAAATCTCGTCTGAGAGGTGATTCAGGTAGACAATACATCCAGCGATACGAATCGTCAAGGAGAGACGTGAATCATTTAGAAGACAACGATTTCTTTGAAAGTGTGGGCGAACCAAAAAGTAGCTCACCAACAAAAACAGCGCATCGCGTTAAAAGTGAAACAGTCGCTATCGAACGCGATAATCAGCCCATGAATATAAGCACCATAGACACAAGGTCTGATCAAAATTTTTTGGATGAATTAATATAAATATAAATATTAATAGATATACATGATATATAACAAGAACAATTAAAGTTCAAAGATATCACAAACAGCAAAGTAGATATAATTCTCAATGATTTGATAATCACTTAAAGCAAAATCATTGGTTTACTTCTTCAAGAGTTCCTTTTCTACTCTCTCTTTGAGATAAGCTTGTTGGTATGGCTAAAAACGTCAGATTAACACTTTCCATCATTAATGACTAACCTCTTTCTCATCATCAGTAAGCTCATTCCATTGTTTAGATGCAGCTTTCATTGCTTCTTGGGAATTACCAAGCTCACGTGAAAGTGATGGAATGACTTCCTTCATGAATCGGATGCGGCTGTTGGCAATGCCCTTTGGCTTGAGAGACTTCTCAGCTTGACGAGCTTGACGTTCATTTTCACGAATTTCTTTCATGTATTCTTTTGGAAATGAAAGAGGCTTCATACCATACAAGCGACGCTCCTCATTGATGAGACTGATATCGGAATGGGTCTTAGACATCCACCAATCTCTCTCCTTTGGCTTATAAAGCTTGTAGTCGTGCTGATAAGCTTGCTCGAAGGGCTACGCTCAATTAGCAGACATCATCTTCGACAACAAATACTCACTTTCTTGTCACTTTCAAAAAGGCTTTCCCATGAAGGCGCAGCAACTTGTTGACCGAACTTGATAGTATCAATCTTGAGTAGTGGCTTGCCTCTTTTCTCTTTCTGGATGAGATGGTGCATAAAACGTAGATAAGGTGACTTGGGCTTTGCAGGTGGCTGGAGTGCTGGGTCACCTAAATATGATGTTAAAAGTATCTAAGCAGGTTTGAAATTTGCTTACGTTCACGCTCTCTCAACTGGGTAGCTGTGGTGAAGAGTGTGCGCTTGAGTAGCTCAGGTGAAGAGCCAAAGAGACTTCCAAAGGGTGCAAGATTTGCCAAAGCTATTACTATAAGTTATAATTGTATGGCGATAAGTTCATCGTTATACTTACCTTTGGTTGAGTTGGATGCACATAATGCCGTGAACTTTGCGAGGGCTTTGATAAACATCGTTGATGGAAGAAACTAGTCAAGAGCGAAAAGAAACTCGGTTTTGTTTTTAAGGACGTAACTTAAAAACTTAAAAACTCCTTGTCTTTGTCTTTATGCATGAGAGTGACGTCATCTTTCAACATTTCAATCGCCCATTGGCCGAAGCCCCATTCACACATTCTAGAATAAACAATTATGTTATTAGAATAAACATGAATAGTTATCGCATAATTTACAACAGAATTTACAAGCTGGGCATATCAGTGAGATACAAGAAGGTCATGCGTACTTACAAGTATTGAGAAGGAGCGACTCTCGCAACAGCACCTCTGTCCTGATCTGCTACTATAGTAGTTGTAAGTAGCCGTATTATTTCTTCATAGAAATACGTACCTGCATCAAAAATTGCAAAATTGTTTTCTGTGACAGGTTCATTCTCTTTCACTTTAAGAACGCTGGCAACGTTTCCTGGTCATTAATTAGTATGACTGCGGACCAATTTCAGCGTTCCAACTAACCGCATCTATAACAGTAATTAGGTGCTGACCAGACAGTCACAAGGTCATTGTCCGGAAACATGAATTTGCTAAGGCGAGTAAGTAAAATATCAGCTACGATGCTTGGTACATACAAGCCCTCTTGAATAAGCTGATGAGCACGGGCGATCATGTCTAAACCATTTATGTGATTAAACTGCATATAGTTGGAATATGATCATAGAATTAAAGCCAATCTTACTTCTTTTGTAACTTTGCTACCAAATAGCCAGCCAGCCCCTCTGGGACTTATGCTCCAATGTTCTACATCGTCAGGATCCGACCACATGAGGTCTAAATGGTCGCATTAGTGAGAAAACCACAACAAATACCTTTCTTACCACAAAACGGTCCATCATGAGGTATCTCTTGAGCTCGTGCTTAAGGTGTATAAGTTTTAAATTGAGTGGTCCAACTGAAGCTCACCAATTACGCGAATTTGATCTAGAGTTCGTACGTCTGGTGACAAACCTCCATGAACACATAAGATTTTACCATCAATGATCTTTGGCCATTTAGCCTGTGACTAATTGAATGGCGTCAGACTTACAGCAGCTAAATTGAGATAGTCGAAGACTTGACAGCATGCTTTCCATACACTTGCATTGCCATACTTTTGTTGACATTCGTCTAGACTCTCGGTCAATACTATAGCTCGACTATGAAGCCAGCTTACCATAAAATCCGTATACCTGAGTGATCTGCCTAGATTCATGATTACCACGCAGTAGAGTTATCCTATCAGGATACCTTATAGCTGTTAGATGAACCATAATAGAATTATGTCAACCAACCTGGCTTTGAGGGCCATAAGGAGGGTAAAACTCTCTAAACTTTGATGACCCCTATCAACAAAGTCACCCTACCCATAATCAGCATCATATTTATTAATAAGTTTGTCTCCTAACCATGAAAATGTAGTTGGTATCCCTGCAATCACCACCGACCCGCAGAAGCTCCAGTAAATCCCAAAACTGACCGTGTATATCTCCGCAAACGGTGACAGGGCTTGATACGGGTTGTATATTTGATTCCTCTACCAGTAATCCTAGTACTACATCGCATAACATCTTGATTTGGGATTCAGCAAGATACTTTCCCGATAAGATAGTGTCGAGCCATTGTTGAGGGTCGACTGTCATTAATAAAAGACTCTAAGGATACTTATGTTTTTATAAAGTATTTCGTCCTCGTGGTTTGCACAAAACTCTCGCTTTACACGTTACGTCGATGGTCGCTAAGCAACATCCGTTTATTAGAGGGCTTTCTGAGCTGGTTCCACACCCTTCACCGTTGTTATCTGTTTCACAGGACAAGCCAGAAGATTGGTTCGATTTCAAGCAACCTTCACTTTGGATCGTAATCGCATCAATTGTATTCAACCCCTTATTCTGGAATATCGTGAGTATAGAGACACTATTAAGCCTTTGATTCTGATTCTTTTCATAGGTTGCTCGCCAAGGTTTGTACTATCTCGTTAGAGATGAGTTTTGAACTTACACGTTGAAAAAGAATATCACAATAAGCTACTCACAAAGCTCGCTGGTGGTAGACCATATCTTGGCTGTTACCTCCTTGCTGTTACCATTTTCTCATTAGGTATCTTGCGTGATCATCTGTACGTGCAGTATTATAGTAGAACTTCTTTCTCATCTTTCTAGGTTTCAGAGTGCTTTAGCTGAACAACCAAACTTGGCTCCACATCCATACTACCCAACTGCGCATGTATACATTGCTCTCGCTTTGTTCCTCTCTGGCCAAATCTTAGTTATAACTTCTATGCTTGCGTTAGGCGTTACTGGAACCTACTTGGGCGACTACTTTGGTATACTTATGAGCCACCGCGTTACAAGTTTCCCATTCAATGTTGTTGAGGATCCAATGTACATTGGCAGTACACTTTGTTTCTTCGGTACTTCCATTTGGTACAGGTCACCTGCTGGTATCGCAATCAGTGTATTCGTCTTCATCGTATACCAAATAGCTCTCAGATATGAAGGGTGAGTGGTGCTTCTTATAATAATTCTATACTCACAAAATCCAGTCCATTCACTGCTATGATTTACTCAGCTAAGGACAAGAAGACAGAAGAAGCTTCACCAAAAGCAGCGCCAGTGAAAGCCCCAGTAGAGCCAGTAGAGTCTTCTCCAAATAACTCACCTGCTTATAGGACTAGAAGTCGCGCTAGAAAGGTCATTGACGAGTAAAATTTTCAAATGTTTTGGTTTTCTCAACAAAATCGAATAGGATTGTAGTGTATGAATAAAATTAAATATCTATCTAATCATTTATAAATTAATTAACCAAAGTGAATGTTCTCATCGTGGAATGCACTAAGTAATCTGAATCCCAAAGTTAGCATGTATAGGAGGAACGGATCGCACTTACTTAAGGAAATCATCAACGTCCATTTTCGCGGCACGGCTTTCTGCAAAGCCAGTTTGTACTAATATCCTTTCGACTCTGCTCTTAATGTTCTCTGAGTCGTCTATCATCTACATACCAGTCAATTGATTAATATCTCTGGTAGTAATCCAACATACTTCATTTTTCTCAGTCAACCAAGCTTTCCTATTGCTTTCTAGCATATCATAAACTCCAGCAGCGGTAAAATTTGATCTGCATGTCTTGTTCCTTCTTGTGAAAAGCACTCTGGTGAGACTATGAAACAGTTAAATAGTTAAAGTGAAAAAATAAGACGTACCCGTCAAATTCTTCAAACTTGACTGGTGGTGGGGGATCTAAGGGGACAATTCGTACAACGCTACTTTCGACTTGTGGCGGTGGTCTGAAGTTGCCCTTGCTGACTTTCATAATATGATCGATTTTTGAATATAACTGGGCGTTGGCACTGAGGCGACACCACATCTCAGTACCAGGTCTTGCGCATAATCGCAAGGCGAATTCACGCTGAAACATCAGTATCGCGACCCTACAGATAGGTCTATGGGAAAGAATACGAAACACGATGTGTGAAGAGATTGAGTATGGGCAATTGGATATTCTACATTTTAGCTTTGTATAATAACAAGTAAGCCACACTATCTTCACCTCCACCTTCTAATTGCGCAATTTTCTCCTTTGATATCTTGCTAACCTTGTTATCATCGAATTTATACCAATCATCTTGGTGTTCTTCACCTTTGTGTACATCTCCCCTAACCCAACCGATGTAGTGACCACCATCTGCGTTGACACCTTTGTGTGTAACTATGCCAACCAAATCGTACAATCCACTCTTTGAAGAACCAACGTCATTCTTCAAGTCCTCATCAACTAACTCATCCAGTTCCTTCTTCTCCTTCGCTTGATATACTTCTTCACTGAGTAATTCTGCATCACCAATCTTGTTTTGTTCATCTTTTATCTCATCGATTAACATCTCATCATTGTTCTTTGAATTGTTAGCCTTCTCCTCCAAGTCGTTTCTCGTCTTGATGCGCTTCCTCAATCTTCTTCTTTCTTCTCTATCCTTGTTGATCGAATAAAGTTTCTTATTGACTGGATTGATCTTGGCTTTCAAATCGTCACTAACTAATTCGCTCGCATCGTACTCCATTGGGAATTTTACTTTACGGAGAATCTTGGCTTTTTTACCTAAGTCAGCTCTCCAGTAGAATCTCACCAGATGAACGGTAAGATAGCTTGGTAGACGATTTATTCTGGACTTTTGAACATATTGAGCGTTCCTGTTAAGAGTTCCAGAATATTTCTCAATTTGTTCATCTAAGCCTTCACTCAAGCCTTGTTGGAGGTAATTGGTATTTGAAGAGATATTACAGCCCAGTTTGGTGAATTCCTCGATCGAATAAGTAGGTTCTTCCTCTGGCGCTTCAGGTGTCGACATTTCTTTCACATAGCTACCTCTCATGTACTGATCAACAAATGACTTGCCTTCCATACCAGTGGTGCCTGCTAATGAGCTGAGTATCGCACCCCAGGCTTCTTCTGCATCTTGTTGGGCAAACTGTCCGAATCTGTCTCTCTCTGTGAACTGTTGGTAGTTTGATCTTAACGTTGTCAAGAATGAAGTTGGTGTTATTGCGTCAGTTGAGTTGTTAAGTGAGCTAAAAAGCGCTTCTAAGGATCTACTGAGACCATTGTTCTGTGAGTTGTTTAAGTTGTTTTGTAATTCCGGTATAGCTCTCAGAACCTGAATTGAACTGTTCAAATAACAAGTGTTACCAAGGTTTGTCAAACCAACTGGGTTCTTCAAGGCTTGTGACAACTGCTTATCATCGAGATCTTCCATGAATACGACCTTTTCTGTAGGCGCCGAAGGTAATGGACCTGCTGCACCAATAACCATGAACAAATGACCTGGTTTTATTGTTGATCCGAGCTTCTCCATATCAGTATCATCCTTTAAGATACCACCTTTTATCATAACTTTTTGTCTAAACTGAGTGTTAAATGAGGTATACTTCACTAAGGTTCTTCTACTTACCTCTCCGGTGCCACGCCAGTTATAGCGTAGATATCATTCTTAAAATCCAAACCAGATGCTTGTGTATTTAAATCTAAATCTAATTTCTTCCCTGCGTGCTTAATTGATACTGGTATTTTAGTCATCTCTTCGTAGTAGTTAATAGAAAAATGACGCGTTCATTTAACTGTCAAATTATGTATTTTTCTGTGTAAAAATAATAATATGGATTTATTTATGCTATATTTAAAATGTAAAAATCAGAATATTCTAATCATCTCTAGATTAAAGACTCATTGAAATACTGACGTCAACATTTCGTTTGAACTCCTCTCTTCTACGAACGTCGATTATCAAAAATAAAAAACTAGAGAAGCAAAATGACAGTGAGTACTATTTTATAGCGTTATACTATTTTACTAACACTCACTCTTTAGCTTTATTACAGCTTAGTGTGGGTAATCAACTATCAATTAGAGATAGATAATTAACAGTTTAAACAGTTTCGGTTTACTCTCTGCTGAGTACGCTTAATTCGTCATAATATCTATCAACTCATCACACTCATAGAATCGTCACTTTCTTGGTATTAGTAGCACCAATGCCATTTGCGTTTAAGCAAAAGTTCTTTAAATTTTTAAGCACAAACCCATTAGTTGCTAAATTACAATACTCCTTGAAGATTTCTCTCATATTCACTTCCATTCTCTTCTTTGATGCTGTACAAAGGTGAGTTTAAGTGTAATAATCAGAAATATCATTACTTACATTATCAATAGAATGTTTAGAGTCGTTAAAGAAGGTCAAACAGCCAAAGAGGAACATGCTTTCACAGATGTACGCTCAGAAACTAACTTTGCAGCACGCAAGTACGTCACTTTTATATTCTAAAGATCTAAATTATTCACACACTTTTTAGGTTTTACTCACAACGTAACGTTTATCTCACCGGTATGTCTCACCTTTCTTTTCGTCATCTTTACAAAACTCATTTTCAAAAGGTTTCACCCTCTTTCTCAGCTTGTGGGTATTACTGCTACGTATTTTAAATTATCCAACTAATTCATTCCTTCTTCCATTAGAATTCTTTCACGCGTATTTGGTATTATCATGGACCTTATTCAATCACAAGAAGAATTGGGTTTGATGAAGGTATTCATTATACAATTATATTTAAACAATTTACTTACATTCTTCATAGAAAGATGGCCAATCATCAGCTGCTCAAAACTTCAAGAAGCAAATTGAAGCTAAAGATCGTGAAATTGGTTAGTATTATTAACAAAACCTTTCAACTTATTTACAAGCTTTCCAGAAATTCTCAAAAAGCAATCTACACAAAACAACAAAGCTATGAACGAACTCGTTGATAACGCTAATTCTTCATCAGGCTCAAAGAAGGAGAAGTAATGCCTATGTCAGACATGTGTGCTTTTTAGAGTTCTGAACTCACACTACGTCAAAGAAAGGCAAATCTGCCTTGACAAAATCACCGATGACGATTTCTAATCTACGCTGCTCAGGTCTACTATATTTAGCAAGTATTGATGATTTCCATTGATACTCACTTTCCCTGAAATCTTTTGCTGAGTTCGGCAGCCATTCTAGGATCCATTTCTATTACTTGACAAGACTTTGCTTTTTCAAGTATTCTAGCAGTCAAATTTCCAGTACCTGGACCGATTTCGAATACTTTGTCTGTTGCTCGCAAAGAAGCTTTATCAACGATACTTTTGAGAGTTCAGAAGTGCAGTAATAATCTAATGATACCCACGCTTGAGCTACTAGGGGATTCTTGAGAATATGCTGACCGAATCTGTCTGTGTTACTGTCCTAAATGAGTGATTGTAGATGCTGATAACAGAATTGCCTACAAAAGTGGATTGCTGGTACCGCCTGAATTGCTATCTGATTTAGCAGCAGGCTGATGGCTCCTGGAGAACTTCTCTGTAGCTGATTTCAATGCTTTTGGCATATTTTAGATGACGGCCAAAATTAAAGAGGAGAGCTACGATCTTCTTTCTATCACAGCAACGCAGTAAGAAATCAATTTATTCGCCTGAACTGGCGTTGCAATAAAAAAACTACTTAGCTACAAAATGTAGGTGGCCATATCTCCTTTCCCAGCTTTCCTAACCCAGCGGCAGGAATACGATAAGAAGAAACGTCTTCACAGCGAAGAAAGGTGGGTAGAACAGCTATCCCAAGTAAGCAGCTCTGATCTATTATTTAGGAGAGAACTATGAGCTCCGTGCAGGTATGCTATCACGATATCTCCACACTACCTGCAGTTAACGTAGCCAGACTTAAATAGTGAATACCGCGACAAAAGAAAGGATGCTATTAAGCGTGTTATCGCGAATATGGTTAGTTATTTCTTATAGTGCGTGCATCTCTGATATCTCTCAGACTGTTGGAAAAGACGTATCGGGTTTATTTCCAGACGTACTGAAGAACATGCAGACAGAAGATCTGGAGCAGAAGAAGCTCGTTTATCTTTATCTTATGAATTACGCAAAAACACAGCCAGAGCTCGTTATACTTGCCGTGAACACATTTGTCAAGGTTGGTGAGACAATTGGCTGTGATTTATCGCTTAACAACACTTAGGATGCTTCCGATCCTAATCCATTAGTCCGTGCATTATCTATAAGAACTATGGGATGTCTCAGAGCAGAAAAGATTATCGACTATCTCTCAGATCCACTTGCTGCAGGTCTACAAGATGATAACCCATATGTACGCAAAACAGCTGCTCTATGCGTTGCCAAAATGTACGATCTTAAACCATCACTTGCGATTGATCGAGGATTTGTTGAAACGCTTCAAGAACTTGTTGGTGATCCAAATCCGACTGTAAGAAAATTACTGTCGTATTATTCAATCATCTCATTATTCCCCAGGTTGTTGCAAATGCCGTCACTGCCTTGACTGATATTCATAATTCCCCTCATCCAGATTCACCAGGTTTCATAATTGATAGGGACATACTTAACAAGATCCTAGTTGCTCTTAATGAATGCACCGAATGGGGCAGAATATCAATATTGAGCGCGTTGTGTCGCTATACACCAACAGAGGAGAAAGAAACTGAATACATTTGCGAACGTGTTTTACCACAATTTCAACATGCCAATGGTAGTGTTGTTTTGAGTGCTATTAAAGTCGTTATGATTAATTTACAGCGTTTACAAAGGGAAGACTTTATAAGACAGCTCGTTCGTAAGATGGCACCTCCTCTAGGTAAGTACAATGCATTTACTACTTGGTAATTTGACAATTTTTCAGTCACTCTTGTGGCATCAGAGCCTGAGGTACAATGGGTAGCTCTTAGAAACATAAATTTGATATTACAAGCTCGTCCAGACGTTCTTTCTTCAGAATTGCGCGTCTTTTTTTGCAAGTACTCCGATGCACAATACAACAAAGTTGAAAAATTAGACATATTGGTTAAATTAGCTAATGAGAACAATGTCGACACTCTATTAAACGAGCTTAAGGAATATGCTAGTGAAGTTGATGTTGATTTCGTTCGCAGATCGATAAGAGCTATAGGACGGTGCGCAATCAAAATTGAAGATGCTGCAGAGCGTTGTGTACAAGTTCTCGTTGACTTGATCAACACAAAAGTATCATATGTTGTACAAGAAGCAGTCATAGTCATAAAGGTGAGAAACACCATTCTCGAACTTGGATTTTAGCTGACCTTTATCTAGGATATATTCCGTAAATACCCACACTCATATGAAGCAATTATTCCAACACTTTGTGAAAACCTCGAAGAAATTGATGAACCTGAATCCAAAGCGTCATTGATTTGGATCCTAGGTGAAAATGCAGAGAAAATAGTAAATGTCGAGGAGTTATTAGAGACGTATCTGGATAGCTTTATCGAGGACTCTTACCCAGTGAGTGAATATATTAAAGCATGTGATAAGCTCATAATGTTAGGTTCAATTACAAACCTTAAGTGCGATAGTAAAACTGTTTTTAAAGAAGCCAGAGGGACCTTCTCAGTCATTGGTTCAAAGGGTTTTGACGACAGCTACAAGTGACTGTGATAACTCAGATGTACGTGATCGTGCATTTATTTACTGGAGACTACTTTCAACAGATACTGGAGCAGCAAGAGTGAGTGCTAATTAAGTGCTAGAACTGACTAACGATTATAGTCAATTACTATAGCGGATAGGCCACCTATCGTCATTCCAAACTTAACAGTCTCGCAGGCAATCCTAGAAGAACTAGTCCAAGAAATTTCTTCCCTTGCAGCCGCATATCACAAGCCAGCATCTACTTTCGTTGGTAAAGGTCGTCTTGGTATTGAGTCAATAAAGAAGATTAATCCGAATGAAGAAAATGAAAATATTTCGAAACAAAAAGCATTGAACACGGTGGCACAAGGTTAGTATCTATGAAATTTGTGATTGCGCATTCTTCTAAACTCTCATGTAGGAGTTAAAGCAGAAAATTTATTAGACCTTGATGAAGATGCCTCACCATCCACGACACAAAGTCCACAAGAAAGCTTCAACAGTTTCACAGCCAATACGGCGACAAGTAATATTAACCATGTACCAAGTCCCGCACAGTCTAATCCTGTCGATGACTTATTAGGCTTATTCGGAAATGCCACCGTTTCGAGTCCTAGTCAGTCCGTCGGCGCAAATAACGACATTTTCGATATGACAACAACAAACAACACACAAGGGCAGAATAAGAATAGTGGTTTAGAAGATCTCCTTTTTTAATGTAATTTTTGTTCTCATACACTTCCTCGCATTTGGCCCTATCTCTAATTTCAACCATCAGGAGTGAATTATTAATTCGAGAGATAAAGAAGAAATAACTCTCGTAAATAAATAAAATTAATATACGTTAAATACAAGTACTCGCATAATCGTACTAGCGACTCGTAAAGCTATATAGGAAGCTTCTTACAATGATTGCGGCAGACGTAGTAGTACATAACGTTTTAAATGAGATCTCAGTAGATGGTGACTGGGGTACGTCACTTTCGTTATCAGGCGCCGTCTGCAGGTGCCGGTGTTCGAATCTCTGGTTGCTAACAAATCATAGGCTCCGATATACAAACTCTTGTGAAATACATCAAAGCACAGTTAGATCCTTCGGTAGATATCAACAATGGTCCGTTACTTGAATTCTTCTGGAGAATTCTCACTCAACAATCCTCATTTCAAATTGGCTTAGCAACAGAACAGTTGAAGCTAGCCTTAAAGGAAAAGGCCCCTCCGATATCTTCTAAAGATGGTAATCAAGTTATCATGTTTGAGCTACTCAACTTCGATGATACACAAACATCTCTGGCCTCCCTTCTTGAACGATACGGTAACGATATTCGCGTTCGTACTGATAGTGACATGAATTGGTTTGTGATAACTGGTCTTAATCATAAACCAGCAAAACTTAGCCAAATGGTATTGAGTGTGCTCACTTTTATCTCTAGAAGTAGAGAAAATGGTATCACCTTTATGCAGATCAGCAAGATGACCGGTTATGACAGCAAAACTGTATTTTATTGTGTCAAAATACTTATAGAATTGGATCTTGTGTAAGTTAACAGTCTGATCTAGCCCTCAATTGACAGTATCATTAGCGTAAAAATGCACACTGTCTACAGAGGTAGCACCACAAATGTTGCTATACATCGTAAATTTTTGGCCAGCTCACCACACTACCGAGCACATGCACTCACAGAAGCGGAACCTGCAAAAAATGATTCCGACAATGAAGCAATAGATTTTGAAGAAACAGTACAAGGTCGTGACGCCTCAGGTCTACCAGAATTCTCAGAATTAAGTAAGGAACATCTCACCAACAACAATTTGTTGAAAGGCCGTATCTTAAAACTTATCAATAGCTGTCCGAACAAGATTATTCAACATAAGTATATATTTAACAAAATAGGATTTAAAACAAATAGTAAACCTATTCAAAGACTCGTTGCACGTCTCATAGGCAGAATGGTTGCAGAGGGTTCACTCGAAAAATGTGCTACCAAAGTTGGAGGTAGGGACACACGTGTTAACTGTTTGCGAATTGCAGATGATGATAACGGATCAAAGTCGTCTGAGAAAGTCATTGAGGAAGATATGATTAAATTAGAATCTGATGATGAAGATGATGATGATGACGTTGTTTCAGATAATTTTGGAGCTGGGATTGCTATCACAGTGGAAAAACAAATATTAAACCTTATACATGAATCAGGTATTGAAGGGATGACTATCTCTGTAAGTTGAAAAGGTAAACAGAATGTATTGTTTAACATCTTGCTAGTCAGTTATTAGCGCGCTTCCTACTATCAATAGGAGAACTGTCGAAAACATCCTTTTGCGTCATGAGACAGATCATGATCCAGCTAGCCTCACCGACCTTGCACTAAAATCAACCACAGAAATGAATGGTCGAGAGCGCAGGCAACGATTTTTTACTCGCAAGAATTATATACTTTTAGCAGAAAAAGAAGGGTTAGATATTCCAGTTGAAGACTTGGTTTCCGATCAATCTGGTTCATTGAACGTTATTAATTCTGCCTTGGGGTGTTCGTCTCGTCAAGAGTACCAGGCGTCAGCAACTCGCATGATAGGATCGATCGGTGGGCAGGTGGCTACTCCTAAAAAACTTGGTAGACCTCCGAAAGCGGAATCATCCAAATCAGCAATGCCTACACCAAAGCCTAAAGGTCGTCCTCGTAAGTCTGAAGCAAGCCAAACACCATCAGAGGTACCGAAGAAGAGAGGCCGTCCACGGAAGTCCGAAATCATCGAAGATCAACCTAAAAAGAAAGGTCGTGCTTCAAAAAATACAATAGAAATAAATGATGATGATGATGATGATGAAAAAACTGTAAAGGACGTGACAAAGAAACATTCTGAATTACAGGAAAATGTATCTGAAAGCATACCATTGAATGCTACGCCAGAAGAAACACAGCCCCCATCTGAAGTGTCTTTAACAACATCACAAACTGCACCTCAAAGAGCTTATCACAATCCTGAACTTGATATGGTCGGTGACGATAATAGTCACATGTCAACGCCTAATAAAAGACAACAACTAAATACACAAGAAGTCGCACATAATACTATCAATGTTACAAACGATGTAGATAAGGATTCATACCTTAAAAAACGCAAACTTAGATCAAGGAAAAGTGGGATCGCTCCTATTTTAATTGATGTCGAAGATGTTAGTCAAGATCATATATCAACAAAACGAAAAAGAGTTCAAAGTCCACAAGATAGTGATGAGTCCGATGCATATGAACCAGGTGAAGACCATGATGAAAAGGAAGACGGGACTTCCGAAAATCAAGGTGTCGAAGATGAGAAGGGTAATATACAGGAGCCTCAACCAAAAGTCATAACTGAGGTTGAAAATCAAGTAAACAAATTACAATATGACACATCTCAACAACAGAACGCATCCGAAATTAACGAGCTAGATGTTAAGAGGGATGTAGATACTGCCATTTCAGATAATCAAATGGAATCAACTATGACATCAACATCAAAATTAGCAAATGGAATCCCTAGAAGCAAGACAACGAAGAAAGCTATTCAACAAATGCGAGCATCAAGAACAGATCTTTCACAACATCGTAGAATTCAAGATTTATACAATGCAATAAAGGGTGCTGGTGGTATGGTGGAAGCGGTGCGCGAATTGTTTGATGTGTATCAGAAGTATGTACATGGCCCCAATCACGAAACTGCTGCACAAATCGATAGGCCAGTTGTTAGAAAAACTCTCAATGTGTTGTTTAATGAAGGTAAATTGAAAGAAACAAAGGTATCCAGTAGAACTTATGGAGGCGCTTCAATGCAGAAAAGACTTATTTACGTATCAGATATTGATACACATTCGGAGGCTTTTCGCAAAAAGAAAGAAGAGTTGGGGCAAGATTTAATGAAGAGATACCAACCTAAAGCGTCTTTCAAAGAAGTAGACACCGAAAATATCGGATATATTAATTTTAGGAGGAAATATGCTGCTGACAATTTACCTGAAAATTGCACTGATCCATCGCAAAAAGAAAATATCAGAAACCTGATCTTGTCTGATGTTAAAGCAATGGCTAGGCTTCATGGTTACATGACCGGAAGGTTTGCAAAAGCACATACTTTGTACTCCCTTCTATCGCAAGAAATGCAGCAGAAGGATCAGTTATCTAGTAAAATGAGCGACGATGAGCCTATCGTATGTATGAACTACTTCAAACGTGACATACCATTGAATATGTACATGAAAGTAATTCCTTGGACAACATTCGATGAGATGGCGTTTGAATACAAGAATGATCCTTCAAAGAGTAAACTGAGGATTAAAGACCTTCCAGAGCATTTACAAGAACTTGTCAGTCTATCATTTTCAAAGAGACGTCACTTTATTACTGAATGTGTCCGTATCTTATGGCATTTGAACCTTGTCGAGCTCATGGAGGAGATCGAAGTAACTGATGAAATAGTAACTGATGTTAGTAATCTCAAGGGTGTAAGGGAGTTAAAGACACCAAACAGGCTCCTTTATTTTAGATTGAAGAAAGATGGTCTCTTTCTATCTTTTGAAGATAAACCCGTACTTATCGATGGTATGACGCTCAACACAAACGAAGATATAGATAAGTATTTTGAAAGGTTGAAAGTGCTTGCGTTGAGCAGTCAAGATGCTTCAATGACAGCAAATGAGAAATCGAAGAATCCGGATATGACGAACTTCTCACATTTTGCATCGTATATTCAAAAGCATCAAAGTTGGCAAAATGACATGATGTTATCAGCAATGCAAAAACGCCTCCTTGCTAATTATACTGGGAAGAGAAGTGACATACCACGCCCAGGTACGGAGGAGGAATACAAGGAGATTGCCTATACCTGCGGAGCACCTTTACCACTTGTGAAACGTTATATAGAAGAGGAAACTTCCAAACAAGAAAAACTTAAATTGGGAGTAACTCTTCATAGACCTAAGAAGAACAAAAACACAACGAAAAAGCTTAAAAAGTCTTCGAAGTATAGCAGGTCCATTGATGCGAAAGTAAAAAAAGCGCGTTCTCGACACATGAGAAGCGAAGAATCTGAAAAAGATAGCTTTGAGGATGCTGAAGATGCAGCAATAGTGGAGCAAATACGCGAGTTTAGAAAGAAAAACGAGAAGAAAAAGAAAAGTAAACCAACAAAAGAGAACCCCTATAGGCTTGATCCACCTCTTTCTGAACCACCTGGTAAATCACCTGAGGAGCTTATAGAAGAGCATAAGAGACTTTGTAAGTACTTTCATTTACGTACAGATGATATACTTATTAAACTGTTTAAGTCCCTGAAGGTACAAGTGCACGTCAAACGAAGAATTACAGCTATTGGAACCCTGAGACTGATGAATTATTGCGTGATTCAATTGCTATCATAAAAGCTCGTACTAGGAATCACAAGAATCCATCTTTGTCTGCAGCTTTACACGTTTTCCCGAGATTTAAGACAGGTCAGTTGAGAAATCATGAGAAGACGTTACTAGAGAATCGTGAAGAACATGTCTATATAGAGCGTTTGGCAAACGAATATGAGAGTTTATGCAAAGATCTTGTCAAAGAAAAGAAAATGGAGAAGAACAGGTCATTATCTCTTGATAAAATTGATTTCATTGAGCATTTAAAGCTAATCAGAATCTATATTGATAAAAGAGCATTGTAAGTGCTAGAGGTTTAGTTGAATGTTTTAACTAATGATTGGCAGATATATTAATACAACCTCCACAGGAAAAGGCTCGAGCATTTTGGAAGTACCTGATGATGTACAGAGCTTCTTTGATCAATACGATATTGCTGAAGAAACTCATCACGCTCAGCAATATCAAGATTTACAGTCAGTATTATTTGACACGGCTCAAACTGAAATTGCAAGAGAACATGCTTACTCGAGAATGGTACTCTTCGATTCATATGAGAACTGTATTTCAAGACCAGTGAATACAATTGATGAATTAAATATTAGGCTCAGTGAAGTTGCTATTAAAGTGAGTTTTATGCAATTAATCAATATTTTACTAACACATTTGAAGATGTCAACACATGATAGAAGTGAAGGACACATGGAAAGGTCTCAAGAATTTCTTAAGAAGATACCTGATGAAGCCTTTCAAATTTCATTCAAGAGAATTAAAGATGAAAATATCCTTTATTTACCTGCAAATAAAAGTGGTCCAACGGCTTTATATGCTTTCTCTGATATATACAAACATGATAGTCACGGACCTTGGCAAGCAGAGTTTATGCAAGACTTCTTTGATGATATAATGAGTGTTCAAAAAGGTGATGTTAATAATTGGCCAATAGTTGTTCAGGAGTCGCTTCAAGCAGCGCTTTTAAGTTGTATATCCTCAAATCAAATCAAATGCAGCATCAATATTGAAAACTTTAGTGCCAGAATTGATAGTATTGGTAGTCGTGACGTTCGTAAGTAAAATGATAACCTAATATTGCTGTGACTTACGATAATTAATTACAGAGGATGATGACTATGAAAATATTATCACATTCACTCCCAGCAATGCAGCAGGTATTCAGTTACCATCAGTACCGCAATACGTATACACACCGTCTATGGAAACTGATGAGTGTGATGACATTTTATTGGATCAATGTGGTCCGATTGGTTTACTAAAAGGTGAAAAATTGACAGACAAAGAAGTGAAAAAAATGTTGAACACGTCTTCGGCTTTTATTACAGGTTATGATGCAGCTAGGCTTGTAAAGTCAAAGTTCGAAAAGAATTGGTGTCTAGCTATAGGTGACAAATTGGTTCATCCGCGTCAATGGATTGACTTAAAAGGAAACACTGTGATAGAAATATGGAATAACGTGTTATTCGCAATAATCGGTATCCTGATGATCTACCCTGGTATTTCGATGGTAAGTCATATCCCAAAGATGGCTATCAAACTAACTCGCCATGTAGGTACAGCTAAGAAAGCGGTTCGAAAAAGCTTATGATAGGCTTGAAATTAGCGATGCACTTAATGGCTTATTGAGGAATGGGTTTGTTGAAGGAAGATCTCAAAGCATTCATGAAGGCATACAGTTACATAGCCTTGAAGATGAGAGTACTACATATTTATTCTTGAGAGAAGCATAATTTAATTAGTTGTACAATATCAATACAGTTCGAATATTTATTTAATTGTCTTCAGCGTCAATTTGATGGTAATGTTCCTCTTCAGCATCTCTACTAGCGATTTGCTGTACTCTTGTGACAAATAATTAAGCGATCGAAAGGGAAAATAAGTAGAAGACACACCTCTTAGCTAATCTATGATCTTCTTTCAGATCACGCATTGCAATGCTACTTCTACAACCACTATAGGGGCATAGCTTTGTTGAACCATTTAGATAGGCTTTAATTGATTCAAATGAGTAGGAGTGATTACATTTCGTGCTAAAGTAGATTTAATGGGTTTGCTTATCATTCAAGTTGACTTACGAAGTATATGGATTCTGGAAAAGACCGAGCGTGATAGGGTCATTGAAATTTTGACGCATACTACCTATCTCGATATCAGAGTCATCGTCATCTGAATCATCATCCTCCCTAGGTATCAGTTTCTTAATATTTGGTACAACCTCGTCAGGTTTGGATACAGCCTTTGCTTTGTTAGACAATTTACAAGACGCGTGCGCTTGAGACATACCCAAATAGCATTGTAATAAGATTGAATTTTACTGTTATTTGTATACTTTTGACGCGCAGTTTTATTATCGTATTGCGCCTGACGACTATCAAACTTCTGTTGGAAGACATTCGCTGGTTTGACCTTGATAAGCAATAAGTTGTAGGTAAAGCGCAACTAGAAGCTGCTCACAATCTTTGCGTCATTTTCTATCTCATTTACTATTTCATTAGCAGTCTCTTGCTTCATTTGAAGGCGATTTTCGAGATCTACGAGAGATCTCATATTATTATCTGCTGTAGTTTCTAGCTAGATAATTGTGAGCTGCAAAAATTAAAATAGTAGAACACAAACATGCTCCCAGCCACCATTTGGGTCATACCGTTTACAGTATTGAATCTGAGTGATGAGATCAACGACGTTCTCAGAGGCTGCGTCTATCGGCACTCTCTTCCAAAGAGAATTTAAACTTCTTATGGAATTTCTATCGTTTCTCCCGATATCTGCGTCTTTATCATAATCTACGCTCATCGTTATCACTTTTAAAGCGCTCTTCTCTCTTGAGAGTAAACGACCATCAATAAATTGAATAGAAGGAAATGTCGGTCATGAATTCCTTCTTAAGATCTTTTACTAGGTTAAACCTACGCACCCAATATCAGCAGCCATCCACTGTTTTCACTCGGCGGTCAATCTCATCAACTCCTACCAACTTTGCTACTTTAAACCAAATTATGAGAGGAGCTCGTAAATCATTCACAAAAGTTTCTAAAGCACCTGCATTAGAGAATAATCCACAAAAGAAGGGCGTTTGTACAAAGGTTTATACCACCAAACCAAAGAAACCAAATTCTGCCATTCGTAAAGTTGCTCGTGTAAAGTTATCAAATGGTAGAGTCGTTAGAGCATACATCCCAGGTGAAGGTCACAATCTGCAGGAACACAGTGTTGTTTTATTACGTGGTGGACGTACTCAAGATCTTCCTGGTGTTCGGTAGGTTGATAATATCTTCCAAAGGATAACTACTTATCATTTATTAGTTACAAAATAATAAGAGGTGCTTTAGATTTCAGTGGTGTCGTCGGTCGTTCATCTGCGAGGTCAAAATATGGGTGTAGGTGCTCCTTTCTTTGACTTACTTTATACTAATTTCAGCAGCTAAAAAACCTAAAGATTTGTAATCGCGTTAAATAGTAGATATAATGAATCATTTTTTTATAGATAAGAGCAGATAGTATTGCAAGGTCATCCAAGTTTCTTTGTTTGTAAGTAAACATCAACATCATGTCATTGCAAATGTACGTAAAGAAGTCATTAGAATCATCCAAGCACAGATTAAATATAGCTTGGATCATTTACTCGTGCTACTTCGAAGACCTATTTTTTCGCAAGTACTTGAATAGAGGGATTTCTTTGGGTCCTTTTATTGAAACTTTTAAGAAATTTAATACAGAGTTCTCTTATGAACTAAATATCGGCAGACTCTCTAGATTTGTTATTGAAATTATCCTTAATCTCATTGTTAGAATTACCCAAACAAGCAAGGCTATACAAGTTGAGAGGTTGACAAACGAAGAAAAGCGGGAATCTTTCAAACCTAATAAAATGGCCAATATCCATCGGATTGAAGCTCAACCAGCCTTTCAGCAACGCAAGTATGCTTTTTGTCAACAGTTTTTCGTTGATAACATAACTTATCCATATCCTGTCGACAAGAATAGTAAGGAGGCAATTGCTATAAAAGATGGTGTAACTATAAAACAGGTGAGTTTGGCCATCACGCATGTACAACATTAAAAATAGTCAGGTTGATCAGTGGTTCAGCAACCATCGCAAGCGATCTGGTTGGAACGACATCTGTAATATCACTGAAAGCAAAGAAAGGGCTTCCACACTTGTCAAAACAGTTCTAATAGGAAACAACGATTTTATTCTGCCACAACCGGTAAGTATGATTGTTGTTGCCTATATTCAGCGTGCAACAAATAACTCGGAAAGTATGCCTAGTGAATGGCTTGCAAAAACACTAGCTCATTTGAGCTATGATTATCCATTTCTAATGAAAAAAAGTAAAACGAACGATAAGACTCCTGAATACCTAGCGAATTTCCAAGATTCGAAGAAAAGTATTCGCTCAGTAAATAATAAATTGATTTCTGTTTAATGTAACTTGCATTGTATTACTTTTAATATTTATATTTTTCCTTAAAA

GACTGCTAAGTGAATGGAACAACGATGGGTACAACAAAAAAAGAAAAGAGAATAAGTAATTGCGAAGAGCAGAGGAAATGGGTGCTGTAGGGAGATATTACAAAGCTCTGCTGTGCGACTGAAGTGAATGGTTAGTGGAAATTTGAAATCCAGATATTAACACACTTAGAAGATTAGTTGTTATCCACTTTTGTGAGTCATATCATAGAAATAGAGATGATGAATGCAGTGTTATATATATTTTCTATTGATAAGATGAAGTTTGATTGATGTGTTTTCTTTGTTCATATGCTTCCATATCGTGCGTGTAATTTGTTATATATAAAAAAAGAATCCTTTTTTTGTGAGTAAAACTCATTTAGTTGATTGTGATGAATTATTTCTTTGATGTTGTTTATCTTTGTTTGACTTCTTTTTAGAATTATTCGACTTATTTTTACCTGTTTCGCTACCAACACTAGCAGACCTACGTTTTTCTTTATTACTCTGTTGTTGCTGTTGTTGCTTCTTCCGTGATGCTTGTAAACTCGCTGAGTAGAGGTTATTCAGGCTTGAAGAAGTCTTGAATTGTCCTTGTGAATTGTTATTACTCAATGCGGGAAAACCTTCTTCTAATCCAGTCGTCAAATTTGGTTTAAACGGTGGTGTATCCTCATCGACATTGGTTGCATCACCAGCTTTATCATCTAACAGAGGTAAAGTTGCGTTTGGAATTATTCTTGAATACGTTCGGCCACCACCTACTGACTTCTCAAACTCTCTACTTGATATTCTAGACGTTTTGTTGGATACTCTAGGAGTTGAAGGTACTGAACGTGATCGAGTAGGTAATTTACGACCCGTTGTAGTTGATGGAGTTGTTGCCGTTGTTGTTGTTGCTTTTGAAGGCGTTTGTGTATTCCTGGCAGCAGCTTGTTTCTGCAATTTAAGCCAACGTCTTGCTGACATCCTTGGTGGTGGTACACGTACATCAGCTGCCAGAGTTAATGCAGACGTAGTTAGACTTTCCGAAGCGACGAAAGTGTTCTGACCGAATGTATAACGTTGTGCTTGTGGGAATGGAGGGAATCCCAATCCTAGTCCTACTGTTTGCTCTTTCTCTTCTCCATTTCTAGAAGGTTGAGCGGTTGATGTTACTGGCGCACTCAATGTAGATGTTGTCGACAAAGGTGGTGAATTTGTAGGTGTACCAGACTCAGACGGTTGAGTGAATGTTGATGGTGTTATTCTAATTTGCTGACCAGCTCCATTTGTAACAAACGTCGGCACTCGTATTTTCATATCACTGTTATTAGCTGCTATTTGTTTTTGGATCAATTGTGAAGGTGAAGCATTCACACTATTAGCGGTTGTTGCTGCTGCAGCTACCGCTGCTGCGCTGCTCGATGCACGTGAATGTAAAGCAGCATTGGCTAATCTCGGAGAATGCCTCAATGGTGCAGGTATTGGTACTGGTGGCCATCCAATTTCAGCTTGAGTTGTTGCATCAGCGTAGGGCCCTGAAACATTAGGAATTGTAGCTTTAAGATGATGTTTTGGTGCATACTTCCTCTCATGTAATCTAGATAACCAAGTTGGTCCCACATTTGCACCTGAACTAGCTCTTCTTGGTATACTACTACTTTGTTCGGTTCTTGTGGATTCATCTGAAACTAAATTTTCTGCTTCCCAGAAAGCACTACCTAATGGTTTAGTATGTGACTGAGGATGTTGATACATCAGATGCTCTTCATGTGCGTTCCTCATCCTTTCATTTTGAGCAACATTTAATGCTATTGATAACGCTTGAGCATGTGATGATGATTGTCTACGTCTATTAAATGTAGCTGGTGGTGATGATATTTGATCGCTGACTTTTCTTGAAGGGAAGATTGAGGATTGCGATTGAGTAGGTGAATATGATTGTTGAGATGCAGTTTGAGTCATATGCGTATTGTGAGAATGCGTTTGTACAATTGGAGGTGGTTGTAAGCTAGGTGGAGGATGTAATGAAGGTCCTAGCGTCGGTCCTAAAGATGGGTTCGTGAGAGGTCTTGGTGTGCGATTTCCAGCGGAGAAAGGAGCAGGTACGAGGGCTTCTTCTCTTTCCTCACATAGATCTACGAGTACTTGTGAAGGTAAAATATCGGTTTTTCCTACAGTTTGTAACATTCTTGAAGCACGCATGAATTCGCCACGGATTGTGTATAAACCTGTGGTTGTTAGGAACGTTTCATTCAAAATTTTCTAAAACCCACCATCAGCTGTTACCGTTCTAGCTACGTTATAGGTAGTATCAAATGGATCCTCTATGCAAAGTCTATTCCTATCACGAGCGAATTCACTGGCACCATCGACTTCTAAATCTTGTACCCAACCTTTAGCGTCCTTAGAGAGAATACCACCTTCAGTTCTAATAGAGATAACATCGTGGGTATAGTTAAAATCTTTTGCGTAGAATCTAAAGAATTCCCATAATAATTCACCGACAGATTGAGTATTAGCGGATGACCATTCCTGACGAAGTAAAGCAACATCATCGAAGAAGTAAATATTTCTACCTTCATAACTTGCTGATTCTGGGCTTACTGGTCGTAATGGAGGTATTCGCTGTAAGTTTGGTAGAACGGGTGGTTGTTTAACGTGAGCCAAGAAGAAGATGACAAGAAGGGTGAATCCGTAACTACTAAGTGTTCCCCTGTATGCGCTGTTAATTTTACGCCTTTTACTCCAAACTTTTATGAAAAGGACGAGGGTTCTCAAACGAGGAGGGTCTATGGAGGCGTAACCAAGAAGTAAGCGAGTATTTTCTAATGCTAGCTGACCACCAAAGCCGATATCACATGCTATACCATACGGCAGAGCAGGAGTGGGTGCCAAGGTGAGCTTTATGATAGGAATACGAGCTTTCGGGAGTGGTTTGACGTGAAAGTTAGTTTCTATGGACTATTAAAAGCGAAATAATAAAATAAAGTGCCTACCATGTCTAATTAATTCGCCAACTATTGTCGTTAGTTCTGAGGATGACAGTGGTTCAGACGCCGGGTCTAGAATGCATTGTAGATCCTGTAAATTGTTATTAGACGAATAAATGGTCGGTAATTAATAAGATACCATATCTGTAGTCGTTAGAAAGCGTCACAACGTCCGCAAAAGACACCTACCTGAATTCCTCAAAGCGAAACCGTTTGCCATAGAACCAAAAGCTATTAGTCTTGCACCGGGACTGACTCTATCTATCAACTTCTCTAATAACAGCCTCGTCTGTTCTTTTATCGCGTACTCTTCTGCAGTCGGGAGAAGAGGCAGTATGAAACTGAATAAGCAGCCAGTTAGGTCCTGTGACAGAGCTTCCCTCATTGAGTTAGAAGCCCAATTATTCGCCAT

**Alignment of upstream regions**

```
Wm1      TACATTTTAGCTTTGTATAATAACAAGTAAGCCACACTATCTTCACCTCCACCTTCTAAT	60
Wm5      TACATTTTAGCTTTGTATAATAACAAGTAAGCCACACTATCTTCACCTCCACCTTCTAAT	60
         ************************************************************

Wm1      TGCGCAATTTTCTCCTTTGATATCTTGCTAACCTTGTTATCATCGAATTTATACCAATCA	120
Wm5      TGCGCAATTTTCTCCTTTGATATCTTGCTAACCTTGTTATCATCGAATTTATACCAATCA	120
         ************************************************************

Wm1      TCTTGGTGTTCTTCACCTTTGTGTACATCTCCCCTAACCCAACCGATGTAGTGACCACCA	180
Wm5      TCTTGGTGTTCTTCACCTTTGTGTACATCTCCCCTAACCCAACCGATGTAGTGACCACCA	180
         ************************************************************

Wm1      TCTGCGTTGACACCTTTGTGTGTAACTATGCCAACCAAATCGTACAATCCACTCTTTGAA	240
Wm5      TCTGCGTTGACACCTTTGTGTGTAACTATGCCAACCAAATCGTACAATCCACTCTTTGAA	240
         ************************************************************

Wm1      GAACCAATGTCATTCTTCAAGTCCTCATCAACTAACTCATCCAGTTCCTTCTTCTCCTTC	300
Wm5      GAACCAACGTCATTCTTCAAGTCCTCATCAACTAACTCATCCAGTTCCTTCTTCTCCTTC	300
         ******* ****************************************************

Wm1      GCTTGATATACTTCTTCACTGAGTAATTCTGCATCACCAATCTTGTTTTGTTCATCTTTT	360
Wm5      GCTTGATATACTTCTTCACTGAGTAATTCTGCATCACCAATCTTGTTTTGTTCATCTTTT	360
         ************************************************************

Wm1      ATCTCATCGATTAACATCTCATCATTGTTCTTTGAATTGTTAGCCTTCTCCTCCAAGTCG	420
Wm5      ATCTCATCGATTAACATCTCATCATTGTTCTTTGAATTGTTAGCCTTCTCCTCCAAGTCG	420
         ************************************************************

Wm1      TTTCTCGTCTTGATGCGCTTCCTCAATCTTCTTCTTTCTTCTCTATCCTTGTTGATCGAA	480
Wm5      TTTCTCGTCTTGATGCGCTTCCTCAATCTTCTTCTTTCTTCTCTATCCTTGTTGATCGAA	480
         ************************************************************

Wm1      TAAAGTTTCTTATTGACTGGATTGATCTTGGCTTTCAAATCGTCACTAACTAATTCGCTC	540
Wm5      TAAAGTTTCTTATTGACTGGATTGATCTTGGCTTTCAAATCGTCACTAACTAATTCGCTC	540
         ************************************************************

Wm1      GCATCGTACTCCATTGGGAATTTTACTTTACGGAGAATCTTGGCTTTTTTACCTAAGTCA	600
Wm5      GCATCGTACTCCATTGGGAATTTTACTTTACGGAGAATCTTGGCTTTTTTACCTAAGTCA	600
         ************************************************************

Wm1      GCTCTCCAGTAGAATCTCACCAGATGAACGGTAAGATAGCTTGGTAGACGATTTATTCTG	660
Wm5      GCTCTCCAGTAGAATCTCACCAGATGAACGGTAAGATAGCTTGGTAGACGATTTATTCTG	660
         ************************************************************

Wm1      GACTTCTGAACATATTGAGCGTTCCTGTTAAGAGTTCCAGAATATTTCTCAATTTGTTCA	720
Wm5      GACTTTTGAACATATTGAGCGTTCCTGTTAAGAGTTCCAGAATATTTCTCAATTTGTTCA	720
         ***** ******************************************************

Wm1      TCTAAGCCTTCACTCAAGCCTTGTTGGAGGTAATTGGTATTTGAAGAGATATTACAGCCC	780
Wm5      TCTAAGCCTTCACTCAAGCCTTGTTGGAGGTAATTGGTATTTGAAGAGATATTACAGCCC	780
         ************************************************************

Wm1      AGTTTGGTGAATTCCTCGATCGAATAAGTAGGTTCTTCCTCTGGCGCTTCAGGTGTCGAC	840
Wm5      AGTTTGGTGAATTCCTCGATCGAATAAGTAGGTTCTTCCTCTGGCGCTTCAGGTGTCGAC	840
         ************************************************************

Wm1      ATTTCTTTCACATAGCTACCTCTCATGTACTGATCGACAAATGACTTGCCTTCCATACCA	900
Wm5      ATTTCTTTCACATAGCTACCTCTCATGTACTGATCAACAAATGACTTGCCTTCCATACCA	900
         *********************************** ************************

Wm1      GTGGTGCCTGCTAATGAGCTGAGTATCGCACCCCAGGCTTCTTCTGCATCTTGTTGGGCA	960
Wm5      GTGGTGCCTGCTAATGAGCTGAGTATCGCACCCCAGGCTTCTTCTGCATCTTGTTGGGCA	960
         ************************************************************

Wm1      AACTGTCCGAATCTGTCTCTCTCTGTGAACTGTTGGTAGTTTGATCTTAACGTTGTCAAG	1020
Wm5      AACTGTCCGAATCTGTCTCTCTCTGTGAACTGTTGGTAGTTTGATCTTAACGTTGTCAAG	1020
         ************************************************************

Wm1      AATGAAGTTGGTGTTATTGCGTCAGTTGAGTTGTTAAGTGAGCTAAAAAGCGCTTCTAAG	1080
Wm5      AATGAAGTTGGTGTTATTGCGTCAGTTGAGTTGTTAAGTGAGCTAAAAAGCGCTTCTAAG	1080
         ************************************************************

Wm1      GATCTACTGAGACCATTGTTCTGTGAGTTGTTTAAGTTGCTTTGTAATTCCGGTATAGCT	1140
Wm5      GATCTACTGAGACCATTGTTCTGTGAGTTGTTTAAGTTGTTTTGTAATTCCGGTATAGCT	1140
         *************************************** ********************

Wm1      CTCAGAACCTGAATTGAACTGTTCAAATAACAAGTGTTACCAAGGTTTGTCAAACCAACT	1200
Wm5      CTCAGAACCTGAATTGAACTGTTCAAATAACAAGTGTTACCAAGGTTTGTCAAACCAACT	1200
         ************************************************************

Wm1      GGGTTCTTCAAGGCTTGTGACAACTGCTTATCATCGAGATCTTCCATGAATACGACCTTT	1260
Wm5      GGGTTCTTCAAGGCTTGTGACAACTGCTTATCATCGAGATCTTCCATGAATACGACCTTT	1260
         ************************************************************

Wm1      TCTGTAGGCGCCGAAGGTAATGGACCTGCTGCACCAATAACCATGAACAAATGACCTGGT	1320
Wm5      TCTGTAGGCGCCGAAGGTAATGGACCTGCTGCACCAATAACCATGAACAAATGACCTGGT	1320
         ************************************************************

Wm1      TTTATTGTTGATCCGAGCTTCTCCATATCAGTATCATCCTTTAAGATACCACCTTTTATC	1380
Wm5      TTTATTGTTGATCCGAGCTTCTCCATATCAGTATCATCCTTTAAGATACCACCTTTTATC	1380
         ************************************************************

Wm1      ATAACTTTTTGTCTAAACTGAGTGTTAAATGAGGTATACTTCACTAAGGTTCTTCTGCTT	1440
Wm5      ATAACTTTTTGTCTAAACTGAGTGTTAAATGAGGTATACTTCACTAAGGTTCTTCTACTT	1440
         ******************************************************** ***

Wm1      ACCTCTCTGGTGCCACGCCAGTTATAGCGTAGATATCATTCTTAAAATCTAAACCAGATG	1500
Wm5      ACCTCTCCGGTGCCACGCCAGTTATAGCGTAGATATCATTCTTAAAATCCAAACCAGATG	1500
         ******* ***************************************** **********

Wm1      CTTGTGTATTTAAATCTAAATCTAATTTCTTCCCTGCGTGCTTAATTGATACTGGTATTT	1560
Wm5      CTTGTGTATTTAAATCTAAATCTAATTTCTTCCCTGCGTGCTTAATTGATACTGGTATTT	1560
         ************************************************************

Wm1      TAGTCATCTCTTCGTAGTAGTTAATAGAAAAATGACGCGTTCATTTAACTGTCAAATTAT	1620
Wm5      TAGTCATCTCTTCGTAGTAGTTAATAGAAAAATGACGCGTTCATTTAACTGTCAAATTAT	1620
         ************************************************************

Wm1      GTATTTTTCTGTGTAAAAACAATAATATGGATTTATTTATGCTATATTTAAAATGTAAAA	1680
Wm5      GTATTTTTCTGTGTAAAAATAATAATATGGATTTATTTATGCTATATTTAAAATGTAAAA	1680
         ******************* ****************************************

Wm1      ATCAAAATATTCTAATCATCTCTAGATTAAAGACTCATTGAAATACTGACGTCAACATTT	1740
Wm5      ATCAGAATATTCTAATCATCTCTAGATTAAAGACTCATTGAAATACTGACGTCAACATTT	1740
         **** *******************************************************

Wm1      CGGTTGAACTCCTCTCTTCTACGAACGTCGATTATCAAAAATAAAAAACTAGAGAAGCAA	1800
Wm5      CGTTTGAACTCCTCTCTTCTACGAACGTCGATTATCAAAAATAAAAAACTAGAGAAGCAA	1800
         ** *********************************************************

Wm1      AATGACAGTGAGTACTATTTTATAGCGTTATACTATTTTACTAAGACTCACTCTTTAGCT	1860
Wm5      AATGACAGTGAGTACTATTTTATAGCGTTATACTATTTTACTAACACTCACTCTTTAGCT	1860
         ******************************************** ***************

Wm1      TTATTACAGCTTAGTGTGGGTAATCAACTATCAATTAGAGATAGATAATTAACAGTTTAA	1920
Wm5      TTATTACAGCTTAGTGTGGGTAATCAACTATCAATTAGAGATAGATAATTAACAGTTTAA	1920
         ************************************************************

Wm1      ACAGTTTCGGTTTACTCTCTGCTGAGTACGCTTGATTCGTCATAATATCTATCAACTCAT	1980
Wm5      ACAGTTTCGGTTTACTCTCTGCTGAGTACGCTTAATTCGTCATAATATCTATCAACTCAT	1980
         ********************************* **************************

Wm1      CACACTCATAGAATCGTCACTTTCTTGGTATTAGTAGCACCAATGCCATTTGCGTTTAAG	2040
Wm5      CACACTCATAGAATCGTCACTTTCTTGGTATTAGTAGCACCAATGCCATTTGCGTTTAAG	2040
         ************************************************************

Wm1      CAAAAGTTCTTTAAATTTTTAAGCACAAACCCATTAGTTGCTAAATTACAATACTCCTTG	2100
Wm5      CAAAAGTTCTTTAAATTTTTAAGCACAAACCCATTAGTTGCTAAATTACAATACTCCTTG	2100
         ************************************************************

Wm1      AAGATCTCTCTCATATTCACTTCCATTCTCTTCTTTGATGCTGTACAAAGGTGAGTTTAA	2160
Wm5      AAGATTTCTCTCATATTCACTTCCATTCTCTTCTTTGATGCTGTACAAAGGTGAGTTTAA	2160
         ***** ******************************************************

Wm1      GTGTAATAATCAGAAATATCATTACTTACATTATCAATAGAATGCTTAGAGTCGTTAAAG	2220
Wm5      GTGTAATAATCAGAAATATCATTACTTACATTATCAATAGAATGTTTAGAGTCGTTAAAG	2220
         ******************************************** ***************

Wm1      AAGGTCAAACAGCCAAAGAGGAACATGCTTTCACAGATGTACGCTCAGAAACTAACTTTG	2280
Wm5      AAGGTCAAACAGCCAAAGAGGAACATGCTTTCACAGATGTACGCTCAGAAACTAACTTTG	2280
         ************************************************************

Wm1      CCGCACGCAAGTACGTCACTTTTATATTCTAAAGATCTAAATTATTCACACACTTTTTAG	2340
Wm5      CAGCACGCAAGTACGTCACTTTTATATTCTAAAGATCTAAATTATTCACACACTTTTTAG	2340
         * **********************************************************

Wm1      GTTTTACTCACAACGTAACGTTTATCTCACCGGTATGTCTCACCTTTCTTTTCGTCATCT	2400
Wm5      GTTTTACTCACAACGTAACGTTTATCTCACCGGTATGTCTCACCTTTCTTTTCGTCATCT	2400
         ************************************************************

Wm1      TTACAAAACTCATTTTCAAAAGGTTTCACCCTCTTTCTCAGCTTGTGGGTATTACTGCTA	2460
Wm5      TTACAAAACTCATTTTCAAAAGGTTTCACCCTCTTTCTCAGCTTGTGGGTATTACTGCTA	2460
         ************************************************************

Wm1      CGTTTTT-AAATTAACCAACTAATTCATTCCTTCTTCCATTAGAATTCTTTCACGCGTAT	2519
Wm5      CGTATTTTAAATTATCCAACTAATTCATTCCTTCTTCCATTAGAATTCTTTCACGCGTAT	2520
         *** *** ****** *********************************************

Wm1      TTGGTATTATCATGGACCTTATTCAATCACAAGAAGAATTGGGTTTGATGAAGGTACTCA	2579
Wm5      TTGGTATTATCATGGACCTTATTCAATCACAAGAAGAATTGGGTTTGATGAAGGTATTCA	2580
         ******************************************************** ***

Wm1      TTATACAATTATATTTAAACAATTTACTTACATTCTTCATAGAAAGATGGCCAATCATCA	2639
Wm5      TTATACAATTATATTTAAACAATTTACTTACATTCTTCATAGAAAGATGGCCAATCATCA	2640
         ************************************************************

Wm1      GCTGCTCAAAACTTCAAGAAGCAAATTGAAGCTAAAGATCGTGAAATTGGTTAGTATTAT	2699
Wm5      GCTGCTCAAAACTTCAAGAAGCAAATTGAAGCTAAAGATCGTGAAATTGGTTAGTATTAT	2700
         ************************************************************

Wm1      TAATAAAACCTTTCACCTTATTTACAAGCTTTTCCAGAAATTCTCAAAAAGCAATCTACA	2759
Wm5      TAACAAAACCTTTCAACTTATTTACAAGCTT-TCCAGAAATTCTCAAAAAGCAATCTACA	2759
         *** *********** *************** ****************************

Wm1      CAAAACAACAAAGCTATGAACGAACTCGTTGATAACGCTAATTCTTCATCAGGCTCAAAG	2819
Wm5      CAAAACAACAAAGCTATGAACGAACTCGTTGATAACGCTAATTCTTCATCAGGCTCAAAG	2819
         ************************************************************

Wm1      AAGGAGAAGTAA	2831
Wm5      AAGGAGAAGTAA	2831
         ************
```

**Alignment of mating loci** (with complementary strand of Wi5)

```
Wm1      TGCAATTGTACAAGATGTATGTTGTTGATGACAGTAAATAATTCATAGTATGTTTGGTTG	60
Wm5      ------------------------------------------------------------	0


Wm1      ATGTTATAATTGTTCTACTTTATAGATTATTACGATAGTTTTGTAGATTAAAAACAACAT	120
Wm5      ------------------------------------------------------------	0


Wm1      TTGGGTCAACATAGTTAGAGTGATTCAGATTATCAATAAAAACCTTATCTTGAATAGTAT	180
Wm5      ------------------------------------------------------------	0


Wm1      ATTCAGAAGAAGTATTCAACAATTCTGCTGTTAGTATCTTGTTTGGTAATGGGTCAACTA	240
Wm5      ------------------------------------------------------------	0


Wm1      CATCAAGAAGAGGCAATTGCAAATGTAAATATCCTTGTGAAAGAAGTAAGATTTTCAGTT	300
Wm5      ------------------------------------------------------------	0


Wm1      GCTCTAAAGTAGTATTCATAGTGATAATTTCGCAATTAACTTGACTTACCTCGTAACATT	360
Wm5      ------------------------------------------------------------	0


Wm1      TTATTCTCGTTTTCAAGCCTAGTGACCAGGTCTTTTTGGTTTTGACGATAACGTCGCCCA	420
Wm5      ------------------------------------------------------------	0


Wm1      ATCTCTAGATGATATTAAGCTACTGTAGGTAATCTGTGGCTTCATGAACTAACCTTGGCT	480
Wm5      ------------------------------------------------------------	0


Wm1      TCTTTCAAATCGTTAGGAAAGCTCTGACTTTTTAAAAAGACTTACTTCGTAGCTGGACTT	540
Wm5      ------------------------------------------------------------	0


Wm1      TGAGTGTTGCGAGGTCGACCACGACCTCTCGATACTATTGTTTTACTAGACATCATTAAC	600
Wm5      ------------------------------------------------------------	0


Wm1      TACCTTAGTTTCTATCAGAAAGTATATGTGATGATTGATATATCGAATATATGTACGCTC	660
Wm5      ------------------------------------------------------------	0


Wm1      TGATCTGTCCGTCCGCATTCCTATCTGTACCTTTTTTTAAGACAATGAAACTAAAAAAAA	720
Wm5      ------------------------------------------------------------	0


Wm1      AATCATGCGCTAAACAAAGATACCAGAAATAAAGTTACCATTGTTCTAAAAACTGACAAT	780
Wm5      ------------------------------------------------------------	0


Wm1      AACAATGGCGATAAATTTTTCAATGGTTAGCATTATAAATAAGCGTCTTTCTCTAATAAG	840
Wm5      ------------------------------------------------------------	0


Wm1      CTTTATTGACATTAAAAGATGGAACCAGAGTCATTTAACAACTTCAACTGTCAAGATACA	900
Wm5      ------------------------------------------------------------	0


Wm1      GACAAGTTCTATTCACAAAATGATGAAACCTACTTCGAAACTGGTATTGGAAGGACTGAA	960
Wm5      ------------------------------------------------------------	0


Wm1      ATGTCAAGTGAAGATTTAGCTAATTTCGATAATCCTATGGATGATGACACTGGTACAGTA	1020
Wm5      ------------------------------------------------------------	0


Wm1      GGTGATAAGGCTGACACCATATACTGTGTCATTGCTTAAGTTAGTTATTATCATTCAAAA	1080
Wm5      --------------------------------TTTTAAGGAAAAATATAAATATTAAAAG	28
                                         *  * * *  *  *** *  *** ***

Wm1      TTTATCATTACTAATT-CATTAAACAGAAAATTCAGAATTTTGTGCAATGATTGTTCTTC	1139
Wm5      TAATACAATGCAAGTTACATTAAACAGAAATCAATTTATTATTTACTGAGCGAATACTTT	88
         *    ** * * * ** *************       *** * * *   *    * ***

Wm1      AATATATATATATACATTATGCAAATAATTCAGGTGCTTGACTGTTTACATCGACGGCCA	1199
Wm5      TCTTCGAATCTTGGAAATTCGCTAGGTATTCAGGAGTCTTATCGTT--------------	134
           *    ** *    * *  ** *   ******* *  * *  ***

Wm1      CATTCTCTACTTTAGATCTCCATTCGTTTAGACGTGAAGCCAATGATTTGACTTGATTTT	1259
Wm5      ------------------------------------------------------------	134


Wm1      TATCGAGTACTCTTGGTTGTACCCAATTGATGCTAGCTGAGCTTGAGACTTGGTCAATAG	1319
Wm5      ---------------------------------------CGTTTTACTTTTTTTCATTAG	155
                                                 * ** *   **  *** ***

Wm1      TACCACGGATAAGTTTAAGCCTTATTATTTTAAGTTGAAACAATCCAGTAAATAGATTAC	1379
Wm5      AA--ATGGATAATCATAGCTCAAATGAGCTAGTGTTTTTGCAAGCCATTCACTAG-----	208
          *  * ******   **   *  ** *  *   ***    *** *** * * ***

Wm1      TCACGATAATGCTTTCATAATTAAGTGTTCTACTTCATCAAGTGGAAGCTTAGTTTCAGC	1439
Wm5      -----------------------------------------------GCATACTTTCCGA	221
                                                        ** ** **** *

Wm1      GCCGATCGTTGAGAAAGACATGTCACGGTCTTTTGATGACCTTTTGAACACGCATTCGAT	1499
Wm5      GTTATTTGTTG------------CACGCTGAATATAGGCAACAACAATCATACTTACCGG	269
         *    * ****            **** *   *  * *        * **  * * *

Wm1      TAATGCCATAAGGCATATCTTTTGACGAAGGAAAGCTTGACTGTCGTGTAAGATTGGCTT	1559
Wm5      TTGTGGCAGAATAAAATCGTTGTTTCCTATTAGAACTGTTTTGACAAGTGTGGAAGCCCT	329
         *  ** ** **   *    ** *  *  *  * * **    ** *  **  *   * * *

Wm1      ATATAATGTTACTTTCAGTGTTCT--AAAATGCTATTACACCCACTTCATTTGCCAATTT	1617
Wm5      TTCT----TTGCTTTCAGTGATATTACAGATGTCGTTCCAACCAGATCGCTTGCGATGGT	385
          * *    ** ********* * *   * ***   ** ** ***  **  **** *   *

Wm1      AGAGGCATTAACCTCAAACTTGGTTATATCA-----------------------------	1648
Wm5      TGCTGAACCACTGATCAACCTGACTATTTTTAATGTTGTACATGCGTGATGGCCAAACTC	445
          *  * *  *      *** **  *** *

Wm1      --------------------------------------------------CCTTCGTTGA	1658
Wm5      ACCTGTTTTATAGTTACACCATCTTTTATAGCAATTGCCTCCTTACTATTCTTGTCGACA	505
                                                           * *      *

Wm1      AAATGTAAATAATATCTTTCAACCATTCTAATTCGGTTCCATCAAGAGCATGTAAGATTG	1718
Wm5      GGATATGGATAAGTTATGTTATCAACGAAAAACTGTTGACAAAAAGCATACTTGCGTTGC	565
           ** *  ****  * * * * * *    **   * *  **  ***   *  *  * *

Wm1      GATGAGTCAACTGAATTTTTAATTAGAGAACTACTATATATTATATTAAAGTGAGTAAGC	1778
Wm5      TGAAAGGCTGGTTGAGCTTCAATCCGATGGATATTGGCCATTTTATT-AGGTTTGAAAGA	624
             ** *   *  *  ** ***  **    ** *    *** **** * **  * ***

Wm1      TTACCAATTCACCGAAATTGTAAATTGTATCACCTAATAAGGCAGCGATGGCTAAATCGT	1838
Wm5      TTCCCGCTTTTCTTCGTTTGTCAA------------------------------------	648
         ** **  **  *     **** **

Wm1      GACTTCTTTGGATTTGCTCAGCTTTGTCAAGTTGGTCCATACCAGCACATGAGATATACA	1898
Wm5      ----CCTCTCAACTTGTATAGCCTTGCTTGTTTGGGTAATTCTAACA-ATGAGATTAAGG	703
              ** *  * ***   *** ***     ****   ** * * ** *******  *

Wm1      GTAATGTTGTACGGTAAAATGCTGCATATTCCGCTTTTGACTGATTTTTGA-----TTAG	1953
Wm5      ATAATTTCAATAACAAATCTAGAGAGTCTGCCGATATTTAGTTCATAAGAGAACTCTGTA	763
          **** *        **  *   *  * * *** * ** * *   *          *

Wm1      GTCGATTTCTGAAGAGTTCCAAATACTCACTTTAAAGTAATCACCACTAACGCGTAGCCA	2013
Wm5      TTAAATTTCTTAAAAGTTTCAATAAAAGGACCCAAAGAAATCCC----------------	807
          *  ****** ** **** ***  *        **** **** *

Wm1      AGCACCAGTGACACTGGCTTCTACGCCATCAATCGAATCTAAAACACGCTCGCAAACATC	2073
Wm5      ------------------------------------------------------------	807


Wm1      CATAGCTTCTTTAGCTTTGTCTAAGTTACCAAGTAAAAGGTCATAATGTGCTATTGAGAT	2133
Wm5      ------------------------------------------------------------	807


Wm1      TTTTGCTAAAACGTAAGCCTCAGATTCGGTTTTAGTTGGTGGTTGCTCCTCATCGAACGA	2193
Wm5      ------------------------------------------------------------	807


Wm1      TTTTGTATTGGTTGGCTTCAGTGCAGGTGGTAATGCAGCTCTTGGAACTGGTAGGGTGTC	2253
Wm5      -----------------TCTATTCAAGTACTTGCGAAAAAATAGGTCTTCGAAGTAGCAC	850
                          **  * ** **  *   * *    * **   * * **     *

Wm1      CAATAAAGATTCTAAGAAGTTTAGAGACTTTTGTGGAATCATCTCCTGTGCTACTCTAAC	2313
Wm5      GAGTAAATGATCCAAGCTATATTTAATCTGTGCTTGGATGATTCTAATGACTTCTTTACG	910
          * ****   ** ***   * *  *  ** *  * * ** **        ** ** **

Wm1      GCCAATTTCCACTAAACGTAGTTGATTCAACTTATCCTTGAATGTTGAGATAAAGTTATT	2373
Wm5      TACATTTGCAATGACATGATGTTGAT----------------------------------	936
           ** ** * *  * * *  ******

Wm1      CCACAAGTCAACCAAATACTTAGAAATATCATCCTTCTGTATGATTTCTTGTAGTGTTTT	2433
Wm5      ------------------------------------------------------------	936


Wm1      AGTAAGCTGATGGTATAAACTTTGGTGGCTGGTTAGCAATTATTTGATTCCTCGCGTAAG	2493
Wm5      ------------------------------------------------------------	936


Wm1      ACGCACCGCTTGCTATAATTAATTTCTAATTTAGTCAAATCTTCTGTTAATGAAGCTGGC	2553
Wm5      ------------------------------------------------------------	936


Wm1      GCCTCTGCTTTTACTTTATTTATGAATTCTGTCGGACTCATGTTGCGCGTGCGTAATCGT	2613
Wm5      ------------------------------------------------------------	936


Wm1      TCTACTCAGGGCTCTGACCAACCCATTCACTGACGCAACGAAGCAATTCGATTTTTTATT	2673
Wm5      --------GTTTACTTACAAACAAAGAAACTTGGATGACCTTGCAATACTATCTGCTCTT	988
                 *    ** ** ***  *   ***      **   ***** * ** *  * **

Wm1      TACTCTCTTCACAAAGAACTTTCAACTATTAGCAAATTAAAATAAATGTCATTCGCAGAT	2733
Wm5      ATCTATAAAAA-------------------------------------------------	999
           ** *    *

Wm1      ATAAGTCAGATTGGTGATGGGCTTATTCATACCATCAATCAAAGCCTTCACAATCATCAA	2793
Wm5      ------------------------------------------------------------	999


Wm1      AGTAAACAAAGAGATGAAGCCAGTATACAGAATTATCCCGTCATACAGCTGCTCGACCAC	2853
Wm5      ------------------------------------------------------------	999


Wm1      TTAAATGGCAAAAAACCTGTACATAGAGATAATGCTGTGATTGGTATTATAAAGAGTCTG	2913
Wm5      ------------------------------------------------------------	999


Wm1      CACAGTTTGGAGAACAATGGAATGCCTAAAAGCTGTATGGACGAAGTCAAGGAGGCGCTG	2973
Wm5      ------------------------------------------------------------	999


Wm1      AAGTATCAAGATTTAGTTAATGGATCATTTATTGCGTCTACGTGCAAATGGGCTGAAAAC	3033
Wm5      ------------------------------------------------------------	999


Wm1      GGTGAAACTCTTGAACATAACATTGATGATAAGCATCGAAAGCAGGTATGTTACATAGTT	3093
Wm5      ------------------------------------------------------------	999


Wm1      TCTCAAGATAGAGGTTAATAACAAATGTAGGTCTTTCGCGAAATCTTGCCAGAATTGTAC	3153
Wm5      ------------------------------------------------------------	999


Wm1      AAACAGAATCCTAGACCTTCAGTTGCTGACAGAAATTATTTGAGTCGAGTATGTTACCTT	3213
Wm5      ------------------------------------------------------------	999


Wm1      AGGGCCCGTGTTTCAAGCTAATATAAAGCTTAAGGTCACCGGTTTGGGTCGCAAATGCCT	3273
Wm5      ------------------------------------------------------------	999


Wm1      TGACACATTCGTTAGTTCTTTACATTTATGTCGTATACTAATTCAAAGTTTAATAGTTTC	3333
Wm5      ------------------------------------------------------------	999


Wm1      AAAATAAGAGAAACCGTCACGGTCAAACCAGAAAAAGAGTGTCATCTGTGCATTCATCAA	3393
Wm5      ------------------------------------------------------------	999


Wm1      ATAGTGCAACTTCTCAAGTTGATATATTGTATGATGCGTCAGACAACGGGTAAGTATTGA	3453
Wm5      ------------------------------------------------------------	999


Wm1      TATCTATAATTTCCAGATGGATAACTTTAGATATACAGCAAAAAGAGGAAGTATGATAGT	3513
Wm5      ------------------------------------------------------------	999


Wm1      ATGAATGAAGTAACCAATACTTGCAATGTGAATCAAAGTAACACGGTTCTCAATTCAAAC	3573
Wm5      ------------------------------------------------------------	999


Wm1      AAGGTAGTATTTCCAGTTCATGAAAATGCAGAATATAGTGCTTCAAGCCTTTCTGGCACT	3633
Wm5      ------------------------------------------------------------	999


Wm1      CTTGAAGACGATATTCTTTCACAATTTCAATGCTCAAATGAATACATCAGGCCAAGCTCA	3693
Wm5      ------------------------------------------------------------	999


Wm1      ATGGGCGAACAATTCAATGACACTAAATACTTACTGGATTTTCCGGAAGTGGTTAACATA	3753
Wm5      ------------------------------------------------------------	999


Wm1      TTTAATAATACTGATGATCTTTTCCATGAGTCATTATTTAGCATGTCACTATACCCAAAG	3813
Wm5      ------------------------------------------------------------	999


Wm1      ACGGACATGGAACACATACTACTAAATGTTTAAGACACATAACTAAATTTCCCTTTATAC	3873
Wm5      ------------------------------------------------------------	999


Wm1      TGATATCGATACCTATTGTCGAAGATGCATTTTATCTATTTATTTAGCACAATTACAAAT	3933
Wm5      --------------------AATGATTCATTATATCTA-CTATTTAACGCGATTACAAAT	1038
                              * *** **** ******  ****** * * *********

Wm1      CTTTAGGTTTTTTAGCTGTTGAAATTAGTATAAAGTAAATCAAAGGAAAGAGCACCTACA	3993
Wm5      CTTTAGGTTTTTTAGCTGCTGAAATTAGTATAAAGTAAGTCAAAGAAAGGAGCACCTACA	1098
         ****************** ******************* ****** ** ***********

Wm1      CCCATATTTTGACCTCGCAGATGAACGACCGACGACACCACTGAAATCTAAAGCACCTCT	4053
Wm5      CCCATATTTTGACCTCGCAGATGAACGACCGACGACACCACTGAAATCTAAAGCACCTCT	1158
         ************************************************************

Wm1      TATTATTTTGTAACTAATAAATGATAAGTAGTTATCCTTTGGAAGATATTATCAACCTAC	4113
Wm5      TATTATTTTGTAACTAATAAATGATAAGTAGTTATCCTTTGGAAGATATTATCAACCTAC	1218
         ************************************************************

Wm1      CGAACACCAGGAAGATCTTGAGTACGTCCACCACGTAATAAAACAACACTGTGTTCCTGC	4173
Wm5      CGAACACCAGGAAGATCTTGAGTACGTCCACCACGTAATAAAACAACACTGTGTTCCTGC	1278
         ************************************************************

Wm1      AGATTGTGACCTTCACCTGGGATGTATGCTCTAACGACTCTACCATTTGATAACTTTACA	4233
Wm5      AGATTGTGACCTTCACCTGGGATGTATGCTCTAACGACTCTACCATTTGATAACTTTACA	1338
         ************************************************************

Wm1      CGAGCAACTTTACGAATGGCAGAATTTGGTTTCTTTGGTTTGGTGGTATAAACCTTTGTA	4293
Wm5      CGAGCAACTTTACGAATGGCAGAATTTGGTTTCTTTGGTTTGGTGGTATAAACCTTTGTA	1398
         ************************************************************

Wm1      CAAACGCCCTTCTTTTGTGGATTATTCTCTAATGCAGGCGCTTTAGAAACTTTTGTGAAT	4353
Wm5      CAAACGCCCTTCTTTTGTGGATTATTCTCTAATGCAGGTGCTTTAGAAACTTTTGTGAAT	1458
         ************************************** *********************

Wm1      GATTTACGAGCTCCTCTCATAATTTGGTTTAAAGTAGCAAAGTTGGTAGGAGTTGATGAG	4413
Wm5      GATTTACGAGCTCCTCTCATAATTTGGTTTAAAGTAGCAAAGTTGGTAGGAGTTGATGAG	1518
         ************************************************************

Wm1      ATTGACCGCCGAGTGAAAACAGTGGATGGCTGCTGATATTGGGTGCGTAGGTTTAACCTA	4473
Wm5      ATTGACCGCCGAGTGAAAACAGTGGATGGCTGCTGATATTGGGTGCGTAGGTTTAACCTA	1578
         ************************************************************

Wm1      GTAAAAGATCTTAAGAAAGAATTCATGACCGACATTTCCTTCTATTCAATTTATTGATGG	4533
Wm5      GTAAAAGATCTTAAGAAGGAATTCATGACCGACATTTCCTTCTATTCAATTTATTGATGG	1638
         ***************** ******************************************

Wm1      TCGTTTACTTTCAAGAGAGAAGAGCGCTTTAAAAGTGATAACGATGAGCGTAGATTATGA	4593
Wm5      TCGTTTACTCTCAAGAGAGAAGAGCGCTTTAAAAGTGATAACGATGAGCGTAGATTATGA	1698
         ********* **************************************************

Wm1      TAAAGACGCAGATATCGGGAGAAACGATAGAAATTCTATAAGAAGTTTAAATTCTCTTTG	4653
Wm5      TAAAGACGCAGATATCGGGAGAAACGATAGAAATTCCATAAGAAGTTTAAATTCTCTTTG	1758
         ************************************ ***********************

Wm1      GAAGAGAGTGCCGATAGACGCAGCCTCTGAGAACGTCGTTGATCTCATCACTCAGATTCA	4713
Wm5      GAAGAGAGTGCCGATAGACGCAGCCTCTGAGAACGTCGTTGATCTCATCACTCAGATTCA	1818
         ************************************************************

Wm1      ATACTGTAAACGGTATGACCCAAATGGTGGCTGGGAGCATGTTTGTGTTCTACTATTTTA	4773
Wm5      ATACTGTAAACGGTATGACCCAAATGGTGGCTGGGAGCATGTTTGTGTTCTACTATTTTA	1878
         ************************************************************

Wm1      A-ATTTGCAGCTCACAATTATCTAGCTAGAAACTACAGCAGATAATAATATGAGATTTCT	4832
Wm5      ATTTTTGCAGCTCACAATTATCTAGCTAGAAACTACAGCAGATAATAATATGAGATCTCT	1938
         *  ***************************************************** ***

Wm1      CGTAGATCTCGAAAATCGCCTTCAAATGAAGCAAGAGACTGCTAATGAAATAGTAAATGA	4892
Wm5      CGTAGATCTCGAAAATCGCCTTCAAATGAAGCAAGAGACTGCTAATGAAATAGTAAATGA	1998
         ************************************************************

Wm1      GATAGAAAATGACGCAAAGATTGTGAGCAGCCTATAGTTGCGCTTTACCTACAACTTATT	4952
Wm5      GATAGAAAATGACGCAAAGATTGTGAGCAGCTTCTAGTTGCGCTTTACCTACAACTTATT	2058
         ******************************* * **************************

Wm1      GCTTATCAAGGTCAAACCAGCGAATGTCTTCCAACAGAAGTTTGATAGTCGTCAGGCGCA	5012
Wm5      GCTTATCAAGGTCAAACCAGCGAATGTCTTCCAACAGAAGTTTGATAGTCGTCAGGCGCA	2118
         ************************************************************

Wm1      ATACGATAATAAAACTGCGCGTCAAAAGTATTCAAATAACAGCAAAATTCAATCTTATTA	5072
Wm5      ATACGATAATAAAACTGCGCGTCAAAAGTATACAAATAACAGTAAAATTCAATCTTATTA	2178
         ******************************* ********** *****************

Wm1      CAATGCTATTTGGGTATGTCTCAAGCGCACGCGTCTTGTAAATTGTCTAACAAAGCAAAG	5132
Wm5      CAATGCTATTTGGGTATGTCTCAAGCGCACGCGTCTTGTAAATTGTCTAACAAAGCAAAG	2238
         ************************************************************

Wm1      GCTGTATCCAAACCTGACGAGGTTGTACCAAATATTAAGAAACTGATACCTAGGGAGGAC	5192
Wm5      GCTGTATCCAAACCTGACGAGGTTGTACCAAATATTAAGAAACTGATACCTAGGGAGGAT	2298
         ***********************************************************

Wm1      GATGATTCAGATGACGATGACTCTGATATCGAGATAGGTAGTATGCGTCAAAATTTCAAT	5252
Wm5      GATGATTCAGATGACGATGACTCTGATATCGAGATAGGTAGTATGCGTCAAAATTTCAAT	2358
         ************************************************************

Wm1      GACCCTATCACGCTCGGTCTTTTCCAGAATCCATATACTTCGTAAGTCAACTTGAATGAT	5312
Wm5      GACCCTATCACGCTCGGTCTTTTCCAGAATCCATATACTTCGTAAGTCAACTTGAATGAT	2418
         ************************************************************

Wm1      AAGCAAACCCATTAAATATACTTTAGCACCAAATGTAATCACTCCTACTCATTTGAATCA	5372
Wm5      AAGCAAACCCATTAAATCTACTTTAGCACGAAATGTAATCACTCCTACTCATTTGAATCA	2478
         ***************** *********** ******************************

Wm1      ATTAAAGCCTATCTAAATGGTTCAACAAAGCTATGCCCCTATAGTGGTTGTAGAAGTAGC	5432
Wm5      ATTAAAGCCTATCTAAATGGTTCAACAAAGCTATGCCCCTATAGTGGTTGTAGAAGTAGC	2538
         ************************************************************

Wm1      ATTGCAATGCGTGATCTGAAAGAAGATCATAGATTAGCTAAGAGGTGTGTCTTCTACTTG	5492
Wm5      ATTGCAATGCGTGATCTGAAAGAAGATCATAGATTAGCTAAGAGGTGTGTCTTCTACTTA	2598
         ***********************************************************

Wm1      TTTTCCCTTTCGGTCGCTTAATTCTCTGTCACAAGAGTACAGCAAATCGCTAGTAGAGAT	5552
Wm5      TTTTCCCTTTCGATCGCTTAATTATTTGTCACAAGAGTACAGCAAATCGCTAGTAGAGAT	2658
         ************ ********** * **********************************

Wm1      GCTGAAGAGGAACATTACCATCAAATTGACGCTGAAGACAATTAAATAAATATTCGAACT	5612
Wm5      GCTGAAGAGGAACATTACCATCAAATTGACGCTGAAGACAATTAAATAAATATTCGAACT	2718
         ************************************************************

Wm1      GTATTGATATTGTACAACTAATTAAATTATGCTTCTCTCAAGAATAAATATGTAGTACTC	5672
Wm5      GTATTGATATTGTACAACTAATTAAATTATGCTTCTCTCAAGAATAAATATGTAGTACTC	2778
         ************************************************************

Wm1      TCATCTTCAAGGCTATGTAATTGTATGCCTTCATGAACGCTTTGAGATCTTCCTTCAACA	5732
Wm5      TCATCTTCAAGGCTATGTAACTGTATGCCTTCATGAATGCTTTGAGATCTTCCTTCAACA	2838
         ******************** **************** **********************

Wm1      AACCCATTCCTCAATAAGCCATTAAGTGCATCGCTAATTTCAAGCCTATCATAAGCTTTT	5792
Wm5      AACCCATTCCTCAATAAGCCATTAAGTGCATCGCTAATTTCAAGCCTATCATAAGCTTTT	2898
         ************************************************************

Wm1      TCGAACCGCTTTCTTAGCTGTACCTACATGGCGAGTTAGTTTGATAGCCATCTTTGGGAT	5852
Wm5      TCGAACCGCTTTCTTAGCTGTACCTACATGGCGAGTTAGTTTGATAGCCATCTTTGGGAT	2958
         ************************************************************

Wm1      ATGACTTACCATCGAAATACCAGGGTAGATCATCAGGATACCGATTATTGCGAATAACAC	5912
Wm5      ATGACTTACCATCGAAATACCAGGGTAGATCATCAGGATACCGATTATTGCGAATAACAC	3018
         ************************************************************

Wm1      GTTATTCCATATTTCTATCACAGTATTTCCTTTTAAGTCAATCCATTGACGCGGATGAAC	5972
Wm5      GTTATTCCATATTTCTATCACAGTGTTTCCTTTTAAGTCAATCCATTGACGCGGATGAAC	3078
         ************************ ***********************************

Wm1      CAATTTGTCACCTATAGCTAGACACCAATTCTTTTCGAACTTTGACTTTACAAGCCTAGC	6032
Wm5      CAATTTGTCACCTATAGCTAGACACCAATTCTTTTCGAACTTTGACTTTACAAGCCTAGC	3138
         ************************************************************

Wm1      TGCATCATAACCTGTAATAAAAGCCGAAGACGTGTTCAACATTTTTTTCACTTCTTTGTC	6092
Wm5      TGCATCATAACCTGTAATAAAAGCCGAAGACGTGTTCAACATTTTTTTCACTTCTTTGTC	3198
         ************************************************************

Wm1      TGTCAATTTTTCACCTTTCAGTAAACCAATCGGACCACATTGATCCAATAAAATGTCATC	6152
Wm5      TGTCAATTTTTCACCTTTTAGTAAACCAATCGGACCACATTGATCCAATAAAATGTCATC	3258
         ****************** *****************************************

Wm1      ACACTCATCAGTTTCCATAGACGGTGTGTATACGTATTGCGGTACTGATGGTAACTGAAT	6212
Wm5      ACACTCATCAGTTTCCATAGACGGTGTGTATACGTATTGCGGTACTGATGGTAACTGAAT	3318
         ************************************************************

Wm1      ACCTGCTGCATTGCTGGGAGTGAATGTGATAATATTTTCATAGTCATCATCCTCTGTAAT	6272
Wm5      ACCTGCTGCATTGCTGGGAGTGAATGTGATAATATTTTCATAGTCATCATCCTCTGTAAT	3378
         ************************************************************

Wm1      TAATT-TCGTAAGTCACAGCAATATTAGGTTATTATTTTACTTACGAACGTCACGACTAC	6331
Wm5      TAATTATCGTAAGTCACAGCAATATTAGGTTATCATTTTACTTACGAACGTCACGACTAC	3438
         ***** *************************** **************************

Wm1      CAATACTATCAATTCTGGCACTAAAGTTTTCAATATTGATGCTGCATTTGATTTGATTTG	6391
Wm5      CAATACTATCAATTCTGGCACTAAAGTTTTCAATATTGATGCTGCATTTGATTTGATTTG	3498
         ************************************************************

Wm1      AGGATATACAACTTAAAAGCGCTGCTTGAAGCGACTCCTGAACAACTATTGGCCAATTAT	6451
Wm5      AGGATATACAACTTAAAAGCGCTGCTTGAAGCGACTCCTGAACAACTATTGGCCAATTAT	3558
         ************************************************************

Wm1      TAACATCACCTTTTTGAACACTCATTATATCATCAAAGAAGTCTTGCATAAACTCTGCTT	6511
Wm5      TAACATCACCTTTTTGAACACTCATTATATCATCAAAGAAGTCTTGCATAAACTCTGCTT	3618
         ************************************************************

Wm1      GCCAAGGTCCGTGACTATCATGTTTGTATATATCAGAGAAAGCATATAAAGCCGTTGGAC	6571
Wm5      GCCAAGGTCCGTGACTATCATGTTTGTATATATCAGAGAAAGCATATAAAGCCGTTGGAC	3678
         ************************************************************

Wm1      CACTTTTATTTGCAGGTAAATAAAGGATATTTTCATCTTTAATTCTCTTAAATGAAATTT	6631
Wm5      CACTTTTATTTGCAGGTAAATAAAGGATATTTTCATCTTTAATTCTCTTGAATGAAATTT	3738
         ************************************************* **********

Wm1      GAAAGGCTTCATCAGGTATCTTCTTAAGAAATTCTTGAGACCTTTCCATGTGTCCTTCAC	6691
Wm5      GAAAGGCTTCATCAGGTATCTTCTTAAGAAATTCTTGAGACCTTTCCATGTGTCCTTCAC	3798
         ************************************************************

Wm1      TTCTATCATGTGTTGACATCTTCAAATGTGTTAGTAAAATATTGATTAATTGCATAAAAC	6751
Wm5      TTCTATCATGTGTTGACATCTTCAAATGTGTTAGTAAAATATTGATTAATTGCATAAAAC	3858
         ************************************************************

Wm1      TCACTTTAATAGCAACTTCACTGAGTCTAATATTTAATTCATCAACTGTATTCACCGGTC	6811
Wm5      TCACTTTAATAGCAACTTCACTGAGCCTAATATTTAATTCATCAATTGTATTCACTGGTC	3918
         ************************* ******************* ********* ****

Wm1      TTGAAATACAGTTCTCATATGAATCGAAGAGTACCATTCTCGAGTAAGCATGTTCTCTTG	6871
Wm5      TTGAAATACAGTTCTCATATGAATCGAAGAGTACCATTCTCGAGTAAGCATGTTCTCTTG	3978
         ************************************************************

Wm1      CAATCTCAGTTTGAGCCGTGTCAAATAATACTGACTGTAAATCTTGATATTGCTGAGCGT	6931
Wm5      CAATTTCAGTTTGAGCCGTGTCAAATAATACTGACTGTAAATCTTGATATTGCTGAGCGT	4038
         **** *******************************************************

Wm1      GATGAGTTTCTTCAGCTATATCGTATTGATCAAAGAAGCTCTGTACATCATCAGGTACTT	6991
Wm5      GATGAGTTTCTTCAGCAATATCGTATTGATCAAAGAAGCTCTGTACATCATCAGGTACTT	4098
         **************** *******************************************

Wm1      CCAAAATGCTCGAGCCTTTTCCTGTGGAGGTTGTATTAATATATCTGCCAATCATTAGTT	7051
Wm5      CCAAAATGCTCGAGCCTTTTCCTGTGGAGGTTGTATTAATATATCTGCCAATCATTAGTT	4158
         ************************************************************

Wm1      AAAACATTCAACTAAACCTCGAGCACTTACAATGCTCTTTTATCAATATAGATTCTGATT	7111
Wm5      AAAACATTCAACTAAACCTCTAGCACTTACAATGCTCTTTTATCAATATAGATTCTGATT	4218
         ******************** ***************************************

Wm1      AGCTTTAAATGCTCAATAAAATCAATTTTATCAAGAGACAATGACCTGTTCTTCTCCATT	7171
Wm5      AGCTTTAAATGCTCAATGAAATCAATTTTATCAAGAGATAATGACCTGTTCTTCTCCATT	4278
         ***************** ******************** *********************

Wm1      TTCTTTTCTTTGACAAGATCTTTGCATAAACTCTCATATTCGTTTGCCAAACGCTCTATA	7231
Wm5      TTCTTTTCTTTGACAAGATCTTTGCATAAACTCTCATATTCGTTTGCCAAACGCTCTATA	4338
         ************************************************************

Wm1      TAGACATGTTCTTCACGATTCTCTAGTAACGTCTTCTCATGATTTCTCAACTGACCTGTC	7291
Wm5      TAGACATGTTCTTCACGATTCTCTAGTAACGTCTTCTCATGATTTCTCAACTGACCTGTC	4398
         ************************************************************

Wm1      TTAAATCTCGGGAAAACGTGTAAAGCTGCAGACAAAGATGGATTCTTGTGATTCCTAGTA	7351
Wm5      TTAAATCTCGGGAAAACGTGTAAAGCTGCAGACAAAGATGGATTCTTGTGATTCCTAGTA	4458
         ************************************************************

Wm1      CGAGCTTTTATGATAGCAATTGAATCACGCAATAATTCATCAGTCTCAGGGTTCCAATAA	7411
Wm5      CGAGCTTTTATGATAGCAATTGAATCACGCAATAATTCATCAGTCTCAGGGTTCCAATAG	4518
         ***********************************************************

Wm1      CTGTAATTCTTCGTTTGACGTGCACTTGTACCTTCAGGGACTTAAACAGTTTAATAAGTA	7471
Wm5      CTGTAATTCTTCGTTTGACGTGCACTTGTACCTTCAGGGACTTAAACAGTTTAATAAGTA	4578
         ************************************************************

Wm1      TATCATCTGTACGTAAATTAAAGTACTTACAAAGTCTCTTATGTTCTTCTATAAGCTCCT	7531
Wm5      TATCATCTGTACGTAAATGAAAGTACTTACAAAGTCTCTTATGCTCTTCTATAAGCTCCT	4638
         ****************** ************************ ****************

Wm1      CAGGTGATTTACCAGGTGGTTCAGAAAGAGGTGGATCAAGCCTATAGGGGTTCTCTCTTG	7591
Wm5      CAGGTGATTTACCAGGTGGTTCAGAAAGAGGTGGATCAAGCCTATAGGGGTTCTCTTTTG	4698
         ******************************************************** ***

Wm1      TTGGTTTACTTTTCTTTTTTTTCTCGTTTTTCTTTTTAAACTCGCGTATTTGCTCAACTA	7651
Wm5      TTGGTTTACTTTTCTTTTTCTTCTCGTTTTTCTTTCTAAACTCGCGTATTTGCTCCACTA	4758
         ******************* *************** ******************* ****

Wm1      TTGCTGCATCTTCAGCATCCTCAAAGCTATCTTTTTCAGATTCTTCGCTTCTCATGTGTC	7711
Wm5      TTGCTGCATCTTCAGCATCCTCAAAGCTATCTTTTTCAGATTCTTCGCTTCTCATGTGTC	4818
         ************************************************************

Wm1      GAGAACGCGCTTTTTTTACTTTCGCATCAATGGACCTGCTATACTTCGAAGACTTTTTAA	7771
Wm5      GAGAACGCGCTTTTTTTACTTTCGCATCAATGGACCTGCTATACTTCGAAGACTTTTTAA	4878
         ************************************************************

Wm1      GCTTTTTCGTTGTGTTTTTGTTCTTCTTAGGTCTATGAAGAGTTACTCCCAATTTAAGTT	7831
Wm5      GCTTTTTCGTTGTGTTTTTGTTCTTCTTAGGTCTATGAAGAGTTACTCCCAATTTAAGTT	4938
         ************************************************************

Wm1      TTTCTTGTTTGGAAGTTTCCTCTTCTATATAACTTTTTACAAGTGGTAAAGGTGCTCCGC	7891
Wm5      TTTCTTGTTTGGAAGTTTCCTCTTCTATATAACGTTTCACAAGTGGTAAAGGTGCTCCGC	4998
         ********************************* *** **********************

Wm1      AGGTATAGGCAATCTCCCTGTATTCCTTCTCCGTACCTGGGCGTGGTATGTCACTTCTCT	7951
Wm5      AGGTATAGGCAATCTCCTTGTATTCCTCCTCCGTACCTGGGCGTGGTATGTCACTTCTCT	5058
         ***************** ********* ********************************

Wm1      TCCCAGTATAATTAGCAAGGAGGCGTTTTTGCATTGCTGATAACATCATGTCATTTTGCC	8011
Wm5      TCCCAGTATAATTAGCAAGGAGGCGTTTTTGCATTGCTGATAACATCATGTCATTTTGCC	5118
         ************************************************************

Wm1      AACTTTGATGCTTTTGAATATACGATGCAAAATGTGAGAAGTTCGTCATATCCGGATTCT	8071
Wm5      AACTTTGATGCTTTTGAATATACGATGCAAAATGTGAGAAGTTCGTCATATCCGGATTCT	5178
         ************************************************************

Wm1      TCGATTTCTCATTTGCTGTCATTGAAGCATCTTGACTGCTCAACGCAAGCACTTTCAACC	8131
Wm5      TCGATTTCTCATTTGCTGTCATTGAAGCATCTTGACTGCTCAACGCAAGCACTTTCAACC	5238
         ************************************************************

Wm1      TTTCAAAATACGTATCTATATCTTCGTTTGTGTTGAGCGTCATACCATCGATAAGTACGG	8191
Wm5      TTTCAAAATACTTATCTATATCTTCGTTTGTGTTGAGCGTCATACCATCGATAAGTACGG	5298
         *********** ************************************************

Wm1      GTTTATCTTCAAAAGATAAAAAGAGACCATCTTTCTTCAATTTAAAATAAAGGAGCCTGT	8251
Wm5      GTTTATCTTCAAAAGATAGAAAGAGACCATCTTTCTTCAATCTAAAATAAAGGAGCCTGT	5358
         ****************** ********************** ******************

Wm1      TTGGTGTCTTTAACTCCCTTACACCCTTGAGATTACTAACATCAGTTACTATTTCATCAG	8311
Wm5      TTGGTGTCTTTAACTCCCTTACACCCTTGAGATTACTAACATCAGTTACTATTTCATCAG	5418
         ************************************************************

Wm1      TTACTTCGATCTCCTCCATGAGCTCGACAAGGTTCAAATGCCATAAGATACGGACACATT	8371
Wm5      TTACTTCGATCTCCTCCATGAGCTCGACAAGGTTCAAATGCCATAAGATACGGACACATT	5478
         ************************************************************

Wm1      CAGTAATAAAGTGACGTCTCTTTGAAAATGATAGACTGACAAGTTCTTGTAAATGCTCTG	8431
Wm5      CAGTAATAAAGTGACGTCTCTTTGAAAATGATAGACTGACAAGTTCTTGTAAATGCTCTG	5538
         ************************************************************

Wm1      GAAGGTCTTTAATCCTCAGTTTACTCTTTAAAGCATCATTCTTGTATTCAAACGCCATCT	8491
Wm5      GAAGGTCTTTAATCCTCAGTTTACTCTTTGAAGGATCATTCTTGTATTCAAACGCCATCT	5598
         ***************************** *** **************************

Wm1      CATCGAATGTTGTCCAAGGAATTAGTTTCATGTACATATTCAATGGTATGTCACGTTTGA	8551
Wm5      CATCGAATGTTGTCCAAGGAATTACTTTCATGTACATATTCAATGGTATGTCACGTTTGA	5658
         ************************ ***********************************

Wm1      AGTAGTTCATACATACGATAGGCTCATCGTCGCTCATTTTACTAGATAGCTGATCCTTCT	8611
Wm5      AGTAGTTCATACATACGATAGGCTCATCGTCGCTCATTTTACTAGATAACTGATCCTTCT	5718
         ************************************************ ***********

Wm1      GCTGCATTTCTTGCGATAAAAGGGAGTACAAAGTATGTGCTTTTGCAAACCTTCCGGTCA	8671
Wm5      GCTGCATTTCTTGCGATAGAAGGGAGTACAAAGTATGTGCTTTTGCAAACCTTCCGGTCA	5778
         ****************** *****************************************

Wm1      TGTAACCATGAAGCCTAGCCATTGCTTTAACATCAGACAAGATCAGGTTTCTGATATTTT	8731
Wm5      TGTAACCATGAAGCCTAGCCATTGCTTTAACATCAGACAAGATCAGGTTTCTGATATTTT	5838
         ************************************************************

Wm1      CTTTTTGCGATGGATCAGTGCAATTTTCAGGTAAATTGTCAGCAGCATATTTCCTCCTAA	8791
Wm5      CTTTTTGCGATGGATCAGTGCAATTTTCAGGTAAATTGTCAGCAGCATATTTCCTCCTAA	5898
         ************************************************************

Wm1      AGTTAATATATCCGATATTTTCGGTGTCTACTTCTTTGAAAGACGCTTTAGGTTGGTATC	8851
Wm5      AATTAATATATCCGATATTTTCGGTGTCTACTTCTTTGAAAGACGCTTTAGGTTGGTATC	5958
         * **********************************************************

Wm1      TCTTCATTAAATCTTGCCCCAACTCTTCTTTCTTTTTGCGAAAAGCCTCCGAATGTGTAT	8911
Wm5      TCTTCATTAAATCTTGCCCCAACTCTTCTTTCTTTTTGCGAAAAGCCTCCGAATGTGTAT	6018
         ************************************************************

Wm1      CAATATCTGATACGTAAATAAGTCTTTTCTGCATTGAAGCGCCTCCATAAGTTCTACTGG	8971
Wm5      CAATATCTGATACGTAAATAAGTCTTTTCTGCATTGAAGCGCCTCCATAAGTTCTACTGG	6078
         ************************************************************

Wm1      ATACCTTTGTTTCTTTCAATTTACCTTCATTAAACAACACATTGAGAGTTTTTCTAACAA	9031
Wm5      ATACCTTTGTTTCTTTCAATTTACCTTCATTAAACAACACATTGAGAGTTTTTCTAACAA	6138
         ************************************************************

Wm1      CTGGCCTATCGATTTGTGCCGCAGTTTCGTGATTGGGGCCATGTACATACTTCTGATACA	9091
Wm5      CTGGCCTATCGATTTGTGCAGCAGTTTCGTGATTGGGGCCATGTACATACTTCTGATACA	6198
         ******************* ****************************************

Wm1      CATCAAACAATTCGCGCACCGCTTCCACCATACCACCAGCACTCTTTATTGCATTGTATA	9151
Wm5      CATCAAACAATTCGCGCACCGCTTCCACCATACCACCAGCACCCTTTATTGCATTGTATA	6258
         ****************************************** *****************

Wm1      AATCTTGAATTCTACGATGTTGTGAAAGATCTGTTCTTGATGCTCGCATTTGCTGAATAG	9211
Wm5      AATCTTGAATTCTACGATGTTGTGAAAGATCTGTTCTTGATGCTCGCATTTGTTGAATAG	6318
         **************************************************** *******

Wm1      CTCTCTTCGTTGTCTTGCTTCTAGGGGTTCCATTTGCTAATTTTGATGTTGATGTCATAG	9271
Wm5      CTTTCTTCGTTGTCTTGCTTCTAGGGATTCCATTTGCTAATTTTGATGTTGATGTCATAG	6378
         ** *********************** *********************************

Wm1      TTGATTCCATTTGATTATCTGAAATGGCAGTATCTGCATCCCTCTTAACATCTAGCTCGT	9331
Wm5      TTGATTCCATTTGATTATCTGAAATGGCAGTATCTACATCCCTCTTAACATCTAGCTCGT	6438
         *********************************** ************************

Wm1      TAATTTCGGATGCGTTCTGTTGTTGAGATGTGTCATATTGTAATTTGTTTACTTGATTTT	9391
Wm5      TAATTTCGGATGCGTTCTGTTGTTGAGATGTGTCATATTGTAATTTGTTTACTTGATTTT	6498
         ************************************************************

Wm1      CAACCTCAGTTATGACTTTTGGTTGAGGCTCCTGTATATTACCCTTCTCATCTTCGACAC	9451
Wm5      CAACCTCAGTTATGACTTTTGGTTGAGGCTCCTGTATATTACCCTTCTCATCTTCGACAC	6558
         ************************************************************

Wm1      CTTGATTTTCGGTAGTCTCGTCTTCCTTTTCATGATGGTCTTCACCAGGTTCATATGCAT	9511
Wm5      CTTGATTTTCGGAAGTCCCGTCTTCCTTTTCATCATGGTCTTCACCTGGTTCATATGCAT	6618
         ************ **** *************** ************ *************

Wm1      CGGACTCATCACTATCTTGTGGACTTTGAACTCTTTTTCGTTTTGTTGATATATGATCTT	9571
Wm5      CGGACTCATCACTATCTTGTGGACTTTGAACTCTTTTTCGTTTTGTTGATATATGATCTT	6678
         ************************************************************

Wm1      GACTAACATCTTCGACATCAATTACAATAGGAGCGATCCCACTTTTCCTTGATCTAAGTT	9631
Wm5      GACTAACATCTTCGACATCAATTAAAATAGGAGCGATCCCACTTTTCCTTGATCTAAGTT	6738
         ************************ ***********************************

Wm1      TGCGTTTTTTAAGGTATGAATCCTTATCTACATCGTTTGTAACATTGATAGTATTATGTG	9691
Wm5      TGCGTTTTTTAAGGTATGAATCCTTATCTACATCGTTTGTAACATTGATAGTATTATGTG	6798
         ************************************************************

Wm1      CGACTTCTTGTTTATTTAGTTGTTGTCTTTTATTAGGCGTTGACATGTGACTATTATCGT	9751
Wm5      CGACTTCTTGTGTATTTAGTTGTTGTCTTTTATTAGGCGTTGACATGTGACTATTATCGT	6858
         *********** ************************************************

Wm1      CACCGACCATATCAAGTTCAGGATTGTGATGAGCTCTTTGAGGTGCAGTTTGTGATGTTG	9811
Wm5      CACCGACCATATCAAGTTCAGGATTGTGATAAGCTCTTTGAGGTGCAGTTTGTGATGTTG	6918
         ****************************** *****************************

Wm1      TTAAAGACACTTCAGATGGGGGCTGTGTTTCTTCTGGCGTAGCATTCAATGGTATGCTTT	9871
Wm5      TTAAAGACACTTCAGATGGGGGCTGTGTTTCTTCTGGCGTAGCATTCAATGGTATGCTTT	6978
         ************************************************************

Wm1      CAGCTACATTTTCCTGTAATTCAGAATGTTTCTTTGTCACGTCCTTTACAGTTTTTTCAT	9931
Wm5      CAGATACATTTTCCTGTAATTCAGAATGTTTCTTTGTCACGTCCTTTACAGTTTTT----	7034
         *** ****************************************************

Wm1      CATTATCATCATCATCATCATCATCATCATCTACTTCTATTGTATTTTTTGAAGCACGAC	9991
Wm5      --------TCATCATCATCATCATCATCATTTATTTCTATTGTATTTTTTGAAGCACGAC	7086
                 ********************** ** **************************

Wm1      CTTTCTTTTTAGGCTGATCTTCGATGATTTCGGACTTCCGTGGACGGCCTCTCTTCTTTG	10051
Wm5      CTTTCTTTTTAGGTTGATCTTCGATGATTTCGGACTTCCGTGGACGGCCTCTCTTCTTCG	7146
         ************* ******************************************** *

Wm1      GTACCTCTGATGGTGTTTGGCTTGCTTCAGACTTACGAGGACGACCTTTAGGCTTTGGTG	10111
Wm5      GTACCTCTGATGGTGTTTGGCTTGCTTCAGACTTACGAGGACGACCTTTAGGCTTTGGTG	7206
         ************************************************************

Wm1      TAGGCATTGCTGATTTGGATGATTCTGCTTTCGGAGGTCTACCAAGTTTTTTAGGAGTAG	10171
Wm5      TAGGCATTGCTGATTTGGATGATTCCGCTTTCGGAGGTCTACCAAGTTTTTTAGGAGTAG	7266
         ************************* **********************************

Wm1      CCACCTGCCCACCGATCGATCCTATCATGCGAGTTGCTGACGCCTGGTACTCTTGACGAG	10231
Wm5      CCACCTGCCCACCGATCGATCCTATCATGCGAGTTGCTGACGCCTGGTACTCTTGACGAG	7326
         ************************************************************

Wm1      ACGAACACCCCAAGGCAGAATTAATAACCTTCAATGAACCAGATTGATTGGAAACCAAGT	10291
Wm5      ACGAACACCCCAAGGCAGAATTAATAACGTTCAATGAACCAGATTGATCGGAAACCAAGT	7386
         **************************** ******************* ***********

Wm1      CTTCAACTGGAATATCTAACCCTTCTTTTTCTGCTAAAAGTATATAATTCTTACGAGTAA	10351
Wm5      CTTCAACTGGAATATCTAACCCTTCTTTTTCTGCTAAAAGTATATAATTCTTGCGAGTAA	7446
         **************************************************** *******

Wm1      AAAATCGTTGCCTGCGCTCTCGACCATTCATTTCTGTGGTTGATTTTAGTGCAAGGTCAG	10411
Wm5      AAAATCGTTGCCTGCGCTCTCGACCATTCATTTCTGTGGTTGATTTTAGTGCAAGGTCGG	7506
         ********************************************************** *

Wm1      TGAGGCTAGCTGGATCATGATCTGTCTCATGACGCAAAAGTATGTTTTCGACAGTTCTCC	10471
Wm5      TGAGGCTAGCTGGATCATGATCTGTCTCATGACGCAAAAGGATGTTTTCGACAGTTCTCC	7566
         **************************************** *******************

Wm1      TATTGATGGTAGGAAGAGCGCTAATAACTGACTAGCAAGATGTTAAACAATACATTCTAT	10531
Wm5      TATTGATAGTAGGAAGCGCGCTAATAACTGACTAGCAAGATGTTAAACAATACATTCTGT	7626
         ******* ******** ***************************************** *

Wm1      TTACCTTCTCAACTTACAGAGATAGTCATCCCTTCAATACCTGATTCATGTATAAGGTTT	10591
Wm5      TTACCTTTTCAACTTACAGAGATAGTCATCCCTTCAATACCTGATTCATGTATAAGGTTT	7686
         ******* ****************************************************

Wm1      AATATTTGTTTTTCCACTGTGATAGCAATCCCAGCTCCAAAATTATCTGAAACAACGTCA	10651
Wm5      AATATTTGTTTTTCCACTGTGATAGCAATCCCAGCTCCAAAATTATCTGAAACAACGTCA	7746
         ************************************************************

Wm1      TCATCATCGTCTCCATCATCAGATTCTAATTTAATCATATCTTCCTCAATGACTTTCTCA	10711
Wm5      TCATCATCATCTTCATCATCAGATTCTAATTTAATCATATCTTCCTCAATGACTTTCTCA	7806
         ******** *** ***********************************************

Wm1      GACGACTTTGATCCGTTATCATCATCTGCAATACGCAAACAGTTAACACGTGTGTCCCTA	10771
Wm5      GACGACTTTGATCCGTTATCATCATCTGCAATTCGCAAACAGTTAACACGTGTGTCCCTA	7866
         ******************************** ***************************

Wm1      CCTCCAACTTTGGTAGCACATTTTTCGAGTGAACCCTCTGCAACCATTCTGCCTATAAGA	10831
Wm5      CCTCCAACTTTGGTAGCACATTTTTCGAGTGAACCCTCTGCAACCATTCTGCCTATGAGA	7926
         ******************************************************** ***

Wm1      CGTGCCACGAGTCTTTGAATAGGTTTACTATTTGTTTTAAATCCTATTTTGTTAAATATA	10891
Wm5      CGTGCAACGAGTCTTTGAATAGGTTTACTATTTGTTTTAAATCCTATTTTGTTAAATATA	7986
         ***** ******************************************************

Wm1      TACTTATGTTGAATAATCTTGTTCGGACAGCTATTGATAAGTTTTAAGATACGGCCTTTC	10951
Wm5      TACTTATGTTGAATAATCTTGTTCGGACAGCTATTGATAAGTTTTAAGATACGGCCTTTC	8046
         ************************************************************

Wm1      AACAAATTGTTGTTGGTGAGATGTTCCTTACTTAATTCTGAGAATTCTGGTAGACCTGAG	11011
Wm5      AACAAATTGTTGTTGGTGAGATGTTCCTTACTTAATTCTGAGAATTCTGGTAGACCTGAG	8106
         ************************************************************

Wm1      GCGTCACGAGCTTGTACTGTTTCTTCAAAATCTATTGCTTCATTGTCGGAATCATTTTTT	11071
Wm5      GCGTCACGACCTTGTACTGTTTCTTCAAAATCTATTGCTTCATTGTCGGAATCATTTTTT	8166
         ********* **************************************************

Wm1      GCAGGTTCTGCTTCTGTGAGTGCATGTGCTCGATAGTGTGGTGAGCTGGCCAAAAATTTA	11131
Wm5      GCAGGTTCCGCTTCTGTGAGTGCATGTGCTCGGTAGTGTGGTGAGCTGGCCAAAAATTTA	8226
         ******** *********************** ***************************

Wm1      CGATGTATAGCAACATTTGTGGTGCTACCTCTGTAGACAGTGTGCATTTTTACGCTTATG	11191
Wm5      CGATGTATAGCAACATTTGTGGTGCTACCTCTGTAGACAGTGTGCATTTTTACGCTAATG	8286
         ******************************************************** ***

Wm1      ATACTGTCAATTGAGGGCTAGATCAGATTGTTAACTTACACAAGATCCAATTCTATAAGT	11251
Wm5      ATACTGTCAATTGAGGGCTAGATCAGACTGTTAACTTACACAAGATCCAATTCTATAAGT	8346
         *************************** ********************************

Wm1      ATTTTGACACAATAAAATACAGTTTTGCTGTCATAACCAGTCATCTTGCTGATCTGCATA	11311
Wm5      ATTTTGACACAATAAAATACAGTTTTGCTGTCATAACCGGTCATCTTGCTGATCTGCATA	8406
         ************************************** *********************

Wm1      AAGGTGATACCATTTTCTCTACTTCTAGAGATAAAAGTGAGCACACTCAATACCATCTGG	11371
Wm5      AAGGTGATACCATTTTCTCTACTTCTAGAGATAAAAGTGAGCACACTCAATACCATTTGG	8466
         ******************************************************** ***

Wm1      CTAAGTTTTGCTGGTTTATGATTAAGACCAGTTATCACAAACCAATTCATGTCACTATCA	11431
Wm5      CTAAGTTTTGCTGGTTTATGATTAAGACCAGTTATCACAAACCAATTCATGTCACTATCA	8526
         ************************************************************

Wm1      GTACGAACGCGAATATCGTTACCGTATCGTTCAAGAAGGGAGGCCAAAGATGTTTGTGTA	11491
Wm5      GTACGAACGCGAATATCGTTACCGTATCGTTCAAGAAGGGAGGCCAGAGATGTTTGTGTA	8586
         ********************************************** *************

Wm1      TCATCGAAGTTGAGTAGCTCAAACATGATAGCTTGATTACCGTCTTTAGAAGATATCGGA	11551
Wm5      TCATCGAAGTTGAGTAGCTCAAACATGATAACTTGATTACCATCTTTAGAAGATATCGGA	8646
         ****************************** ********** ******************

Wm1      GGGGCTTTTTCCTTTAAGGCTAGCTTCAACTGTTCTGTTGCTAAGCCAATTTGAAATGAG	11611
Wm5      GGGGCCTTTTCCTTTAAGGCTAGCTTCAACTGTTCTGTTGCTAAGCCAATTTGAAATGAG	8706
         ***** ******************************************************

Wm1      GGTTGTTGAGTGAGAATTCTCCAGAAGAATTCAAGTAACGGACCATTGTTGATATCTACC	11671
Wm5      GATTGTTGAGTGAGAATTCTCCAGAAGAATTCAAGTAACGGACCATTGTTGATATCTACC	8766
         * **********************************************************

Wm1      GAAGGATCTAACTGTGCTTTGATGTATTTCACAAGAGTTTGTATATCGGAGCCTATGATT	11731
Wm5      GAAGGATCTAACTGTGCTTTGATGTATTTCACAAGAGTTTGTATATCGGAGCCTATGATT	8826
         ************************************************************

Wm1      TGTTAGCAACCAGAGATTCGAACACCGGCACCTGCTGACGGCGCCTGATAACGAAAGTGA	11791
Wm5      TGTTAGCAACCAGAGATTCGAACACCGGCACCTGCAGACGGCGCCTGATAACGAAAGTGA	8886
         *********************************** ************************

Wm1      CGTACCCCAGTCACCATCTACTGAGATCTCATTTAAAACGTTATGTACTACTACGTCTGC	11851
Wm5      CGTACCCCAGTCACCATCTACTGAGATCTCATTTAAAACGTTATGTACTACTACGTCTGC	8946
         ************************************************************

Wm1      CGCAATCATTATAAGAAGCTTCCTATATAGCTTTACGAGTCGCTAGTACGATTATGCGAG	11911
Wm5      CGCAATCATTGTAAGAAGCTTCCTATATAGCTTTACGAGTCGCTAGTACGATTATGCGAG	9006
         ********** *************************************************

Wm1      TACTTGTATTTAACGTATATTAATTTTATTTATTTACGAGAGTTATTTCTTCTTTATCTC	11971
Wm5      TACTTGTATTTAACGTATATTAATTTTATTTATTTACGAGAGTTATTTCTTCTTTATCTC	9066
         ************************************************************

Wm1      TCGAATTAATAATTCACTCCTGATGGTTGAAATCAGAGATAGGGCCAAATGCGAGGAAGT	12031
Wm5      TCGAATTAATAATTCACTCCTGATGGTTGAAATTAGAGATAGGGCCAAATGCGAGGAAGT	9126
         ********************************* **************************

Wm1      GTATGAGAACAAAAATTACATTAAAAAAGGAGATCTTCTAAACCACTATTCTTATTCTGC	12091
Wm5      GTATGAGAACAAAAATTACATTAAAAAAGGAGATCTTCTAAACCACTATTCTTATTCTGC	9186
         ************************************************************

Wm1      CCTTGTGTGTTGTTTGTTGTTGTCATATCGAAAATGTCGTTATTTGCGCCGACGGACTGA	12151
Wm5      CCTTGTGTGTTGTTTGTTGTTGTCATATCGAAAATGTCGTTATTTGCGCCGACGGACTGA	9246
         ************************************************************

Wm1      CTAGGACTCGAAACGGTGGCATTTCCGAATAATCCTAATAAGTCATCCACAGGATTAGAC	12211
Wm5      CTAGGACTCGAAACGGTGGCATTTCCGAATAAGCCTAATAAGTCATCGACAGGATTAGAC	9306
         ******************************** ************** ************

Wm1      TGTGCGGGACTTGGTGCATGGTTAATATTACTTGTCGCCGTATTGGCTGTGAAACTGTTG	12271
Wm5      TGTGCGGGACTTGGTACATGGTTAATATTACTTGTCGCCGTATTGGCTGTGAAACTGTTG	9366
         *************** ********************************************

Wm1      AAGCTTTCTTGTGGACTTTGTGTCGTGGATGGTGAGGCATCTTCATCAAGGTCTAATAAA	12331
Wm5      AAGCTTTCTTGTGGACTTTGTGTCGTGGATGGTGAGGCATCTTCATCAAGGTCTAATAAA	9426
         ************************************************************

Wm1      TTTTCTGCTTTAACACCTACATGAGAGTTTAGAAGAATGCGCAATCACAAATTTCATAGA	12391
Wm5      TTTTCTGCTTTAACTCCTACATGAGAGTTTAGAAGAATGCGCAATCACAAATTTCATAGA	9486
         ************** *********************************************

Wm1      TACTTACCTTGTGCCACCGTGTTCAATGCTTTTTGTTTCGAAATATTCTCATTTTCTTCA	12451
Wm5      TACTAACCTTGTGCCACCGTGTTCAATGCTTTTTGTTTCGAAATATTTTCATTTTCTTCA	9546
         **** ****************************************** ************

Wm1      TTCGGATTAATCTTCTTTATTGACTCAATACCAAGACGACCTTTACCAACGAAAGTAGAT	12511
Wm5      TTCGGATTAATCTTCTTTATTGACTCAATACCAAGACGACCTTTACCAACGAAAGTAGAT	9606
         ************************************************************

Wm1      GCTGGCTTGTGATATGCGGCTGCAAGGGAAGAAATTTCTTGAACTAGTTCTTCTAGGATT	12571
Wm5      GCTGGCTTGTGATATGCGGCTGCAAGGGAAGAAATTTCTTGGACTAGTTCTTCTAGGATT	9666
         ***************************************** ******************

Wm1      GCCCGCGAGACTGTTAAGTTTGGAATGACGATAGGTGGCCTATCCGCTATAGTAATTGAC	12631
Wm5      GCCTGCGAGACTGTTAAGTTTGGAATGACGATAGGTGGCCTATCCGCTATAGTAATTGAC	9726
         *** ********************************************************

Wm1      TATAATCGTTAGTCAGTTCTAGCACTTAATTAGCACTCACTCTTGCTGCTCCAGTATCTG	12691
Wm5      TATAATCGTTAGTCAGTTCTAGCACTTAATTAGCACTCACTCTTGCTGCTCCAGTATCTG	9786
         ************************************************************

Wm1      TTGAAAGTAGTCTCCAGTAAATAAATGCACGATCACGTACATCTGAGTTATCACAGTCAC	12751
Wm5      TTGAAAGTAGTCTCCAGTAAATAAATGCACGATCACGTACATCTGAGTTATCACAGTCAC	9846
         ************************************************************

Wm1      TTGTAGCTGTCGTCAAAACCCTTTGAACCAATGACTGAGAAGGTCCCTCTGGCTTCTTTA	12811
Wm5      TTGTAGCTGTCGTCAAAACCCTTTGAACCAATGACTGAGAAGGTCCCTCTGGCTTCTTTA	9906
         ************************************************************

Wm1      AAAACAGTTTTACTATCGCACTTAAGGTTTGTAATTGAACCTAACATTATGAGCTTATCG	12871
Wm5      AAAACAGTTTTACTATCGCACTTAAGGTTTGTAATTGAACCTAACATTATGAGCTTATCA	9966
         ***********************************************************

Wm1      CATGCTTTAATATATTCACTCACTGGGTAAGAGTCCTCGATAAAGCTATCCAGATACGTC	12931
Wm5      CATGCTTTAATATATTCACTCACTGGGTAAGAGTCCTCGATAAAGCTATCCAGATACGTC	10026
         ************************************************************

Wm1      TCTAATAACTCCTCGACATTTACTATTTTCTCTGCATTTTCACCTAGGATCCAAATCAAT	12991
Wm5      TCTAATAACTCCTCGACATTTACTATTTTCTCTGCATTTTCACCTAGGATCCAAATCAAT	10086
         ************************************************************

Wm1      GACGCTTTGGATTCAGGTTCATCAATTTCTTCGAGGTTTTCACAAAGTGTTGGAATAATT	13051
Wm5      GACGCTTTGGATTCAGGTTCATCAATTTCTTCGAGGTTTTCACAAAGTGTTGGAATAATT	10146
         ************************************************************

Wm1      GCTTCATATGAGTGTGGGTATTTACGGAATATATCCTAGATAAAGGTCAACCAAAATCCA	13111
Wm5      GCTTCATATGAGTGTGGGTATTTACGGAATATATCCTAGATAAAGGTCAGCTAAAATCCA	10206
         ************************************************* * ********

Wm1      CGTTCGAGAATAGTGTTTCTCACCTTTATGACTATGACTGCTTCTTGTACAACGTATGAT	13171
Wm5      AGTTCGAGAATGGTGTTTCTCACCTTTATGACTATGACTGCTTCTTGTACAACATATGAT	10266
          ********** ***************************************** ******

Wm1      ACTTTTGTATTGATCAAGTCGACGAGAACTTGTACACAACGCTCTGCAGCATCTTCAATT	13231
Wm5      ACTTTTGTGTTGATCAAGTCAACGAGAACTTGTACACAACGCTCTGCAGCATCTTCAATT	10326
         ******** *********** ***************************************

Wm1      TTGATTGCGCACCGTCCTATAGCTCTTATCGATCTGCGAACGAAATCAACATCAACTTCA	13291
Wm5      TTGATTGCGCACCGTCCTATAGCTCTTATCGATCTGCGAACGAAATCAACATCAACTTCA	10386
         ************************************************************

Wm1      CTAGCATATTCCTTAAGCTCGTTTAATAGAGTCTCGACATTGTTCTCATTAGCTAATTTA	13351
Wm5      CTAGCATATTCCTTAAGCTCGTTTAATAGAGTGTCGACATTGTTCTCATTAGCTAATTTA	10446
         ******************************** ***************************

Wm1      ACCAATATGTCTAATTTTTCAACTTTGTTGTATTGTGCATCGGAGTACTTGCAGAAAAAG	13411
Wm5      ACCAATATGTCTAATTTTTCAACTTTGTTGTATTGTGCATCGGAGTACTTGCAAAAAAAG	10506
         ***************************************************** ******

Wm1      ACGCGCAATTCTGAAGAAAGAACGTCTGGACGAGCTTGTAATATCAAATTTATGTTTCTA	13471
Wm5      ACGCGCAATTCTGAAGAAAGAACGTCTGGACGAGCTTGTAATATCAAATTTATGTTTCTA	10566
         ************************************************************

Wm1      AGAGCTACCCATTGTACCTCAGGCTCTGATGCCACAAGAGTGACTGAAAAATTGTCAAAT	13531
Wm5      AGAGCTACCCATTGTACCTCAGGCTCTGATGCCACAAGAGTGACTGAAAAATTGTCAAAT	10626
         ************************************************************

Wm1      TACCAAGTAGTAAATGCATTGTACTTACCTAGAGGGGGTGCCATCTTACGAACGAGCTGT	13591
Wm5      TACCAAGTAGTAAATGCATTGTACTTACCTAGAGGAGGTGCCATCTTACGAACGAGCTGT	10686
         *********************************** ************************

Wm1      CTTATAAAGTCTTCCCTTTGTAAACGTTGTAAATTAATCATAACGACTTTAATAGCACTC	13651
Wm5      CTTATAAAGTCTTCCCTTTGTAAACGCTGTAAATTAATCATAACGACTTTAATAGCACTC	10746
         ************************** *********************************

Wm1      AAAACAACACTACCATTGGCATGTTGAAATTGTGGTAAAACACGTTCGCAAATGTATTCA	13711
Wm5      AAAACAACACTACCATTGGCATGTTGAAATTGTGGTAAAACACGTTCGCAAATGTATTCA	10806
         ************************************************************

Wm1      GTTTCCTTCTCCTCTGTTGGTGTATAGCGACACAACGCGCTCAATATTGATATTCTGCCC	13771
Wm5      GTTTCTTTCTCCTCTGTTGGTGTATAGCGACACAACGCGCTCAATATTGATATTCTGCCC	10866
         ***** ******************************************************

Wm1      CATTCGGTGCATTCATTAAGAGCGACTAGGATCTTGTTAAGTATGTCCCTATCAATTATG	13831
Wm5      CATTCGGTGCATTCATTAAGAGCAACTAGGATCTTGTTAAGTATGTCCCTATCAATTATG	10926
         *********************** ************************************

Wm1      AAACCTGGTGAATCTGGATGAGGGGAATTATGAATATCAGTCAAGGCAGTGACGGCATTT	13891
Wm5      AAACCTGGTGAATCTGGATGAGGGGAATTATGAATATCAGTCAAGGCAGTGACGGCATTT	10986
         ************************************************************

Wm1      GCAACAACCTGGGGAATAATGAGATGATTGAATAATACGACAGTAATTTTCTTACAGTCG	13951
Wm5      GCAACAACCTGGGGAATAATGAGATGATTGAATAATACGACAGTAATTTTCTTACAGTCG	11046
         ************************************************************

Wm1      GATTTGGATCACCAACAAGTTCTTGAAGCGTTTCAACAAATCCTCGATCAATCGCAAGTG	14011
Wm5      GATTTGGATCACCAACAAGTTCTTGAAGCGTTTCAACAAATCCTCGATCAATCGCAAGTG	11106
         ************************************************************

Wm1      AAGGTTTAAGATCGTACATTTTGGCAACGCATAGAGCAGCTGTTTTGCGTACATATGGGT	14071
Wm5      ATGGTTTAAGATCGTACATTTTGGCAACGCATAGAGCAGCTGTTTTGCGTACATATGGGT	11166
         * **********************************************************

Wm1      TATCATCTTGTAGACCTGCAGCAAGTGGATCTGAGAGATAGTCGATAATCTTTTCTGCTC	14131
Wm5      TATCATCTTGTAGACCTGCAGCAAGTGGATCTGAGAGATAGTCGATAATCTTTTCTGCTC	11226
         ************************************************************

Wm1      TGAGACATCCCATAGTTCTTATAGATAATGCACGGACTAATGGATTAGGATCGGAAGCAT	14191
Wm5      TGAGACATCCCATAGTTCTTATAGATAATGCACGGACTAATGGATTAGGATCGGAAGCAT	11286
         ************************************************************

Wm1      CCTAAGTGTTGTTAAGCGATAAATCACAGCCAATTGTCTCACCAACCTTGACAAATGTGT	14251
Wm5      CCTAAGTGTTGTTAAGCGATAAATCACAGCCAATTGTCTCACCAACCTTGACAAATGTGT	11346
         ************************************************************

Wm1      TCACGGCAAGTATAACGAGCTCTGGCTGTGTTTTTGCGTAATTCATAAGATAAAGATAAA	14311
Wm5      TCACGGCAAGTATAACGAGCTCTGGCTGTGTTTTTGCGTAATTCATAAGATAAAGATAAA	11406
         ************************************************************

Wm1      CGAGCTTCTTCTGCTCCAGATCTTCTGTCTGCATGTTCTTCAGTACGTCTGGAAATAAAC	14371
Wm5      CGAGCTTCTTCTGCTCCAGATCTTCTGTCTGCATGTTCTTCAGTACGTCTGGAAATAAAC	11466
         ************************************************************

Wm1      CCGATACGTCTTTTCCAACAGTCTGAGAGATATCAGAGATGCACGCACTATAAGAAATAA	14431
Wm5      CCGATACGTCTTTTCCAACAGTCTGAGAGATATCAGAGATGCACGCACTATAAGAAATAA	11526
         ************************************************************

Wm1      CCAACCATATTCGCGATAACACGTTTAATAGCATCCTTTCTTTTGTCGCGGTATTCACTA	14491
Wm5      CTAACCATATTCGCGATAACACGCTTAATAGCATCCTTTCTTTTGTCGCGGTATTCACTA	11586
         * ********************* ************************************

Wm1      TTTAAGTCTGGCTACGTTAACTGCAGGTAGTGCGGAGATATCGTGATAGCATACCTGCAC	14551
Wm5      TTTAAGTCTGGCTACGTTAACTGCAGGTAGTGTGGAGATATCGTGATAGCATACCTGCAC	11646
         ******************************** ***************************

Wm1      GGAGCTCATAGTTCTCTCCTAAATAATAGATCAGAGCTGCTTACTTGGGATAGCTGTTCT	14611
Wm5      GGAGCTCATAGTTCTCTCCTAAATAATAGATCAGAGCTGCTTACTTGGGATAGCTGTTCT	11706
         ************************************************************

Wm1      ACCCACCTTTCTTCGCTGTGAAGACGTTTCTTCTTATCGTATTCCTGCCGTTGGGTTAGG	14671
Wm5      ACCCACCTTTCTTCGCTGTGAAGACGTTTCTTCTTATCGTATTCCTGCCGCTGGGTTAGG	11766
         ************************************************** *********

Wm1      AAAGCTGGGAAAGGAGATATGGCCACCTACATTTTGTAGCTAAGTAGTTTTTTTATTGCA	14731
Wm5      AAAGCTGGGAAAGGAGATATGGCCACCTACATTTTGTAGCTAAGTAGTTTTTTTATTGCA	11826
         ************************************************************

Wm1      ACGCCAGTTCAGGCGAATAAATTGATTTCTTACTGCGTTGCTGTGATAGAAAGAAGATCG	14791
Wm5      ACGCCAGTTCAGGCGAATAAATTGATTTCTTACTGCGTTGCTGTGATAGAAAGAAGATCG	11886
         ************************************************************

Wm1      TAGCTCTCCTCTTTAATTTTGGCCGTCATCTAAAATATGCCAAAAGCATTGAAATCAGCT	14851
Wm5      TAGCTCTCCTCTTTAATTTTGGCCGTCATCTAAAATATGCCAAAAGCATTGAAATCAGCT	11946
         ************************************************************

Wm1      ACAGAGAAGTTCTCCAGGAGCCATCAGCCTGCTGCTAAATCAGATAGCAATTCAGGCGGT	14911
Wm5      ACAGAGAAGTTCTCCAGGAGCCATCAGCCTGCTGCTAAATCAGATAGCAATTCAGGCGGT	12006
         ************************************************************

Wm1      ACCAGCAATCCACTTTTGTAGGCAATTCCGTTATCAGCATCTACAATCACTCATTTAGGA	14971
Wm5      ACCAGCAATCCACTTTTGTAGGCAATTCTGTTATCAGCATCTACAATCACTCATTTAGGA	12066
         **************************** *******************************

Wm1      CAGTAACACAGACAGATTCGGTCAGCATATTCTCAAGAATCCTCTAGTAGCTCAAGCGTG	15031
Wm5      CAGTAACACAGACAGATTCGGTCAGCATATTCTCAAGAATCCCCTAGTAGCTCAAGCGTG	12126
         ****************************************** *****************

Wm1      GGTATCATTAGATTATTACTGCACTTCTGAACTCTCAAAAGTATCGTTGATAAAGCTTCT	15091
Wm5      GGTATCATTAGATTATTACTGCACTTCTGAACTCTCAAAAGTATCGTTGATAAAGCTTCT	12186
         ************************************************************

Wm1      TTGCGAGCAACAGACAAAGTATTCGAAATCGGTCCAGGTACTGGAAATTTGACTGCTAGA	15151
Wm5      TTGCGAGCAACAGACAAAGTATTCGAAATCGGTCCAGGTACTGGAAATTTGACTGCTAGA	12246
         ************************************************************

Wm1      ATACTTGAAAAAGCAAAGTCTTGTCAAGTAATAGAAATGGATCCTAGAATGGCTGCCGAA	15211
Wm5      ATACTTGAAAAAGCAAAGTCTTGTCAAGTAATAGAAATGGATCCTAGAATGGCTGCCGAA	12306
         ************************************************************

Wm1      CTCAGCAAAAGATTTCAGGGAAAGTGAGTATCAATGGAAATCATCAATACTTGCTAAATA	15271
Wm5      CTCAGCAAAAGATTTCAGGGAAAGTGAGTATCAATGGAAATCATCAATACTTGCTAAATA	12366
         ************************************************************

Wm1      TATTAGACCTGAGCAGCGTAGATTAGAAATCGTCATCGGTGATTTTGTCAAGGCAGATTT	15331
Wm5      TAGTAGACCTGAGCAGCGTAGATTAGAAATCGTCATCGGTGATTTTGTCAAGGCAGATTT	12426
         ** *********************************************************

Wm1      GCCTTTCTTTGACGTAGTGTGAGTTCAGAACTCTAAAAAGCACACATGTCTGACATAAGC	15391
Wm5      GCCTTTCTTTGACGTAGTGTGAGTTCAGAACTCTAAAAAGCACACATGTCTGACATAGGC	12486
         ********************************************************* **

Wm1      AGAATATCCAATTGCCCATACTCAATCTCTTCACATATCGTGTTTCGTATTCTTTCCCAT	15451
Wm5      A-----------------------------------------------------------	12487
         *

Wm1      AGACCTATCTGTAGGGTCGCGATACTGATGTTTCAGCGTGAATTCGCCTTGCGATTATGC	15511
Wm5      ------------------------------------------------------------	12487


Wm1      GCAAGACCTGGTACTGAGATGTGGTGTCGCCTCAGTGCCAACGCCCAGTTATATTCAAAA	15571
Wm5      ------------------------------------------------------------	12487


Wm1      ATCGATCATATTATGAAAGTCAGCAAGGGCAACTTCAGACCACCGCCACAAGTCGAAAGT	15631
Wm5      ------------------------------------------------------------	12487


Wm1      AGCGTTGTAAGAATTGTCCCCTTAGATCCCCCACCGCCAGTCAAGTTTGAAGAATTCGAC	15691
Wm5      ------------------------------------------------------------	12487


Wm1      GGGTACGTCTTATTTTTTTCACTTTAGCTATTTAACTGTTTCATAGTCTCACCAGAGTGC	15751
Wm5      ------------------------------------------------------------	12487


Wm1      TTTTCACAAGAAGGAACAAGACATGCAGATCAAATTTTACCGCTGCTGGAGTTTATGATA	15811
Wm5      ------------------------------------------------------------	12487


Wm1      TGCTAGAAAGCAATAGGAAAGCTTGGTTGACTGAGAAAAATGAAGTATGTTGGATTATTA	15871
Wm5      ------------------------------------------------------------	12487


Wm1      CCAGAGATATTAATTAATTGACTGGTATGTAGATGATAGACGACTCAGAGAACATTAAGA	15931
Wm5      ------------------------------------------------------------	12487


Wm1      GCAGAGTCGAAAGGATATTAGTACAAACTGGCTTTGCAGAAAGCCGTGCCGCGAAAATGG	15991
Wm5      ------------------------------------------------------------	12487


Wm1      ACGTTGATGATTTCCTTAAGTAAGTGCGATCCGTTCCTCCTATACATGCTAACTTTGGGA	16051
Wm5      ------------------------------------------------------------	12487


Wm1      TTTCAGGTTACTTAGTGCATTCCACGATGAGAACATTCACTTTGGTTAATTAATTTATAA	16111
Wm5      ------------------------------------------------------------	12487


Wm1      GTGATTAGATATATATTTAATTTTATTCATACACTACAATCCTATTCGATTTTGTTGAGA	16171
Wm5      ------------------------------------------------------------	12487


Wm1      AAACCAAAACATTTGAAAATTTTACTCGTCAATGACCTTTCTAGCGCGACTTCTAGTCCT	16231
Wm5      ------------------------------------------------------------	12487


Wm1      ATAAGCAGGTGAGTTATTTGGAGAAGACTCCACTGGCTCTACTGGGGCTTTCACTGGAGC	16291
Wm5      ------------------------------------------------------------	12487


Wm1      TGCTTTTGGTGTAGCTTCTTCTGTCTTCTTGTCCTTAGCTGAGTAGATCATAGCAGTGAA	16351
Wm5      ------------------------------------------------------------	12487


Wm1      TGGACTGGATTTTGTGAGTATAGAATTATTATAAGAAGCACCACTCACCCTTCATATCTG	16411
Wm5      ------------------------------------------------------------	12487


Wm1      AGAGCTATTTGGTATACGATGAAGACGAATACACTGATTGCGATACCAGCAGGTGATCTG	16471
Wm5      ------------------------------------------------------------	12487


Wm1      TACCAAATGGAAGTACCGAAGAAACAAAGTGTACTGCCAATGTACATTGGATCCTCAACA	16531
Wm5      ------------------------------------------------------------	12487


Wm1      ACATTGAATGGGAAACTTGTAACGCGGTGGCTCATAAGTATACCAAAGTAGTCGCCCAAG	16591
Wm5      ------------------------------------------------------------	12487


Wm1      TAGGTTCCAGTAACGCCTAACGCAAGCATAGAAGTTATAACTAAGATTTGGCCAGAGAGG	16651
Wm5      ------------------------------------------------------------	12487


Wm1      AACAAAGCGAGAGCAACGTATACATGCGCAGTTGGGTAGTATGGATGTGGAGCCAAGTTT	16711
Wm5      ------------------------------------------------------------	12487


Wm1      GGTTGTTCAGCTAAAGCACTCTGAAACCTAGAAAGATGAGAAAGAAGTTCTACTATAATA	16771
Wm5      ------------------------------------------------------------	12487


Wm1      CTGCACGTACAGATGATCACGCAAGATACCTAATGAGAAAATGGTAACAGCAAGGAGGTA	16831
Wm5      ------------------------------------------------------------	12487


Wm1      ACAGCCAAGATATGGTCTACCACCAGCGAGCTTTGTGAGTAGCTTATTGTGATATTCTTT	16891
Wm5      ------------------------------------------------------------	12487


Wm1      TTCAACGTGTAAGTTCAAAACTCATGTCTAACGAGATAGTACAAACCTTGACGAGCAACC	16951
Wm5      ------------------------------------------------------------	12487


Wm1      TATGAAAAGAATCAGAATCAAAGGCTTAATAGTGTCTCTATACTCACGATATTCCAGAAT	17011
Wm5      ------------------------------------------------------------	12487


Wm1      AAGGGGTTGAATACAATTGATGCGATTACGATCCAAAGTGAAGGTTGCTTGAAATCGAAC	17071
Wm5      ------------------------------------------------------------	12487


Wm1      CAATCTTCTGGCTTGTCCTGTGAAACAGATAACAACGGTGAAGGGTGTGGAACCAGCTCA	17131
Wm5      ------------------------------------------------------------	12487


Wm1      GAAAGCCCTCTAATAAACGGATGTTGCTTAGCGACCATCGACGTAACGTGTAAAGCGAGA	17191
Wm5      ------------------------------------------------------------	12487


Wm1      GTTTTGTGCAAACCACGAGGACGAAATACTTTATAAAAACATAAGTATCCTTAGAGTCTT	17251
Wm5      ------------------------------------------------------------	12487


Wm1      TTATTAATGACAGTCGACCCTCAACAATGGCTCGACACTATCTTATCGGGAAAGTATCTT	17311
Wm5      ------------------------------------------------------------	12487


Wm1      GCTGAATCCCAAATCAAGATGTTATGCGATGTAGTACTCGGATTACTGGTAGAGGAATCA	17371
Wm5      ------------------------------------------------------------	12487


Wm1      AATATACAACCCGTATCAAGCCCTGTCACCGTTTGCGGAGATATACACGGTCAGTTTTGG	17431
Wm5      ------------------------------------------------------------	12487


Wm1      GATTTACTGGAGCTTCTGCGGGTCGGTGGTGATTGCAGGGATACCAACTACATTTTCATG	17491
Wm5      ------------------------------------------------------------	12487


Wm1      GTTAGGAAACAAACTTATTAATAAATATGATGCTGATTATGGGTAGGGTGACTTTGTTGA	17551
Wm5      ------------------------------------------------------------	12487


Wm1      TAGGGGTCATCAAAGTTTAGAGAGTTTTACCCTTCTTATGGCCCTCAAAGCCAGGTTGGT	17611
Wm5      ------------------------------------------------------------	12487


Wm1      TGACATAATTCTATTATGGTTCATCTAACAGCTATAAGGTATCCTGATAGGATAACTCTA	17671
Wm5      ------------------------------------------------------------	12487


Wm1      CTGCGTGGTAATCATGAATCTAGGCAGATCACTCAAGTATACGGATTTTATGGTAAGCTG	17731
Wm5      ------------------------------------------------------------	12487


Wm1      GCTTCATAGTCGAGCTATAGTATTGACCGAGAGTCTAGACGAATGTCAACAAAAGTATGG	17791
Wm5      ------------------------------------------------------------	12487


Wm1      CAATGCAAGTGTATGGAAAGCATGCTGTCAAGTCTTCGACTATCTCAATTTAGCTGCTGT	17851
Wm5      ------------------------------------------------------------	12487


Wm1      AAGTCTAATGCCATTCAATTAGTCACAGGCTGAATGGCCAAAGATCATTGATGGTAAAAT	17911
Wm5      ------------------------------------------------------------	12487


Wm1      CTTATGTGTTCATGGAGGTTTGTCACCAGACGTACGAACTCTAGATCAAATTCGCGTAAT	17971
Wm5      ------------------------------------------------------------	12487


Wm1      TGGTGAGCTTCAGTTGGACCACTCAATTTAAAACTTATACACCTTAAGCACGAGCTCAAG	18031
Wm5      ------------------------------------------------------------	12487


Wm1      AGATACCTCATGATGGACCGTTTTGTGGTAAGAAAGGTATTTGTTGTGGTTTTCTCACTA	18091
Wm5      ------------------------------------------------------------	12487


Wm1      ATGCGACCATTTAGACCTCATGTGGTCGGATCCTGACGATGTAGAACATTGGAGCATAAG	18151
Wm5      ------------------------------------------------------------	12487


Wm1      TCCCAGAGGGGCTGGTTGGCTATTTGGTAGCAAAGTTACAAAAGAAGTAAGATTGGCTTT	18211
Wm5      ------------------------------------------------------------	12487


Wm1      AATTCTATGATCATATTTCAACTATATGCAGTTTAATCACATAAATGGTTTAGACATGAT	18271
Wm5      ------------------------------------------------------------	12487


Wm1      CGCCCGTGCTCATCAGCTTATTCAAGAGGGCTTGTATGTATCAAGCATCGTAGCTGATAT	18331
Wm5      ------------------------------------------------------------	12487


Wm1      TTTACTTACTCGCCTTAGCAAATTCATGTTTCCGGACAATGACCTTGTGACTGTCTGGTC	18391
Wm5      ------------------------------------------------------------	12487


Wm1      AGCACCTAATTACTGTTATAGATGCGGTTAGTTGGAACGCTGAAATTGGTCCGCAGTCAT	18451
Wm5      ------------------------------------------------------------	12487


Wm1      ACTAAATAATGACCAGGAAACGTTGCCAGCGTTCTTAAAGTGAAAGAGAATGAACCTGTC	18511
Wm5      ------------------------------------------------------------	12487


Wm1      ACAGAAAACAATTTTGCAATTTTTGATGCAGGTACGTATTTCTATGAAGAAATAATACGG	18571
Wm5      ------------------------------------------------------------	12487


Wm1      CTACTTACAACTACTATAGTAGCAGATCAGGACAGAGGTGCTGTTGCGAGAGTCGCTCCT	18631
Wm5      ------------------------------------------------------------	12487


Wm1      TCTCAATACTTGTAAGTACGCATGACCTTCTTGTATCTCACTGATATGGCCAGCTTGTAA	18691
Wm5      ------------------------------------------------------------	12487


Wm1      ATTCTGTTGTAAATTATGCGATAACTATTCATGTTTATTCTAATAACATAATTGTTTATT	18751
Wm5      ------------------------------------------------------------	12487


Wm1      CTAGAACGTGTGAATGAGGCTTCGGCCAATGGGCGATTGAAATGTTGAAAGATGACGTCA	18811
Wm5      ------------------------------------------------------------	12487


Wm1      CTAACATGCATAAAGACAAAGACAAGATAAAAAGACTACGAGATTCCAAGTTTTCAAATT	18871
Wm5      ------------------------------------------------------------	12487


Wm1      CGTCCTTGAAAACTGGAATGATCCCTTTCCACTCATGGCAGATCGTTCCGCCATCGATGT	18931
Wm5      ------------------------------------------------------------	12487


Wm1      TCACCAAAGCTTTCACAAGGCTCGGAGTCTTATATGTATCTAGCTTAGCCGAATGTAGTC	18991
Wm5      ------------------------------------------------------------	12487


Wm1      GTAAATATATAGGTATAGTGTTATAGACCATATCACTAGCTTTTGCAAAGTTAGCACTAT	19051
Wm5      ------------------------------------------------------------	12487


Wm1      TTGGACGTTTCGTCGACACCTGAGTTGCTCAGGTGTAGGCTCATTCACTAATAAAGCACG	19111
Wm5      ------------------------------------------------------------	12487


Wm1      AACTCAAGTTAATGAGGAGTACCCTCTATCCAGTCTCAAGTCAATATGTTTCCACGAGGA	19171
Wm5      ------------------------------------------------------------	12487


Wm1      CACATAAAGGAGCCCGTGAGAAGGAACATCAAATGCAATAATCATCAACTATCGCATCCG	19231
Wm5      ------------------------------------------------------------	12487


Wm1      ATTCATGGAGAAAGCTATGCGAACAAGCTCATGAAAACTTGGTAACCCCAAAAAGTAGAG	19291
Wm5      ------------------------------------------------------------	12487


Wm1      AGTTACATCTAATAGAAGGAACTAGCTTACTGACGACGAAAAAGAGGTGAATGATTGAAT	19351
Wm5      ------------------------------------------------------------	12487


Wm1      GTCCAAAGTATTAAGCTAACATTTTCAATCGCATCGGCAAGCTTATCTCAGAGAGAAAAT	19411
Wm5      ------------------------------------------------------------	12487


Wm1      AAAAGAGGCCTCTGAAGAAGTAAACCAATGATCTTGCTCTATAGTGATCATCAAATTATT	19471
Wm5      ------------------------------------------------------------	12487


Wm1      GAGAATTATATGTACTTTGCTGTTTTGTGATATCATTGAACTTTAATTGTTCTTGTTATA	19531
Wm5      ------------------------------------------------------------	12487


Wm1      TTTCATGTATATCTATTAATATTTATATTTATATTAATTCATCCAAAAAATCTTGATCAG	19591
Wm5      ------------------------------------------------------------	12487


Wm1      ACCTTGTGTCTATGGTGCTTATATTCATGGGCTGATTATCGCGTTCGATAGCGACTGTTT	19651
Wm5      ------------------------------------------------------------	12487


Wm1      CACTTTTAACGCGATGCGCTGTTTTTGTTGGTGAGCTACTTTTTGGTTCGCCCACACTTT	19711
Wm5      ------------------------------------------------------------	12487


Wm1      CAAAGAAATCGTTGTCTTCTAAATGATTCACGTCTCTCCTTGATGATTCGTATCGCTGGA	19771
Wm5      ------------------------------------------------------------	12487


Wm1      TGTATTGTCTACCTGAATCACCTCTCAGACGAGATTTAAGATCTTTAAGGATGTCAAAAG	19831
Wm5      ------------------------------------------------------------	12487


Wm1      ATGTAAAAGATTGCTGTTACTCACTCCATGATGCTTCTGCCTTCATCCTATCGTTACGCA	19891
Wm5      ------------------------------------------------------------	12487


Wm1      TCTCCTCCAATGCTTTTGAACTCTCTGAATCAATAGGAAGTAATTGGGGTTCTTTCTTCT	19951
Wm5      ------------------------------------------------------------	12487


Wm1      TGTGTCTATTAGCCAAATCATAGTCTATATCATCATCGTCTGCATCTGTAGGTCGCTTTA	20011
Wm5      ------------------------------------------------------------	12487


Wm1      TATATTGTTCAAAATCGTCATCTACCTTAAGTTTCATTGCGATGGACGAGAGATCACGCT	20071
Wm5      ------------------------------------------------------------	12487


Wm1      CTTCTTCTGATGAAACTGTGTATAGTCAGTCTAGTGGATCTATATATTCCCTCTCATACC	20131
Wm5      ------------------------------------------------------------	12487


Wm1      TTCCTCTTTCTCGGTCGCGGGTTCATCGTATTTACTGGAGTTATCAACTAATTTCCGCCT	20191
Wm5      ------------------------------------------------------------	12487


Wm1      AGAGTATTTTGGGTTCGTTGAACGCCTTGTGGCTAATAAATTAAGCTTTATCTAAATGTA	20251
Wm5      ------------------------------------------------------------	12487


Wm1      TCCCCTGTAAACTCACTGTTATTACTAGCAGATGGAACTGCAAGCTTATCTTCACTTTGA	20311
Wm5      ------------------------------------------------------------	12487


Wm1      CTAATCTCTGAGCTATCATTTTCGCTACTACTGCCATCTGAGCTATCAGATTTCTTTAAG	20371
Wm5      ------------------------------------------------------------	12487


Wm1      TCAGGAAATTCCGCTTGATTATCTACTTCTGGTGGCTGTAGACGTCGTGTGGTGGGTTTT	20431
Wm5      ------------------------------------------------------------	12487


Wm1      GGAGCGGTACTTAAACCTCTTGAAGCTTTCCATGCTTTTGAAGCTTTAGCTTTATATTTA	20491
Wm5      ------------------------------------------------------------	12487


Wm1      TCTTGTAAAACCATTCAAATTTCTTATTTTTTGACGAATTCGTCGACTTGGTTAGCAACA	20551
Wm5      ------------------------------------------------------------	12487


Wm1      GTCAAGAAATGCAAACTCAGATCCGTGAAGTCTGGGCAGATAATCTAGACGCAGAGCTAG	20611
Wm5      ------------------------------------------------------------	12487


Wm1      ATATTTTGCGTGAATTAGTCGATCGATATCCTTACGTTTCTATGGTGAATTCAATCGACG	20671
Wm5      ------------------------------------------------------------	12487


Wm1      TATTTGACATCCAAACTGACGGTTTTAGGATACTGAATTCCCTGGCGTTGTAGCTAGACC	20731
Wm5      ------------------------------------------------------------	12487


Wm1      AATTGGAAATTTTAAGAATTCATCAGATTATCACTATCAAACGCTGCGATGCAACGTAGA	20791
Wm5      ------------------------------------------------------------	12487


Wm1      TTTATTAAAGATAATACAATTAGGTATCACTATCGCTGACGCAGACGGCAATATGCCTGA	20851
Wm5      ------------------------------------------------------------	12487


Wm1      GTATCCTACTTGGCAGTTCAACTTTAAATTCAGTTTAAAGTACGCCTCTTTTATCATATC	20911
Wm5      ------------------------------------------------------------	12487


Wm1      ATTCATCATTCTAATATCTACATAGCGATGATATGTATGCACCAGATTCAATAGATTTAC	20971
Wm5      ------------------------------------------------------------	12487


Wm1      TCACAAAATCTGGTATTAATTTTAAAAGGCTAGAAGCTAATGGTATTGATATTGAAGAAT	21031
Wm5      ------------------------------------------------------------	12487


Wm1      TTGGAGAGAAACTGGTCACTTCTGGTTTTGTTCTGTTCGATCACGTTCACTGGATATCGT	21091
Wm5      ------------------------------------------------------------	12487


Wm1      TTCACAGGTGAGTAACAAGCGCGATGTAACTCGTTTACAAACTCAATATAGTGGCTATGA	21151
Wm5      ------------------------------------------------------------	12487


Wm1      CTTTGGTTACTTACTCAAAGTTCTCACTTCATTGCCCTTACCTTCAAATGAAAACGACTT	21211
Wm5      ------------------------------------------------------------	12487


Wm1      CTTTGATCTACTACGCATTTGGTTCCCTTGCATTTTTGACATAAAATACGTTATGAAAAT	21271
Wm5      ------------------------------------------------------------	12487


Wm1      CAATAGGCTGTTAAAGGGTGGTTTACAAGATATTGCAGATGAATTACAGGTAATAATAAG	21331
Wm5      ------------------------------------------------------------	12487


Wm1      CATGTTCCATTATATCAGCTGCTCTGACTCTCATTAGGTGATGCGAATAGGCCCTCAACA	21391
Wm5      ------------------------------------------------------------	12487


Wm1      TCAAGCAGGTTCAGACTCCTTAGTCACATCAGCTTCCTTCTTCTCCATGCGTACAAAATT	21451
Wm5      ------------------------------------------------------------	12487


Wm1      CTTCGATGGTACATCTGAAAGAGATAGACAATTGTAAGACATCCTTTCCGAAACTTATTA	21511
Wm5      ------------------------------------------------------------	12487


Wm1      CTTTGCTAATTTCACTATCAGACCTCATTGTATGGGATATCTTTATGGTATAGGTATGGG	21571
Wm5      ------------------------------------------------------------	12487


Wm1      CGCTGGAGCAACTGCTATACAAACCCCAGGGGCACCTTTCACACCAATGAGCAATCCAAT	21631
Wm5      ------------------------------------------------------------	12487


Wm1      CCCAACTCCTTACGTTGGTCCTCAACCCATAGCACCTTATCCAAATCGAACAAACGTCGG	21691
Wm5      ------------------------------------------------------------	12487


Wm1      ATTCGGAGAAAGGTAAATTAAATTGTATTTTGTACATTATCATAAATCATTTCTATTTCC	21751
Wm5      ------------------------------------------------------------	12487


Wm1      AGAATTAACAGAAAATTTTATGAATTCGAAATTCATGCAACATAGTGATCGAGGCCGAAT	21811
Wm5      ------------------------------------------------------------	12487


Wm1      AAAGTTCCCATTGTATACAAAATACAATTAAAATAAATCCACAAGCTACTCCTTAGATTC	21871
Wm5      ------------------------------------------------------------	12487


Wm1      CTTGAACATCTCATATTAATCTAATCGAAAATGTTCCCTTCTTGGCTTTTTGAGCTGCTG	21931
Wm5      ------------------------------------------------------------	12487


Wm1      TCTATACTGGCTATTGCGTCAGTGTTGATACCAGCACCATTCCATATTAAAGCACAGAAT	21991
Wm5      ------------------------------------------------------------	12487


Wm1      TATGCCGCTGTCGCCCTATTAAGTTGGCTGTTGCTTGACAATATTTTCAAAACAGTCAAT	22051
Wm5      ------------------------------------------------------------	12487


Wm1      AGCTTTGTTTGGAAAGGTAACGTAGACATCAAAATTCCAGTCTGGTGTGACATTGGTAAG	22111
Wm5      ------------------------------------------------------------	12487


Wm1      TGAAGAATCGATAATCAACCTCTCGTTTTAACAATTTAGGAATTAAAATCAATATTGGAG	22171
Wm5      ------------------------------------------------------------	12487


Wm1      GTTCGATAGCCTTACCTGCTGCGTGCCTCTGTATCAATCGCTTTCTTGCAAGCATCTCGT	22231
Wm5      ------------------------------------------------------------	12487


Wm1      CTTCCGATACAATTAGAGCTACAAAGAAAGAGAGGAGAAACCGTCTATTGTTTGACCTTT	22291
Wm5      ------------------------------------------------------------	12487


Wm1      CAATGTGTTACGGCCTACCGGTACTATTCATGATACTTCATGTTGTTTTTCAAGGCCATC	22351
Wm5      ------------------------------------------------------------	12487


Wm1      GCTTCAACATTATTGAAAATATCGGTTGTTCTCAATATACATACAACAATTGGGGAAGTA	22411
Wm5      ------------------------------------------------------------	12487


Wm1      TTATTGTATTCAATATCTTACCATTAGTCCTAGCTGTTTTATCAGTGTTCTATGGATGTA	22471
Wm5      ------------------------------------------------------------	12487


Wm1      AGTCTAGCTATAGTCTTTAGCTGACGTGCTGACAACATACAGCCTATGCCAGTTTTCACT	22531
Wm5      ------------------------------------------------------------	12487


Wm1      TGCTTAAACGAAGATACCAATGTGATGATGAATTTTTAAGGGATTCGACGAATTTCACCC	22591
Wm5      ------------------------------------------------------------	12487


Wm1      CCAGTAGATTCTATAGACTACTAGGGTTGACGTCAACCTTAATACTAATCAACCTTGCTT	22651
Wm5      ------------------------------------------------------------	12487


Wm1      TCAACATAATGGGTATGATATTCAGCGTCACGTCTTACATAGGTTCTTATGAGAGCTGGG	22711
Wm5      ------------------------------------------------------------	12487


Wm1      GTTACGTCCACAAAAATTTCTCACATATTAGCCAATTAGGTCTTGAGGTATTATCGCAAA	22771
Wm5      ------------------------------------------------------------	12487


Wm1      AGGCAATCATACGAATCCAAATTATCTGGTTACTATCTCCAGTTGCAAGTTATATATTCT	22831
Wm5      ------------------------------------------------------------	12487


Wm1      TTGCGTTCTTTGGATTTGGATTTGAAGCGAGGAAAGAATATCTATCATGGTATCAAGGCG	22891
Wm5      ------------------------------------------------------------	12487


Wm1      TCAAGATGTTAATAATCTGGTGTTATGAAAAATTTACGAAGAAATCTGTCTCTAAAACAT	22951
Wm5      ------------------------------------------------------------	12487


Wm1      TCAATCCACCAACCTATGAAGATTCCAGGTATTCAAATGGAATTGATTATTCCCTCAAAG	23011
Wm5      ------------------------------------------------------------	12487


Wm1      ATATTACTGGTAAAACAGGAGTAGACTTTGATCAATATGAGCTTCCCTTCCTAACTAACT	23071
Wm5      ------------------------------------------------------------	12487


Wm1      CACACGACTCCCATACAGTCAAGGTATGAAAAGGAGAGGTTCAAAATAGACTTTGTAATT	23131
Wm5      ------------------------------------------------------------	12487


Wm1      TAGATTATTAATGTAAAAGCCATGTATTAAATTTATACTTGATCTTCATCGCGACTGAGG	23191
Wm5      ------------------------------------------------------------	12487


Wm1      ATGTGTAAGAGAAGAGTTAGGATAGACAGTAAAGACGTACCCAGCGTAGAATTTTTCAAG	23251
Wm5      ------------------------------------------------------------	12487


Wm1      GAAAGTTTGTGGTCTAGCTGTGAGTATTATGTTAGTTTTGAATAAATAATAAAGTTATCA	23311
Wm5      ------------------------------------------------------------	12487


Wm1      AACTGACGTGGTGCCTGTGTAAAGTTAGATATAGCGAATGAGAGTAGAAAGATTACTCAC	23371
Wm5      ------------------------------------------------------------	12487


Wm1      TTCTTCCAATGCAAACCAGCGCATAATGTGAGTTTCTTGCTTCCATGCGTTCATTGTTTC	23431
Wm5      ------------------------------------------------------------	12487


Wm1      TTGGTATTCCTGTCTACCTTTGAATAAAACTAAGTCTATCACATTCAAATCGTCGAGATT	23491
Wm5      ------------------------------------------------------------	12487


Wm1      TCTGTTCTTTTCAAACTCCTGACGAATTTTTGCTCTAATCTGTGATGGTGGAATATTTAA	23551
Wm5      ------------------------------------------------------------	12487


Wm1      TGAATAAAGCGCACAGATACTTGGTGCCTATAAGTATATCTTAATAAAGCAATCAACCAT	23611
Wm5      ------------------------------------------------------------	12487


Wm1      CATCTATTCTATCACTCACGCTACGATAAAAGTCACGATAAAGAGTTCTAGCTCTAGCGT	23671
Wm5      ------------------------------------------------------------	12487


Wm1      GAGCTTCTGCGAATGAAGTTGACGATTTTGTTACGTGTGCCAATCTTGAAGGAATAGTAG	23731
Wm5      ------------------------------------------------------------	12487


Wm1      TCATCGTCGGGAATCAAGAGATAATAAGAAGCTAGAACTAATAGATAAAATCTGTAATAG	23791
Wm5      ------------------------------------------------------------	12487


Wm1      TATAGTTTATTCAATTAATCGTTCATATTTATTTATTCATACGATATATCTCTTCATCTT	23851
Wm5      ------------------------------------------------------------	12487


Wm1      CGTCCTCGATGTCCGCTAAGATACCCAGTACAGCGTTATACGCAAATCAAATTGAAAGCC	23911
Wm5      ------------------------------------------------------------	12487


Wm1      CACCAAGGCTCAATGGTAGTCTAACGAACTCTGCCTTTTCTATATCTCTTATTGCTTTTA	23971
Wm5      ------------------------------------------------------------	12487


Wm1      CCTTAGGTGCTGTCTTTGGTTTGAGCTTACTTCCCGGTTTATCAGTTATCAACTTAGATT	24031
Wm5      ------------------------------------------------------------	12487


Wm1      TGAGTGCTATAATTCCAACAGCATTACATTCAACCCAAGTTTCCCTATATTTAGTCATAC	24091
Wm5      ------------------------------------------------------------	12487


Wm1      TTTGTTGGTTTCATCTTTGGGAATTCCTCAGTACAGCAGGTTGGAATATGACAAAGACAT	24151
Wm5      ------------------------------------------------------------	12487


Wm1      CAGTCGATTGTTAGTCATATACTTAATTGAATATGCAATCTAATAAACTAACAGCTTACT	24211
Wm5      ------------------------------------------------------------	12487


Wm1      TATTAAACAATGGATATAGCTACATCGCTGCACACGCTTTCGGTCTAGTGTCTCATTACC	24271
Wm5      ------------------------------------------------------------	12487


Wm1      TTTTGATATTGTTCTACCCTTCAATTTCTCAAAATCATTACTTAATCATCTCAGGTAAGT	24331
Wm5      ------------------------------------------------------------	12487


Wm1      GTGGTATGAACCATTTAATTGCCCTTACCATAACTTAAGGTCTCGCCTTGGTTGTCGTAG	24391
Wm5      ------------------------------------------------------------	12487


Wm1      GTCAGGTCTTGCGTACGCTTGCTATGGTACACGCGAGTGAAAGCTTTTCACATCATGTCG	24451
Wm5      ------------------------------------------------------------	12487


Wm1      CATCCTTTAAACAAAAAGATCATAAGTTAGTGACTAATGGCGTTTACAGGTAAGAGTATT	24511
Wm5      ------------------------------------------------------------	12487


Wm1      TTGCTATATAATATTTTTCATTAAACTTTCACAGAATCGTACGACACCCTTCGTATTTAG	24571
Wm5      ------------------------------------------------------------	12487


Wm1      GTTTCTACCTATGGGCATTGGGAACACAGATGATTTTGACAAATCCTTTATCATTCATAG	24631
Wm5      ------------------------------------------------------------	12487


Wm1      CATTTGCTATTATTCTGCATAGATTTTTCACTGATAGAATTAAACGTAAGTTTTTCTCTT	24691
Wm5      ------------------------------------------------------------	12487


Wm1      TTCCTCACTTAAGACATTCTTCTAATTCTCGCAGATGAAGAAACTTATCTCGTAAAGTTC	24751
Wm5      ------------------------------------------------------------	12487


Wm1      TTCGGAAATGACTATCAATCTTATAAAAATAATGTGTACTCCTTTTTCCCGTATATCATA	24811
Wm5      ------------------------------------------------------------	12487


Wm1      TGAATATTCTTCGAATGGTATCTTTATCAAACTTGGTAATATGAATGGTACGGGCACAAG	24871
Wm5      ------------------------------------------------------------	12487


Wm1      TACTAAGAAGCTAAT	24886
Wm5      ---------------	12487
```

**Alignment of downstream regions**

```
Wm1      GACCGCTAAGTGAATGGAACAACGATGGGTACAACAAAAAAAGAAAAGAGAATAAGTAAT	60
Wm5      GACTGCTAAGTGAATGGAACAACGATGGGTACAACAAAAAAAGAAAAGAGAATAAGTAAT	60
         *** ********************************************************

Wm1      TGCGAAGAGCAGAGGAAATGGGTGCTGTAGGGAGATATTACAAAGCTCTGCTGTGCGACT	120
Wm5      TGCGAAGAGCAGAGGAAATGGGTGCTGTAGGGAGATATTACAAAGCTCTGCTGTGCGACT	120
         ************************************************************

Wm1      GAAGTGAATGGTTAGTGGAAATTTGAAATCCAGATATTAACATACTTAGAAGATTAGTTG	180
Wm5      GAAGTGAATGGTTAGTGGAAATTTGAAATCCAGATATTAACACACTTAGAAGATTAGTTG	180
         ****************************************** *****************

Wm1      TTATCCACTTTTGTGAGTCATATCATAGAAATAGAGATGATGAATGCAGTGTTATATATA	240
Wm5      TTATCCACTTTTGTGAGTCATATCATAGAAATAGAGATGATGAATGCAGTGTTATATATA	240
         ************************************************************

Wm1      TTTTCTATTGATAAGATGAAGTTTGATTGATGTGTTTTCTTTGTTCATATGCTTCCATAT	300
Wm5      TTTTCTATTGATAAGATGAAGTTTGATTGATGTGTTTTCTTTGTTCATATGCTTCCATAT	300
         ************************************************************

Wm1      CGTGCGTGTAATTTGTTATATATAAAAAAAGAATCCTTTTTTTTGTGAGTAAAACTCATT	360
Wm5      CGTGCGTGTAATTTGTTATATATAAAAAAAGAATCCTT-TTTTTGTGAGTAAAACTCATT	359
         ************************************** *********************

Wm1      TAGTTGATTGTGATGAATTATTTCTTTGATGTTGTTTATCTTTGTTTGACTTCTTTTTAG	420
Wm5      TAGTTGATTGTGATGAATTATTTCTTTGATGTTGTTTATCTTTGTTTGACTTCTTTTTAG	419
         ************************************************************

Wm1      AATTATTCGACTTATTTTTACCTGTTTCGCTACCAACACTAGCAGACCTACGTTTTTCTT	480
Wm5      AATTATTCGACTTATTTTTACCTGTTTCGCTACCAACACTAGCAGACCTACGTTTTTCTT	479
         ************************************************************

Wm1      TATTATTCTGTTGTTGCTGTTGTTGCTTCTTCCGTGATGCTTGTAAACTCGCTGAGTAGA	540
Wm5      TATTACTCTGTTGTTGCTGTTGTTGCTTCTTCCGTGATGCTTGTAAACTCGCTGAGTAGA	539
         ***** ******************************************************

Wm1      GGTTATTCAGGCTTGAAGAAGTCTTGAATTGTCCTTGTGAATTGTTATTACTCAATGCGG	600
Wm5      GGTTATTCAGGCTTGAAGAAGTCTTGAATTGTCCTTGTGAATTGTTATTACTCAATGCGG	599
         ************************************************************

Wm1      GAAAACCTTCTTCTAATCCAGTCGTCAAATTTGGTTTAAACGGTGGTGTATCCTCATCGA	660
Wm5      GAAAACCTTCTTCTAATCCAGTCGTCAAATTTGGTTTAAACGGTGGTGTATCCTCATCGA	659
         ************************************************************

Wm1      CATTGGTTGCATCACCAGCTTTATCATCTAACAGAGGTAAAGTTGCGTTTGGAATTATTC	720
Wm5      CATTGGTTGCATCACCAGCTTTATCATCTAACAGAGGTAAAGTTGCGTTTGGAATTATTC	719
         ************************************************************

Wm1      TTGAATACGTTCGGCCACCACCTACTGACTTCTCAAACTCTCTACTTGATATTCTAGACG	780
Wm5      TTGAATACGTTCGGCCACCACCTACTGACTTCTCAAACTCTCTACTTGATATTCTAGACG	779
         ************************************************************

Wm1      TTTTGTTGGATACTCTCGGAGTTGAAGGTACTGAACGTGATCGAGTAGGTAATTTACGAC	840
Wm5      TTTTGTTGGATACTCTAGGAGTTGAAGGTACTGAACGTGATCGAGTAGGTAATTTACGAC	839
         **************** *******************************************

Wm1      CCGTTGTAGTTGATGGAGTTGTTGCCGTTGTTGTTGTTGCTTTTGAAGGCGTTTGTGTAT	900
Wm5      CCGTTGTAGTTGATGGAGTTGTTGCCGTTGTTGTTGTTGCTTTTGAAGGCGTTTGTGTAT	899
         ************************************************************

Wm1      TCCTGGCAGCAGCTTGTTTCTGCAATTTAAGCCAACGTCTTGCTGACATCCTTGGTGGTG	960
Wm5      TCCTGGCAGCAGCTTGTTTCTGCAATTTAAGCCAACGTCTTGCTGACATCCTTGGTGGTG	959
         ************************************************************

Wm1      GTACACGTACATCAGCTGCCAGAGTTAATGCAGACGTAGTTAGACTTTCCGAAGCGACGA	1020
Wm5      GTACACGTACATCAGCTGCCAGAGTTAATGCAGACGTAGTTAGACTTTCCGAAGCGACGA	1019
         ************************************************************

Wm1      AAGTGTTCTGACCGAATGTATAACGTTGTGCTTGTGGGAATGGAGGGAATCCCAATCCTA	1080
Wm5      AAGTGTTCTGACCGAATGTATAACGTTGTGCTTGTGGGAATGGAGGGAATCCCAATCCTA	1079
         ************************************************************

Wm1      GTCCTACTGTTTGCTCTTTCTCTTCTCCATTTCTAGAAGGTTGAGCGGTTGATGTTACAG	1140
Wm5      GTCCTACTGTTTGCTCTTTCTCTTCTCCATTTCTAGAAGGTTGAGCGGTTGATGTTACTG	1139
         ********************************************************** *

Wm1      GCGCACTCAATGTAGATGTTGTCGACAAAGGTGGTGAATTTGTAGGTGTACCAGACTCAG	1200
Wm5      GCGCACTCAATGTAGATGTTGTCGACAAAGGTGGTGAATTTGTAGGTGTACCAGACTCAG	1199
         ************************************************************

Wm1      ACGGTTGAGTGAATGTTGACGGTGTTATTCTAATTTGCTGACCAGCTCCATTTGTAACAA	1260
Wm5      ACGGTTGAGTGAATGTTGATGGTGTTATTCTAATTTGCTGACCAGCTCCATTTGTAACAA	1259
         ******************* ****************************************

Wm1      ACGTCGGCACCCGTATTTTCATATCACTGTTATTAGCTGCTATTTGTTTTTGGATCAATT	1320
Wm5      ACGTCGGCACTCGTATTTTCATATCACTGTTATTAGCTGCTATTTGTTTTTGGATCAATT	1319
         ********** *************************************************

Wm1      GTGAAGGTGAAGCATTCACACTATTAGCGGTTGTTGCTGCTGCAGCTACCGCTGCTGCGC	1380
Wm5      GTGAAGGTGAAGCATTCACACTATTAGCGGTTGTTGCTGCTGCAGCTACCGCTGCTGCGC	1379
         ************************************************************

Wm1      TGCTCGATGCACGTGAATGTAAAGCAGCATTGGCTAATCTCGGAGAATGCCTCAATGGTG	1440
Wm5      TGCTCGATGCACGTGAATGTAAAGCAGCATTGGCTAATCTCGGAGAATGCCTCAATGGTG	1439
         ************************************************************

Wm1      CAGGTATTGGTACTGGTGGCCATCCAATTTCAGCTTGAGTTGTTGCATCAGCGTAGGGCC	1500
Wm5      CAGGTATTGGTACTGGTGGCCATCCAATTTCAGCTTGAGTTGTTGCATCAGCGTAGGGCC	1499
         ************************************************************

Wm1      CTGAAACATTAGGAATTGTAGCTTTGAGATGATGTTTTGGTGCATACTTCCTCTCATGTA	1560
Wm5      CTGAAACATTAGGAATTGTAGCTTTAAGATGATGTTTTGGTGCATACTTCCTCTCATGTA	1559
         ************************* **********************************

Wm1      ATCTAGATAACCAAGTTGGTCCCACGTTTGCACCTGAACTAGCTCTTCTTGGTATACTAC	1620
Wm5      ATCTAGATAACCAAGTTGGTCCCACATTTGCACCTGAACTAGCTCTTCTTGGTATACTAC	1619
         ************************* **********************************

Wm1      TACTTTGTTCAGTTCTTGTGGATTCATCTGAAACTAAATTTTCTGCTTCCCAGAAAGCAC	1680
Wm5      TACTTTGTTCGGTTCTTGTGGATTCATCTGAAACTAAATTTTCTGCTTCCCAGAAAGCAC	1679
         ********** *************************************************

Wm1      TACCTAATGGTTTAGTATGTGACTGAGGATGTTGATACATCAGATGCTCTTCATGTGCGT	1740
Wm5      TACCTAATGGTTTAGTATGTGACTGAGGATGTTGATACATCAGATGCTCTTCATGTGCGT	1739
         ************************************************************

Wm1      TCCTCATCCTTTCATTTTGAGCAACATTTAATGCTATTGATAACGCTTGAGCATGTGATG	1800
Wm5      TCCTCATCCTTTCATTTTGAGCAACATTTAATGCTATTGATAACGCTTGAGCATGTGATG	1799
         ************************************************************

Wm1      ATGATTGTCTACGTCTATTAAATGTAGCTGGTGGTGATGATATTTGATCGCTGACTTTTC	1860
Wm5      ATGATTGTCTACGTCTATTAAATGTAGCTGGTGGTGATGATATTTGATCGCTGACTTTTC	1859
         ************************************************************

Wm1      TTGAAGGGAAGATTGAGGATTGCGATTGAGTAGGTGAATATGATTGTTGAGATGCAGTTT	1920
Wm5      TTGAAGGGAAGATTGAGGATTGCGATTGAGTAGGTGAATATGATTGTTGAGATGCAGTTT	1919
         ************************************************************

Wm1      GAGTCATATGCGTATTGTGAGAATGCGTTTGTACAATTGGAGGTGGTTGTAAGCTAGGTG	1980
Wm5      GAGTCATATGCGTATTGTGAGAATGCGTTTGTACAATTGGAGGTGGTTGTAAGCTAGGTG	1979
         ************************************************************

Wm1      GAGGATGTAATGAAGGTCCTAGCGTTGGTCCTAAAGATGGGTTCGTGAGAGGTCTTGGTG	2040
Wm5      GAGGATGTAATGAAGGTCCTAGCGTCGGTCCTAAAGATGGGTTCGTGAGAGGTCTTGGTG	2039
         ************************* **********************************

Wm1      TGCGATTTCCAGCGGAGAAAGGAGCAGGCACGAGGGCTTCTTCTCTTTCCTCACATAGAT	2100
Wm5      TGCGATTTCCAGCGGAGAAAGGAGCAGGTACGAGGGCTTCTTCTCTTTCCTCACATAGAT	2099
         **************************** *******************************

Wm1      CTACGAGTACTTGTGAAGGTAGAATATCGGTTTTTCCTACAGTTTGTAACATCCTTGAAG	2160
Wm5      CTACGAGTACTTGTGAAGGTAAAATATCGGTTTTTCCTACAGTTTGTAACATTCTTGAAG	2159
         ********************* ****************************** *******

Wm1      CACGCATGAACTCGCCACGGATTGTGTATAAACCTGTGGTTGTTAGGAACGTTTCATTCA	2220
Wm5      CACGCATGAATTCGCCACGGATTGTGTATAAACCTGTGGTTGTTAGGAACGTTTCATTCA	2219
         ********** *************************************************

Wm1      AAATTTTCTAAAACCCACCATCAGCTGTTACCGTTCTAGCTACGTTATAGGTAGTATCAA	2280
Wm5      AAATTTTCTAAAACCCACCATCAGCTGTTACCGTTCTAGCTACGTTATAGGTAGTATCAA	2279
         ************************************************************

Wm1      ATGGATCCTCTATGCAAAGTCTATTCCTATCACGAGCGAATTCACTGGCACCATCGACTT	2340
Wm5      ATGGATCCTCTATGCAAAGTCTATTCCTATCACGAGCGAATTCACTGGCACCATCGACTT	2339
         ************************************************************

Wm1      CTAAATCTTGCACCCAACCTTTAGCGTCCTTAGAGAGAATACCACCTTCAGTTCTAATAG	2400
Wm5      CTAAATCTTGTACCCAACCTTTAGCGTCCTTAGAGAGAATACCACCTTCAGTTCTAATAG	2399
         ********** *************************************************

Wm1      AGATAACATCGTGGGTATAGTTAAAATCTTTTGCGTAGAATCTAAAGAATTCCCATAATA	2460
Wm5      AGATAACATCGTGGGTATAGTTAAAATCTTTTGCGTAGAATCTAAAGAATTCCCATAATA	2459
         ************************************************************

Wm1      ATTCACCGACAGATTGAGTATTAGCGGATGACCATTCCTGACGAAGTAAAGCAACATCAT	2520
Wm5      ATTCACCGACAGATTGAGTATTAGCGGATGACCATTCCTGACGAAGTAAAGCAACATCAT	2519
         ************************************************************

Wm1      CGAAGAAGTAAATATTTCTACCTTCATAACTTGCTGATTCTGGGCTTACTGGTCGTAATG	2580
Wm5      CGAAGAAGTAAATATTTCTACCTTCATAACTTGCTGATTCTGGGCTTACTGGTCGTAATG	2579
         ************************************************************

Wm1      GAGGTATTCGCTGTAAGTTTGGTAAAACGGGTGGTTGTTTAACGTGAGCCAAGAAGAAGA	2640
Wm5      GAGGTATTCGCTGTAAGTTTGGTAGAACGGGTGGTTGTTTAACGTGAGCCAAGAAGAAGA	2639
         ************************ ***********************************

Wm1      TGACAAGAAGGGTGAATCCGTAACTACTAAGCGTTCCCCTGTATGCGCTATTAATTTTAC	2700
Wm5      TGACAAGAAGGGTGAATCCGTAACTACTAAGTGTTCCCCTGTATGCGCTGTTAATTTTAC	2699
         ******************************* ***************** **********

Wm1      GCCTTTTACTCCAAACTTTTATGAAAAGGACGAGGGTTCTCAAACGAGGAGGGTCTATGG	2760
Wm5      GCCTTTTACTCCAAACTTTTATGAAAAGGACGAGGGTTCTCAAACGAGGAGGGTCTATGG	2759
         ************************************************************

Wm1      AGGCGTAACCAAGAAGTAAGCGAGTATTTTCTAATGCTAGCTGACCACCAAAGCCGATAT	2820
Wm5      AGGCGTAACCAAGAAGTAAGCGAGTATTTTCTAATGCTAGCTGACCACCAAAGCCGATAT	2819
         ************************************************************

Wm1      CACATGCTATACCATACGGCAGAGCAGGAGTGGGTGCCAAGGTGAGCTTTATGATAGGAA	2880
Wm5      CACATGCTATACCATACGGCAGAGCAGGAGTGGGTGCCAAGGTGAGCTTTATGATAGGAA	2879
         ************************************************************

Wm1      TACGAGCTTTCGGGAGTGGTTTGACGTGAAAGTTAGTTTCTATTGACTATTAAAAGCGAA	2940
Wm5      TACGAGCTTTCGGGAGTGGTTTGACGTGAAAGTTAGTTTCTATGGACTATTAAAAGCGAA	2939
         ******************************************* ****************

Wm1      ATAACAAAATAAAGTGCCTACCATGTCTAATTAATTCGCCAACTATCGTCGTTAGTTCTG	3000
Wm5      ATAATAAAATAAAGTGCCTACCATGTCTAATTAATTCGCCAACTATTGTCGTTAGTTCTG	2999
         **** ***************************************** *************

Wm1      AGGATGACAGTGGTTCAGACGCCGGGTCTAGAATGCATTGTAGATCCTGTAAATTGTTAT	3060
Wm5      AGGATGACAGTGGTTCAGACGCCGGGTCTAGAATGCATTGTAGATCCTGTAAATTGTTAT	3059
         ************************************************************

Wm1      TAGACGAATAAATTGTCGTTAATTAATAAGATACCATATCTGTAGTCGTTAGAAAGCGTC	3120
Wm5      TAGACGAATAAATGGTCGGTAATTAATAAGATACCATATCTGTAGTCGTTAGAAAGCGTC	3119
         ************* **** *****************************************

Wm1      ACAACGTCCGCAAAAGACACCTACCTGAATTCCTCAAAGCGAAACCGTTTGCCATAGAAC	3180
Wm5      ACAACGTCCGCAAAAGACACCTACCTGAATTCCTCAAAGCGAAACCGTTTGCCATAGAAC	3179
         ************************************************************

Wm1      CAAAAGCTATTAGTCTTGCACCGGGACTGACTCTATCTATCAACTTCTCTAATAACAGCC	3240
Wm5      CAAAAGCTATTAGTCTTGCACCGGGACTGACTCTATCTATCAACTTCTCTAATAACAGCC	3239
         ************************************************************

Wm1      TCGTCTGTTCTTTTATCGCGTACTCTTCTGCAGTCGGGAGAAGAGGCAGTATGAAACTGA	3300
Wm5      TCGTCTGTTCTTTTATCGCGTACTCTTCTGCAGTCGGGAGAAGAGGCAGTATGAAACTGA	3299
         ************************************************************

Wm1      ATAAGCAGCCAGTTAGGTCCTGTGACAGAGCTTCCCTCATTGAGTTAGAAGCCCAATTAT	3360
Wm5      ATAAGCAGCCAGTTAGGTCCTGTGACAGAGCTTCCCTCATTGAGTTAGAAGCCCAATTAT	3359
         ************************************************************

Wm1      TCGCCA-	3366
Wm5      TCGCCAT	3366
         ******
```
